# The genetics of monogenic intestinal epithelial disorders

**DOI:** 10.1007/s00439-022-02501-5

**Published:** 2022-11-23

**Authors:** Stephen J. Babcock, David Flores-Marin, Jay R. Thiagarajah

**Affiliations:** Division of Gastroenterology, Hepatology and Nutrition, Boston Children’s Hospital, Harvard Medical School, Enders Rm 605, 300 Longwood Ave, Boston, MA 02115 USA

## Abstract

Monogenic intestinal epithelial disorders, also known as congenital diarrheas and enteropathies (CoDEs), are a group of rare diseases that result from mutations in genes that primarily affect intestinal epithelial cell function. Patients with CoDE disorders generally present with infantile-onset diarrhea and poor growth, and often require intensive fluid and nutritional management. CoDE disorders can be classified into several categories that relate to broad areas of epithelial function, structure, and development. The advent of accessible and low-cost genetic sequencing has accelerated discovery in the field with over 45 different genes now associated with CoDE disorders. Despite this increasing knowledge in the causal genetics of disease, the underlying cellular pathophysiology remains incompletely understood for many disorders. Consequently, clinical management options for CoDE disorders are currently limited and there is an urgent need for new and disorder-specific therapies. In this review, we provide a general overview of CoDE disorders, including a historical perspective of the field and relationship to other monogenic disorders of the intestine. We describe the genetics, clinical presentation, and known pathophysiology for specific disorders. Lastly, we describe the major challenges relating to CoDE disorders, briefly outline key areas that need further study, and provide a perspective on the future genetic and therapeutic landscape.

**Supplementary Information:**

The online version contains supplementary material available at 10.1007/s00439-022-02501-5.

## Introduction

Diarrhea and malabsorption in infancy are a relatively common occurrence and can usually be ascribed to a variety of acquired or maternally derived causes such as congenital infections and food protein intolerance. Monogenic intestinal epithelial disorders, also known as congenital diarrheas and enteropathies (CoDEs), are a rare cause of severe, life-threatening diarrhea, and describe a heterogenous group of disorders that result from single gene variants that directly alter epithelial function. These disorders are characterized by neonatal or infantile-onset diarrhea and malabsorption and in general require intensive medical support including intravenous nutrition and fluids. Many of these disorders are associated with altered function in other organ systems, including the immune system; however, disruption of intestinal epithelial structure and function are a universal feature that define this group of genetic disorders. Here, we provide an overview of CoDE disorders, including the advances in their diagnosis and cell biology, placing them in context with other genetic disorders causing intestinal disease. We review in detail archetypal CoDEs as well as recently discovered genes and outline some of the major challenges and opportunities in the field.

*A brief history of CoDE disorders:* Infants who exhibit severe and persistent intestinal fluid loss and malabsorption requiring intensive care level support from birth or in the first few weeks of life are a rare event. These infants often require a high level of support to maintain life and to undergo further evaluation and diagnosis. Prior to the advent of the genomic era in research and clinical medicine, infants who survived past the neonatal period were often grouped together with a diagnosis of “intractable diarrhea of infancy”. This term was popularized by Avery et al. in their description of series of infants with chronic diarrhea of unknown etiology (Avery et al. 1968). The first descriptions of infants and children with chronic diarrhea of unknown etiology suggestive of a non-acquired heritable etiology were reported by Gamble et al. and Darrow in 1945 (Darrow 1945; Gamble et al. 1945). They described an unusual high-volume watery diarrhea, with high fecal chloride, hypochloremia, and metabolic alkalosis. In the 1960s, a series of cases in Finland (Perheentupa et al. 1965; Holmberg et al. 1977; Luaniala et al. 1968) led to the recognition that this disorder, termed congenital chloride diarrhea, was genetic, inherited in an autosomal recessive manner, and likely due to a single gene defect. The causative gene involved was eventually identified in 1996 as the chloride–bicarbonate transporter *SLC26A3*. Similarly in 1978, Davidson et al. described a detailed case series of five infants with persistent severe diarrhea from birth without evidence of inflammation or immune deficits but with evidence of heritability based on similar disease in related siblings (Davidson et al. 1978). The authors found clear epithelial abnormalities on examination of intestinal biopsies and postulated that the patients “evidently suffered from a congenital enteropathy which caused profound defects in their capacity to assimilate nutrients”, commenting that “the pathogenesis of this disorder, if indeed it is a single disease, remains obscure”. Several years later, Cutz et al. examined the intestinal epithelial ultrastructure via electron microscopy of one of these patients along with several more cases of severe infantile-onset diarrhea, identifying unique “intracytoplasmic inclusions composed of neatly arranged brush-border microvilli” terming the disorder microvillus inclusion disease (Cutz et al. 1989). The same group later also reported the first histological description of the characteristic epithelial “tufts” found in the CoDE disorder congenital tufting enteropathy (Reifen et al. 1994).

**Fig. 1:**
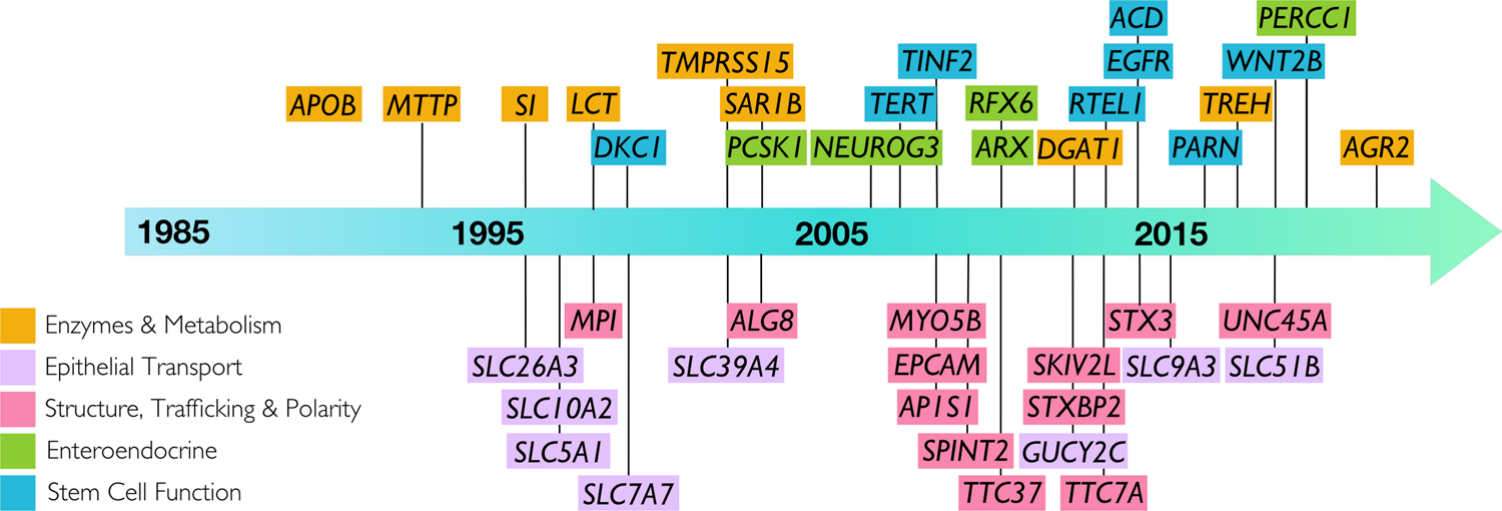
Timeline of the association of genes important for epithelial function and intestinal disease. Colors indicate CoDE disorder classification

Although a number of identified CoDE disorders were well described to be familial with classical Mendelian inheritance patterns, the causative genes, as with many monogenic disorders, were not identified until the late 1990s and 2000s. A series of causative genes including *SLC5A1* (*SGLT1*) for glucose-galactose malabsorption (Martín et al. 1996), and *SLC26A3* (*DRA*) for congenital chloride diarrhea (Höglund et al. 1996) were the first to be identified, followed later by *MYO5B* for microvillus inclusion disease (Müller et al. 2008) and *EPCAM* for congenital tufting enteropathy (Sivagnanam et al. 2008). The availability of accessible and affordable targeted genetic testing led to a steady stream of causative genes being identified which significantly accelerated with the advent of next-generation sequencing technology and whole-exome sequencing over the last decade (Fig. 1). In addition to advances in genomics, improvements in clinical care of CoDE patients have led to a general increase in overall survival rates with a consequent rise in diagnostic efficacy. Advances in parenteral nutrition management, central line care, and the establishment of specialized centers and clinics have now transformed life expectancy in severe CoDE disorders from infancy to late childhood and beyond.

*Monogenic intestinal disorders and other monogenic causes of intestinal disease:* Monogenic intestinal epithelial disorders or CoDEs are defined by mutations that fundamentally alter epithelial cell function and subsequently result in intestinal disease. For most disorders, diarrhea is the predominant symptom with minimal or no inflammatory component present in the intestinal mucosa. However, loss of epithelial barrier function or disruption of normal mucosal architecture, for example in *TTC7A* deficiency (Dannheim et al. 2022) or alteration of normal host–microbiome responses (e.g., *SLC9A3* deficiency (Kini et al. 2022)) can result in a secondary mucosal inflammatory response. This contrasts and can be distinguished from monogenic causes of very-early-onset inflammatory bowel disease such as *IL10* mutations or other primary immune deficiencies with intestinal involvement such *WAS* mutations (Wiskott-Aldrich) or *FOXP3* mutations (IPEX) that primarily affect immune cell function, or development (Nambu and Muise 2020). As such, hematopoietic stem cell transplant (HSCT) is, therefore, often curative for very-early-onset inflammatory bowel disease, whereas this is not the case for CoDE disorders. Another distinguishing feature of CoDE disorders is primary involvement of the small intestine with variable involvement of the colon, resulting in a generalized enteropathy with consequent nutrient malabsorption and loss of normal fluid and electrolyte handling.

*Evaluation of CoDE disorders:* The diagnostic evaluation of CoDE disorders has evolved considerably over the past few years with the advent of accessible genetic testing and gradual increased recognition of these disorders by pediatric medical practitioners. General diagnostic approaches have emerged over the past few years to aid in the initial evaluation for infants with suspected CoDEs (Thiagarajah et al. 2018; Elkadri 2020; Younis et al. 2020; Russo 2020; Mantoo et al. 2021). In summary, most approaches focus on identification of infants with a history and symptoms suspicious for a CoDE disorder, initial dietary and stool evaluation, early genetic testing, and endoscopic biopsy evaluation as soon as safely possible.

## Challenges with CoDE disorders

Several significant challenges have historically faced the field of CoDE disorders. A significant issue, as with many monogenic disorders, is the rarity of individual CoDE disorders. This has led to a general lack of awareness in many medical facilities for diagnostic evaluation, a general paucity of data on the epidemiology, natural history, and management, and relatively little mechanistic information on the cellular pathophysiology for many disorders. For many patients, the lack of early recognition or suspicion for a CoDE disorder leads to a diagnostic odyssey, often characterized by unnecessary testing and interventions, and lengthy hospitalizations leading to increased mortality and morbidity. The nature of these disorders, with often profound loss of enteral absorptive function and severe growth failure and malnutrition during the most critical window for physical and cognitive development, means that early diagnosis and appropriate clinical management can have profound effects on patient prognosis and outcomes. In addition to the clinical effects on the patients themselves, this diagnostic odyssey places a large financial burden on the healthcare system and families. A recent comparison for a single CoDE patient in the U.S. (LeBlanc et al. 2020), showed an approximately $4 million difference in the hospital/clinical costs between before and after diagnosis.

The last decade has brought remarkable improvements in clinical nutrition care for CoDE patients, and the careful management of parenteral nutrition and indwelling central venous catheters have led to decreases in the mortality of even patients with severe CoDE disorders such as microvillus inclusion disease and tufting enteropathy. A major remaining challenge, however, is the lack of both effective symptomatic therapies for fluid loss and diarrhea and specific curative or disease-modifying therapies. Efforts to advance our understanding of the cell biology and physiology of intestinal epithelial function and the effect of CoDE disorder mutations on target proteins will be critical for development of future therapeutic options.

## Overview of the intestinal epithelium

The intestinal epithelium is organized as a single layer of cells that line the surface the intestinal tract. In the small intestine, this layer is architecturally characterized by glands (crypts) and finger-like projections (villi). Intestinal epithelial cells (IECs) are generated from pluripotent stem cells that reside in the base of the crypts and are marked by the receptor protein Lgr5. These stem cells give rise to progenitor cell populations that ultimately differentiate into a wide range of different epithelial subtypes including secretory cells such as goblet cells, and enteroendocrine cells, as well as columnar absorptive cells (Santos et al. 2018; Gehart and Clevers 2019).

IECs demonstrate cell polarization, with two distinct plasma membrane surfaces or domains: apical or lumen facing, and basolateral or body facing with epithelial tight junctions demarcating the boundary between these domains. The apical surface of the IEC is the site of nutrient absorption. It is rich in both active and passive transport channels (e.g., ENaC, NHE3, SGLT1) and digestive enzymes (e.g., sucrase-isomaltase, lactase, enterokinase) (Kiela and Ghishan 2016). The apical surface of IECs also contains numerous microscopic projections, microvilli, which serve to increase the apical surface area and, thus, the nutrient absorbing capacity of the cell. IECs contain an intricate and polarized intracellular trafficking machinery that serves to maintain the localization of membrane proteins. The lateral surfaces of IECs are responsible for maintaining junctions between cells that strengthen epithelial integrity and prevent paracellular movement of most solutes. Proteins responsible for maintaining adhesion include the epithelial cell adhesion molecule (EpCAM) and E-cadherin, while those that form tight junctions include the claudins, occludin, and zonula occludens-1 (ZO-1) (Choi et al. 2017). The basolateral surface of IECs is the site of nutrient release into circulation via passive and active transport channels (e.g., GLUT2, ferroportin, SLC7A7). This side is also the location of the sodium–potassium ATPase (Na/K ATPase), which provides the primary electrochemical driving force for the majority of nutrient and electrolyte transport. Lastly, the basolateral surface is the site at which IECs are anchored onto the underlying basement membrane by integrins.

## Classification of CoDE disorders

Although they are commonly linked and defined by altered intestinal epithelial function, CoDE disorders exhibit a wide variety of cell and tissue level pathophysiological mechanisms. Despite this heterogeneity some broad categories have emerged to classify specific genes. As the specific causative genes for many cases of non-acquired severe infantile-onset chronic diarrhea can now be established, it has become important to shift away from syndromic or clinically defined naming of disorders to more specific designation by affected gene (e.g., *SPINT2* deficiency vs syndromic congenital sodium diarrhea). This is particularly relevant for current and future studies that aim to correlate specific mutations with phenotype, prognosis, and treatment. As with any attempt to classify such a heterogenous group of disorders there are specific genes/disorders that do not fit well in any one category. In some cases, such as the recently discovered *WNT2B* deficiency, this has allowed expansion or development of new categories. As new causative genes (e.g., *PERCC1*, *UNC45A*, *AGR2*) and new information on disease mechanisms continue to emerge, these categories will need ongoing revision and refinement. Disease can also be classified by other methods such as protein ontology (e.g., functional annotation) or clinical outcome (e.g., parenteral nutrition dependence).

Broadly, monogenic epithelial disorders can be classified into five major categories that comprise core modules of epithelial function. These are listed below (see also Fig. 1) and detailed descriptions of several disorders in each category follow:

- Epithelial transport
- Epithelial enzymes and metabolism
- Epithelial structure, trafficking, and polarity
- Enteroendocrine function
- Epithelial stem cell function

## Epithelial transport

Perhaps the most archetypal of CoDE disorders are those that involve alterations in intestinal epithelial transport function. Several disorders affect the process electroneutral sodium absorption and parallel chloride/bicarbonate handling (*SLC26A3*) either directly via loss-of-function mutations in NHE3 or indirectly via gain-of-function mutations in the receptor GUCY2C.* GUCY2C* mutations also cause activation of chloride secretion via apical membrane chloride channels. Nutrient dependent sodium absorption is altered in glucose-galactose malabsorption via SGLT/SLC5A1 and loss of sodium-dependent bile transport in the ileum leads to congenital bile acid mediated diarrheas.

### Congenital chloride diarrhea and SLC26A3

The 21-exon solute carrier family 26 member 3 (*SLC26A3*) gene is mapped to chromosome 7q31 and encodes for the chloride/bicarbonate exchanger, also known as down regulated in adenoma (*DRA*), a transmembrane protein localized mainly to the apical side of the mucosa in the ileum and proximal colon (Byeon et al. 1996). Gamble et al. and Darrow likely described the first cases with *SLC26A3* mutations in 1945 in two patients who presented with watery diarrhea not responsive to conventional therapy and characterized by extremely high chloride content in the stools, in conjunction with low serum chloride and metabolic alkalosis (Darrow 1945; Gamble, et al. 1945). This syndrome was termed congenital chloride diarrhea (CLD). These observations were soon followed by several case reports with similar characteristics in the U.S, U.K, and France, as well as a large number of cases in Finland (Kelsey 1954; Perheentupa et al. 1965; Evanson and Stanbury 1965; Holmberg et al. 1977).

While *SLC26A3* mutations have been spotted around the globe, incidence and prevalence are uneven. Approximately 20% of the cases reported in Finland, where about 1:30,000–40,000 live births have the mutation overall, but where a higher prevalence is present in specific geographical areas (Wedenoja et al. 2010). The identification of p.Val317del in almost all Finish cases, the 3-base pair p.Ile675-676ins mutation in 47% of CLD-associated chromosomes in Polish cases, and the p.Gly187* mutation in 94% of chromosomes in affected Arab patients supports the existence of a founder effect in these populations (Höglund et al. 1998). A recent retrospective observational as well as prospective genetic analysis carried out in Japan, found that the unique c.2063-1G → T mutation was present in 7 out of 13 CLD patients and might be a founder mutation in East Asia (Konishi et al. 2019).

Our understanding of the genetics of *SLC26A3* mutations comes largely from a variety of in-depth studies in populations with a higher-than-normal incidence, such as in Finnish, Polish, and Arab populations, where there are specific autosomal recessive mutations in *SLC26A3* due to founder effects (Holmberg et al. 1977; Höglund et al. 1998; Wedenoja et al. 2011). Most mutations found so far are single-nucleotide substitutions, with missense, nonsense, insertions, and splice-site mutations identified. Although 110 mutations have been identified according to the Human Gene Mutation Database, with most described in recent years, genotype–phenotype associations remain incompletely understood. Further studies are needed to define associations and guide our understanding of how specific mutations affect SLC26A3 function.

The chloride/bicarbonate (HCO3^−^/Cl^−^) exchanger, encoded by *SLC26A3*, plays a major role in fluid homeostasis in the human body. It has the task of transporting chloride ions in exchange for bicarbonate across the apical membrane of intestinal epithelial cells. This drives electroneutral sodium absorption through coupled Na^+^/H^+^ exchangers (NHE3) in the intestine (Yu 2021). Therefore, in loss-of-function *SLC26A3* mutations, the inability to secrete HCO3^−^ into the intestinal lumen leads to acidic luminal bowel content, altering the driving forces and interfering with electroneutral sodium absorption with loss of normal fluid absorption and diarrhea. The loss of chloride absorption in parallel results in the characteristic high fecal chloride concentrations. Loss of normal body acid/base handling leads to the feature of a systemic metabolic alkalosis, unusual in most diarrheal disorders which generally lead to a systemic metabolic acidosis. Diagnosis of *SLC26A3* deficiency is strongly suggested by high corrected fecal chloride levels, greater than 90 mmol/L (Wedenoja et al. 2010). Diarrhea often starts in the intrauterine period, resulting in polyhydramnios and premature birth (Holmberg et al. 1975). Failure to thrive and nephropathy can also be seen, mimicking the presentation of Bartter syndrome with the notable difference that *SLC26A3* mutations do not lead to abnormalities in calcium levels. Given the similarities in the metabolic profile, it is, therefore, important to keep congenital chloride diarrhea as a differential diagnosis when evaluating an infant with suspected Bartter syndrome (Matsunoshita et al. 2018).

A number of reports (Asano et al. 2009; Shao et al. 2018) suggest that biallelic mutations in *SLC26A3* may confer increased risk for the development inflammatory bowel disease (IBD) later in life. This is supported by mouse studies of *Slc26a3* deficiency that show increased susceptibility to induction of colitis (Xiao et al. 2012) and consistent with data that show *DRA* expression is diminished in intestinal inflammatory states (Yu 2021). Current theories on the connection between *SLC26A3* and inflammation suggest that loss of transporter function may lead to a chronically altered luminal microbiome and mucosal environment leading to a loss of epithelial barrier function (Kini et al. 2022).

Treatment in congenital chloride diarrhea is primarily supportive with prevention of dehydration and electrolyte abnormalities through enteral fluid and electrolyte supplementation. In general, patients can avoid long-term parenteral nutrition if intensive management is instituted early, and most patients have a good long-term prognosis. Reduction of gastric chloride secretion by proton pump inhibitors has been proposed as a treatment (Aichbichler et al. 1997) although subsequent studies have found equivocal benefit (Höglund et al. 2001). The short-chain fatty acid butyrate, normally produced by colonic bacterial metabolism, has also been proposed as a potential therapy. Although not fully determined, the mechanism by which butyrate acts to improve diarrhea has been proposed to be via upregulation of chloride/butyrate transport, improved barrier function, or other trophic effects (Kelly et al. 2015; Deng et al. 2021). A number of studies have reported equivocal and genotype dependent results to butyrate administration in practice, although an alternative step-up approach for optimizing individualized doses has been recently proposed (Di Meglio et al. 2022).

### Congenital sodium diarrhea and SLC9A3

Congenital sodium diarrhea, characterized by neonatal onset severe watery diarrhea with an antenatal onset and high fecal sodium levels, was initially described in 1980s (Holmberg and Perheentupa 1985). Diarrhea with high fecal sodium levels has been identified in many cases associated with slightly different phenotypes and ultimately different causative genes. A syndromic condition with other abnormalities such as superficial punctate keratitis and choanal atresia was ultimately related to *SPINT2* mutations (Heinz-Erian et al. 2009). A non-syndromic form was found to be caused either by mutations in the *GUCY2C* gene or the Na^+^/H^+^ exchanger, *SLC9A3* (Fiskerstrand et al. 2012; Janecke et al. 2015). The role of *SLC9A3* in congenital sodium diarrhea was ultimately established with a cohort of nine patients from eight unrelated families who presented with sodium-rich watery secretory diarrhea, a history of maternal polyhydramnios, and abdominal distension. Seven patients were found by whole-exome sequencing, chromosomal microarray analysis or direct Sanger sequencing, to carry private homozygous or compound heterozygous *SLC9A3* mutations. In two patients only one exonic mutation was identified (Janecke et al. 2015).

To date, only approximately 12 patients have been described as having confirmed congenital sodium diarrhea secondary to *SLC9A3* mutations. A number of patients with a phenotype consistent with *SLC9A3*-mediated congenital sodium diarrhea have not been found to have coding mutation by standard whole-exome sequencing. The finding of single putatively pathogenic *SLC9A3* mutations in several cohorts raises the question of the existence of deep intronic or promoter-region variants in* SLC9A3* that remain to be discovered. The gradual adoption of whole-genome sequencing as a primary diagnostic modality should provide further information on this possibility in the future.

*SLC9A3*, mapped to chromosome 5p15.3, encodes for the Na^+^/H^+^ exchanger 3 (NHE3), localized primarily on the apical side of intestinal and proximal renal tubular epithelial cells. NHE3 is responsible for the bulk of electroneutral Na^+^ luminal absorption in the small and large intestine in exchange for intracellular protons, as well as for most reabsorption of filtered Na^+^ in the proximal renal tubules (Nwia et al. 2022). Studies in mouse models including the recent generation of an inducible intestinal epithelial-cell-specific *Nhe3* knockout mouse model mimicking congenital sodium diarrhea has yielded valuable information regarding the important role of NHE3 on intestinal epithelial integrity and its participation in acid–base homeostasis (Xue et al. 2020).

A variety of *SLC9A3* missense, splicing, and truncation mutations have been identified, with missense mutations the most common and without a clear genotype–phenotype correlation (Dimitrov et al. 2019). The polymorphism rs11739663, which is located close to *SLC9A3,* is a single-nucleotide polymorphism (SNP) associated with ulcerative colitis, suggesting a potential explanation for the association between *SLC9A3* mutations and the development of inflammatory bowel disease later in life (Jostins et al. 2012). As with congenital chloride diarrhea, *SLC9A3* deficiency is inherited in an autosomal recessive fashion. Patients affected can display severe abdominal distension due to dilated fluid-filled loops of bowel that can be mistaken for intestinal pseudo-obstruction.

Management of *SLC9A3* deficiency is largely supportive with careful nutritional management, sodium supplementation and management of metabolic acidosis, with a goal of optimizing nutrition and prevention of dehydration to allow for normal growth to be achieved. There are little systematic data on patient prognosis but case reports generally indicate that severe fluid and sodium loss is usually short-lived and many patients appear to have normal growth after the first few years of life (Bogdanic et al. 2022).

### GUCY2C

**Fig. 2:**
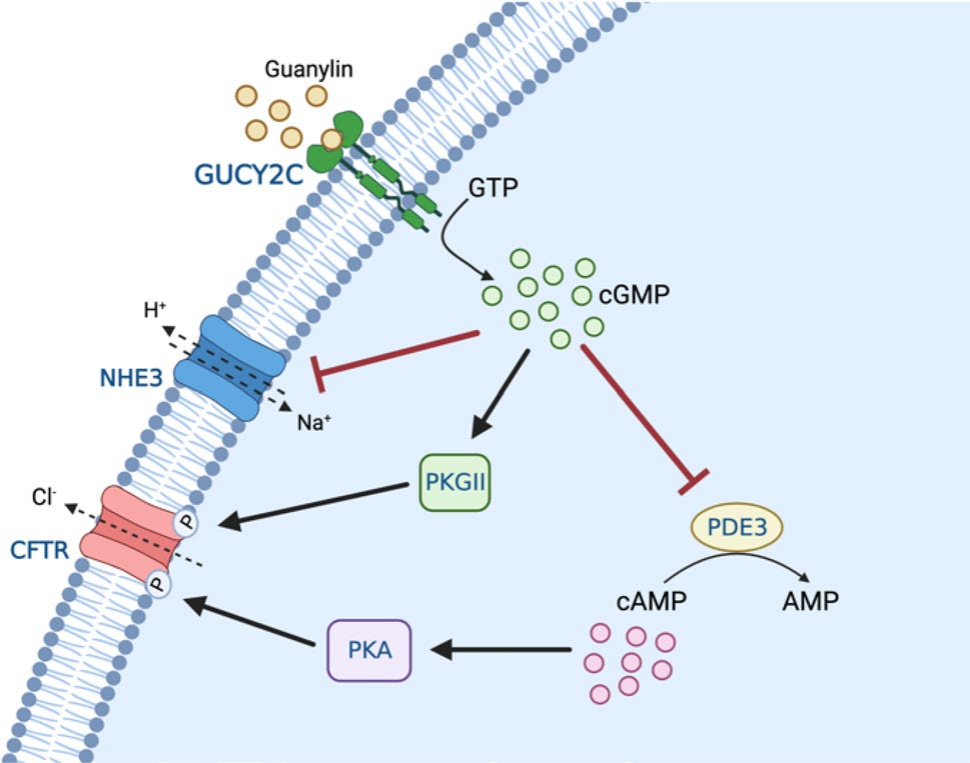
The effect of GUCY2C activation on epithelial electrolyte transport. GUCY2C (Guanylate cyclase 2C), cGMP (cyclic guanosine monophosphate), cAMP (cyclic adenosine monophosphate), PDE3 (phosphodiesterase 3), PKGII (Protein kinase G type 2), PKA (Protein kinase A), NHE3 (Sodium–hydrogen exchanger 3, SLC9A3), CFTR (Cystic Fibrosis Conductance Regulator)

*GUCY2C*, mapped to chromosome 12p12.3, encodes the homodimeric receptor enzyme Guanylyl cyclase C (GC-C), localized to the apical membrane of enterocytes and activated by guanylin, uroguanylin, and the heat-stable Escherichia coli enterotoxin heat-stable enterotoxin (STa). Activating mutations, which are inherited in an autosomal dominant manner, are a recognized cause of non-syndromic secretory congenital sodium diarrhea (Fiskerstrand et al. 2012). Activation by endogenous guanylin and uroguanylin, or from heat-stable enterotoxins, results in production of cyclic guanosine monophosphate (cGMP) and activation of downstream protein kinase-mediated signaling cascades. cGMP activates cGMP-dependent protein kinase II (PKGII) and inhibits the cyclic adenosine monophosphate (cAMP) phosphodiesterase PDE3 leading to indirect activation of cAMP-dependent protein kinase A (PKA) (Forte et al. 2000). Both protein kinase II and protein kinase A phosphorylate the cystic fibrosis transmembrane conductance regulator (CFTR) channel, resulting in elevated chloride secretion. In parallel, elevated cGMP levels inhibit electroneutral sodium Na^+^/H^+^ exchanger (NHE3), reducing sodium absorption and together with elevated chloride secretion result in fluid loss and diarrhea (Fig. 2).

Most patients with identified mutations in the *GUCY2C* gene present with early-onset watery secretory diarrhea, which is generally more severe in infancy and decreases with older age, possibly related to a greater receptor density in early childhood (Cohen et al. 1988). As with other causes of congenital diarrheas, potential complications include dehydration and metabolic acidosis. There is evidence for an association between these mutations and the development of irritable bowel syndrome, inflammatory bowel disease, small-bowel obstruction, and esophagitis (Fiskerstrand et al. 2012). More recently described cases report an antenatal presentation of secretory diarrhea, causing polyhydramnios during pregnancy and increasing the risk for complications such as sepsis, ileus, volvulus, and early-onset IBD (Müller et al. 2016). Further studies are needed to clarify how *GUCY2C* mutations are involved in the longer-term development of inflammatory bowel disease, small-bowel obstruction, and esophagitis.

Autosomal dominant mutations in* GUCY2C* were identified as a cause of congenital diarrhea through the analysis of a Norwegian family in which several members presented with chronic, mild early-onset diarrhea. Sequence analysis proved that the GUCY2C c.2519G → T missense mutation in exon 22 of the gene was present in all affected patients, causing the substitution of serine for isoleucine in codon 840, in the catalytic domain of the protein (Fiskerstrand et al. 2012). More recently, four distinct de novo missense mutations in the catalytic domain, in the linker region, and in the kinase homology domain of *GUCY2C* were identified, causing ligand-mediated activation of the receptor, and significantly elevated levels of basal intracellular cGMP (Müller et al. 2016). As with *SLC9A3* mutations, management of patients with *GUCY2C* mutations consists mainly of intensive nutritional and fluid support and sodium supplementation. New small molecule inhibitors of GC-C activity have recently been proposed, showing encouraging results in patient-derived enteroids (van Vugt et al. 2021).

### SLC5A1

Glucose transporters are a varied group of membrane proteins that have the task of facilitating the transport of glucose across the plasma membrane. In general terms, these glucose transporters are divided into two families: the facilitated-diffusion glucose transporters (GLUT) and the sodium-dependent glucose transporters (SGLT) (Scheepers et al. 2004). The *SLC5A1* gene, specifically, mapped to chromosome 22q12.3, encodes the sodium-dependent glucose/galactose cotransporter 1 (SGLT1), a 73 k-Da transmembrane protein, which is present mainly in the small intestine, the heart, and the kidneys (Wright et al. 2011). It is the primary transporter mediating glucose absorption in the small intestine, and is important for normal glucose, sodium and fluid absorption in the proximal small intestine.

Loss-of-function mutations in *SLC5A1* as a cause for glucose-galactose malabsorption were first discovered in the 1990s by Martin et al. (Martín et al. 1996). Reported cases of glucose-galactose malabsorption resulting from *SLC5A1* mutations remain rare, with only approximately 300 cases reported in the literature. SGLT1 is a high-affinity, low-capacity transporter located primarily on the luminal side of mucosal intestinal cells and in the renal proximal tubule. Its function is to transport one molecule of glucose along with two sodium molecules into the cell, taking advantage of the electrochemical gradient established by the Na^+^/K^+^ ATPase pump. It plays a critical role in the absorption of d-glucose and d-galactose across the apical brush-border membrane of enterocytes. Glucose then reaches the circulation through the GLUT2 transporter located in the basolateral membrane (Scheepers et al. 2004; Wright et al. 2011).

As a member of the SGLT family, SGLT1 is a 664-amino acid protein that contains 14 alpha-helical transmembrane domains. Its hydrophobic cytoplasmic C-terminal domain contains the five terminal transmembrane helices involved in glucose-binding and translocation (Wright et al. 2011). The N-terminus of wild-type SGLT1 has proven to be extracellular. Missense, nonsense, frameshift, and splice-site mutations cause the SGLT1 protein to lose its function, and studies of missense mutations have provided critical information regarding the structure and transport mechanism of the protein (Wright et al. 2002). Loss of SGLT1 function results in glucose-galactose malabsorption (GGM), an autosomal recessive inherited disorder that manifests within the first weeks of life because of the inability to absorb and utilize the monosaccharides glucose and galactose. Children with GGM present with a diet-induced diarrhea that begins when they are fed breastmilk or formula. This diarrhea can cause severe dehydration and metabolic acidosis, making early diagnosis and management critical. An initial diagnosis can be established clinically, by dietary reversal of symptoms with glucose/galactose-free formula substitution but should be followed by confirmatory genetic testing. Management of GGM consists of instituting a strict glucose/galactose-free diet, limiting carbohydrate intake to fructose initially but addition of small amounts of glucose in the diet can occur as patients grow older (Chan et al. 2021). Once past infancy, patients can generally manage diarrhea with diet alone and generally do well, although there are no systematic studies of long-term health outcomes in GGM patients.

## Enzymes and metabolism

A group of CODE disorders are caused by mutations in a heterogenous collection of genes involved in the metabolic processing of nutrients in epithelial cells. These include several intestinal brush-border enzymes involved in carbohydrate uptake such as sucrose-isomaltase (SI) and key proteins in intestinal fat processing such as diacylglycerol O-acyltransferase 1 (DGAT1). A very novel member of this group is *AGR2* which is involved in normal protein folding and the secretion of mucin proteins from epithelial goblet cells.

### Sucrase-isomaltase deficiency and SI

The sucrase-isomaltase (*SI*) gene, mapped to chromosome 3q26.1, codes for sucrase-isomaltase, a heterodimeric protein with two subunits, sucrase and isomaltase. Sucrase-isomaltase is a type II transmembrane disaccharidase glycoprotein expressed in the intestinal brush border (Naim et al. 1988). Initially, the protein encoded by the *SI* gene is a precursor protein that is later cleaved by pancreatic proteases into sucrase and isomaltase. While sucrase hydrolyzes sucrose, isomaltase processes starch, isomaltose, and maltose. Therefore, patients with biallelic mutations of the *SI* gene are unable to metabolize these carbohydrates, and their consumption leads to a diet-induced diarrhea accompanied by different degrees of abdominal bloating and pain. This condition, congenital sucrase-isomaltase deficiency (CSID), was initially identified and described in the 1960s (Weijers et al. 1960).

There is still no clear consensus on the worldwide prevalence of congenital sucrase-isomaltase deficiency, due to the nonspecific nature of symptoms and variation in disease severity. Estimates place the mutation at around 0.2% in individuals of European descent, 5–10% in Greenland Innuits, and 3–7% in Canadian and Alaskan Innuits (Treem 2012). Four mutations are estimated to represent over 80% of CSID in European populations: p.Gly1073Asp, p.Val577Gly, p.Phe1745Cys, and p.Arg1124* (Gericke et al. 2017). A molecular and cellular analysis of 13 missense mutations has allowed for their classification into three major phenotypes based on whether protein trafficking, enzymatic activity, or lipid raft association is affected (Gericke et al. 2017). In general terms, however, these mutations result in some degree of improper targeting to the plasma membrane and a combined deficiency of both sucrase and isomaltase (Naim et al. 1988).

Diagnosis of congenital *SI* deficiency is made definitively with a duodenal or jejunal biopsy and a disaccharidase assay (Puntis and Zamvar 2015). Patients are advised to adhere to a sucrose and starch-free diet, which only partially relieves symptoms. If necessary, enzyme replacement therapy with sacrosidase may be instituted, allowing for a return to close-to-normal intestinal activity.

### DGAT1 deficiency

**Fig. 3:**
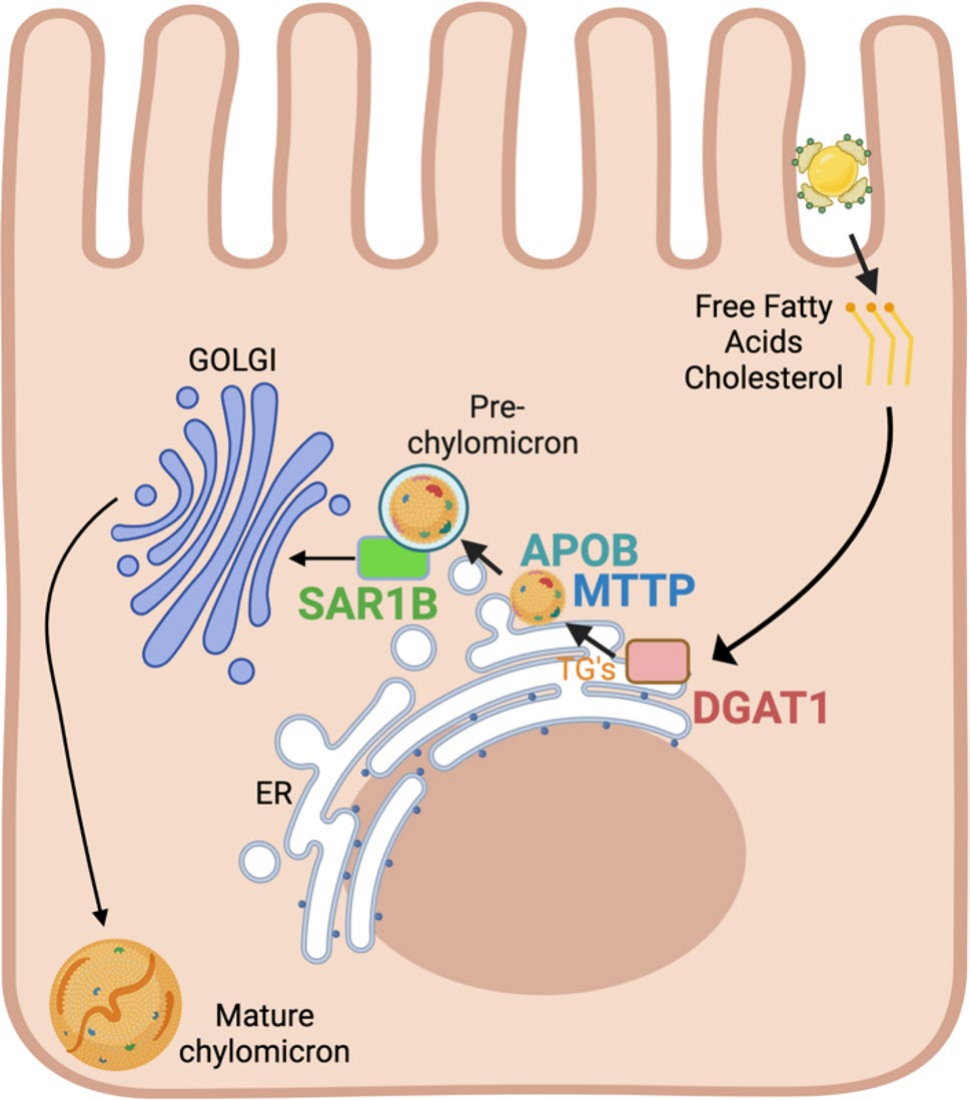
CODE genes/proteins involved in intestinal epithelial fat processing. DGAT 1 (Acyl-CoA diacylglycerol acyltransferase), MTTP (microsomal triglyceride transfer protein), APOB (apolipoprotein B), SAR1B (secretion-associated Ras-related GTPase 1B)

Diacylglycerol O-acyltransferase 1 (DGAT1) is a microsomal enzyme that plays a critical role in synthesizing cellular triacylglycerols (Fig. 3), using diacylglycerol and fatty acyl CoA as substrates (Wang et al. 2020a). The human *DGAT1* gene has been mapped to chromosome 8q through fluorescence in situ hybridization (Cases et al. 1998). Biallelic splice-altering loss-of-function mutations in *DGAT1* were first described as causing autosomal recessive disease in an Ashkenazi Jewish family in 2012 (Haas et al. 2012). The clinical picture of these patients was characterized by severe electrolyte-transport-related diarrhea (ETRD), vomiting, growth failure, protein-losing enteropathy, and hypoalbuminemia. Additionally, these patients presented with elevated fasting triglycerides and cholesterol levels (Haas et al. 2012; Gluchowski et al. 2017).

Biopsy of the small intestine in patients with *DGAT1* deficiency reveals mild villous blunting and chronic inflammation with eosinophilia in the lamina propria (Xu et al. 2020; Eldredge et al. 2021). Some authors have reported lipid droplets visible on the surface of epithelial cells without lymphatic dilation (Eldredge et al. 2021).

The structure of DGAT1 was recently solved by two separate groups in 2020 (Sui et al. 2020; Wang et al. 2020a). In its mature form, DGAT1 is present as a dimer or a tetramer on the plasma membrane. It is composed of nine transmembrane domains with one luminal loop and two extracellular loops. The catalytic histidine residue, His415, is attached to the 7th transmembrane domain, which forms a tunnel-like channel with the 8th transmembrane domain to create the active site.

Disease-associated mutations identified in *DGAT1* are mostly made up of nonsense, deletion or duplication-induced frameshift, and splice-altering mutations. Most of these variants likely represent effective knockout alleles whose products are destroyed by nonsense-mediated decay. Two missense mutations (p.Leu105Pro and p.Leu295Pro) and one in-frame deletion (p.Ala226_Arg250del) have been identified (Stephen et al. 2016; Eldredge et al. 2021). The p.Leu105Pro mutation has been proven to cause only partial loss of DGAT1 function, and, as a result, a less severe clinical picture (Gluchowski et al. 2017). The exact mechanisms by which loss of DGAT1 function in intestinal epithelial cells leads to diarrhea and protein-losing enteropathy remain poorly understood. Recent studies point to a possible mechanism involving increased lipid-induced ER stress and susceptibility to lipid-induced cell death (van Rijn et al. 2018, 2019). Treatment of these patients entails correction of fluid status and underlying electrolyte abnormalities along with implementation of a fat-restricted diet (Gluchowski et al. 2017).

### Abetalipoproteinemia and homozygous hypobetalipoproteinemia: MTTP and APOB

Abetalipoproteinemia (ABL) and homozygous hypobetalipoproteinemia (HHBL) are syndromes resulting from the total absence or extremely low levels of apolipoprotein B. These patients present similarly during infancy with fatty diarrhea and associated fat-soluble vitamin malabsorption (Leppert et al. 1988; Zamel et al. 2008). Later in life they may develop the stigmata of fat-soluble vitamin deficiencies, in particular ataxia, peripheral neuropathy, and atypical retinitis pigmentosa (Zamel et al. 2008; Welty 2014). Patients with ABL and HHBL are clinically indistinguishable from one another except by the phenotype of their parents: heterozygous parents of patients with ABL will have normal lipid panels while the parents of patients with HHBL will have half-normal levels of ApoB-containing lipoproteins (Lee and Hegele 2014).

Ultimately these syndromes are defined biochemically by low or undetectable levels of circulating chylomicrons, VLDL, and LDL (Leppert et al. 1988; Welty 2014). The appearance of ABL and HHBL on EGD and histology is very similar to that of chylomicron retention disease. These patients have a “white hoar frosting” appearance to the mucosa, which on biopsy reveals clarified lipid-laden enterocytes that stain with oil red O (Lee and Hegele 2014). Additionally, these patients display baseline acanthocytosis on peripheral blood smear (Zamel et al. 2008).

Apolipoprotein B is encoded by the *APOB* gene and exists in two major isoforms, apoB-100 and apoB-48 (Welty 2014). The full-length apoB-100 isoform is used by the liver to package and secrete triglyceride-loaded VLDL, while the splice variant apoB-48 is used by enterocytes to package and secrete triglycerides and cholesterol in chylomicrons (Fig. 3). The *MTTP* gene encodes the mitochondrial triglyceride transfer protein (MTP), a protein which heterodimerizes with protein disulfide isomerase (PDI) and loads lipids onto apolipoprotein B (Fig. 3) to form mature VLDL and chylomicrons (Zamel et al. 2008). Homozygous mutations in *MTTP* cause ABL, while homozygous mutations in *APOB* cause HHBL (Lee and Hegele 2014). Of note, patients heterozygous for *APOB* mutations display co-dominance resulting in half-normal LDL, VLDL, and chylomicrons. Patients with certain polymorphisms of these genes present only with modified risk of metabolic syndrome, hyperlipidemia, and hepatic steatosis (Hsiao et al. 2015).

Apolipoprotein B is a particularly large protein at a length of 4536 amino acids in its full-length form (apoB-100) and 2152 amino acids in its splice variant form (apoB-48) (Hooper et al. 2005). It is composed of an N-terminal signal sequence (which is co-translationally cleaved) followed by a βα1 domain which houses the MTP and lipoprotein lipase binding sites, a C-terminal LDL receptor binding site (residues 3359 to 3369), and numerous beta strands and alpha helices throughout to form a belt-like structure (Hooper et al. 2005; Benn et al. 2008). Disease-associated mutations in *APOB* are largely composed of truncations, splice variants, and nonsense mutations. Only one missense mutation has been identified, p.Arg463Trp, which was originally reported as “p.Arg490Trp” when first described in 2003 (Hooper et al. 2005; Ayoub et al. 2021). This mutation is found primarily in individuals of Lebanese descent (Ayoub et al. 2021). The exact mechanism by which it induces HHBL remains unclear.

MTP is structurally composed of an N-terminal signal peptide, a beta-barrel domain, an alpha-helical domain, a lipid binding domain, and a C-terminal KDEL sequence (Biterova et al. 2019). Disease-associated mutations identified in MTP are highly diverse, including numerous insertion and deletion-induced frameshifts, splice mutations, nonsense mutations, loss-of-start mutations, and missense mutations. While disease-associated missense mutations are found in each of the three major domains, most are found in the C-terminal stretch of the alpha-helical domain from residues 528 to 590: p.Tyr528His, p.His529Arg, p.Arg540Cys, p.Arg540His, p.Pro552Leu, p.Ile564Thr, p.Ser590Ile (Vlasschaert et al. 2021). The Arg540 residue sits at the N-terminal portion of alpha helix 14 in the alpha-helical domain. Mutations replacing the arginine at this site have been shown to result in preserved apoB and PDI binding, but exhibit substantially impaired lipid-transfer activity (Miller et al. 2014).

The steatorrhea caused by ABL and HHBL is responsive to a reduced fat diet (< 30% of total calories) with supplementation of essential fatty acids and fat-soluble vitamins (Welty 2014). Evaluation of the extent of liver disease in these patients is key, as some may develop end-stage liver disease at an early age and require liver transplantation (Burnett et al. 2021).

### Chylomicron retention disease and SAR1B

Mutations of the *SAR1B* gene, which encodes for the Sar1b protein, are mapped to chromosome 5q31.1 and are implicated in the development of chylomicron retention disease (CRD), now presumed to be the same disorder as Anderson disease. *SAR1B* deficiency is characterized by severe fat malabsorption, failure to thrive, diarrhea, and vomiting in infancy or childhood. Consequently, patients affected by a variety of loss-of-function variants of the *SAR1B* gene present with low blood cholesterol levels, absent chylomicrons, deficiency of cholesterol-dependent fat-soluble vitamins, and subsequent neurological impairment.

The presentation of CRD on esophagogastroduodenoscopy (EGD) is quite striking, with authors describing white duodenal and jejunal mucosa (Charcosset et al. 2008; Ferreira et al. 2018). This correlates with fat-laden enterocytes on histology and numerous lipid droplets appreciable on electron microscopy (Georges et al. 2011; Ferreira et al. 2018). These lipid droplets may be highlighted with oil red O stain on histologic preparation.

SAR1B is a cellular GTPase which, in intestinal epithelial cells, is critical for the formation and transport of chylomicrons from the endoplasmic reticulum to the cis-Golgi (Fig. 3). This is a vital part of the process of fat transport and absorption via cellular transcytosis. Its specific function is to recruit COPII complex heterodimers to the ER membrane in a GTP-dependent manner (Suda et al. 2017; Auclair et al. 2021). This function is redundant between SAR1A and SAR1B in most tissues; however, the formation of pre-chylomicrons appears to be entirely SAR1B-dependent (Peotter et al. 2019). Both SAR1 proteins are composed of an N-terminal amphipathic helical domain followed by two switch domains, switch I and switch II, which enable the SAR1 proteins to change conformation during GTP hydrolysis. Residues 32–39 contain a GxxxxGKT Walker A motif and is required for both Sec12 docking and GTP loading (Peotter et al. 2019).

Disease-associated mutations identified in *SAR1B* include frameshifts, nonsense mutations, in-frame deletions, a loss-of-start mutation, and several missense mutations. The in-frame deletion p.Ser117_Lys160del eliminates a long portion of the protein, including an important binding site for GTP (Jones et al. 2003). The p.Glu114del mutation produces CRD by removing a single glutamic acid residue, but the mechanism of this mutation’s pathology remains uncertain (Doya et al. 2021). Missense mutations which have been identified include p.Gly37Arg, which removes a key glycine in the GxxxxGKT motif, and p.Gly185Val, which removes a structurally important glycine at the beginning of an alpha helix (Jones et al. 2003; Peotter et al. 2019). The additional missense mutations p.Asp137Asn and p.Ser179Arg involve residues which help form the active site and interact with GTP through hydrogen bonding. The p.Asp137Asn mutation is a particularly common variant which has been found in multiple French-Canadian families (Jones et al. 2003; Charcosset et al. 2008). Management of chylomicron retention disease generally consists of a diet low in long-chain fatty acids and supplementation of fat-soluble vitamins, particularly vitamins A and E.

### Enterokinase deficiency and TMPRSS15

The *TMPRSS15* gene encodes the enzyme enterokinase (also called enteropeptidase), a key player in the pancreatic enzyme activation cascade. The link between malabsorption and enterokinase deficiency (EKD) was first established by Hadorn et al. in 1969, who identified an infant with chronic malabsorption and low proteolytic enzyme activity which could be corrected by the addition of exogenous enterokinase in vitro (Hadorn et al. 1969). This biochemical syndrome was not connected to a gene until 2002, when mutations in what is now known as the* TMPRSS15* or *ENTK* gene were identified in three patients (Holzinger et al. 2002). The clinical syndrome of these patients is characterized by failure to thrive, chronic diarrhea, low serum protein, and diffuse edema starting in the first weeks of life (Haworth et al. 1971; Holzinger et al. 2002; Madhusudan et al. 2021). While this disorder has been well characterized as a classic example of intestinal physiology and biochemistry, it remains extremely rare, with only about 20 cases reported since its discovery over 50 years ago (Madhusudan et al. 2021).

Enterokinase is a transmembrane serine peptidase expressed on enterocytes which serves the important role of activating trypsinogen to trypsin upon secretion of pancreatic juices into the duodenal space (Mössner 2010). Physiologically, this serves as one of many mechanisms which prevent premature activation of pancreatic enzymes prior to their arrival in the duodenum. Histopathology in patients with EKD is generally unremarkable, as is ultrastructural analysis (Hadorn et al. 1969; Haworth et al. 1971). However, enzymatic analysis of duodenal juices from these patients demonstrates low peptidase and enterokinase activity with preserved lipase and amylase activity (Hadorn et al. 1969).

Given the rarity of this disease and the fact that many cases were identified biochemically before the advent of readily available genetic testing, few disease-causing mutations have been identified in *TMPRSS15*. Most of the known pathogenic variants are nonsense mutations or frameshifts resulting in early truncation (Wang et al. 2020b; Madhusudan et al. 2021). Splice variants have also been reported, including the intronic c.1428 + 2T → G and the exonic p.Glu641Lys, the latter of which results in the skipping of exon 16 and produces an in-frame 47 amino acid deletion in the second LDL receptor-like domain. Lastly, the missense mutations p.Val799Asp and p.Gly1002Arg have been also reported. p.Val799Asp has been shown to reduce both expression and enzyme activity (Wang et al. 2020b). Both of these missense mutations localize to the C-terminal serine protease domain, suggesting that they may affect the folding or catalysis of this domain directly.

In terms of management, *TMPRSS15* deficiency can today be treated by replacement of exogenous pancreatic enzymes, similar to other disorders of pancreatic exocrine function. However, since EKD is so rare, there is no systematic data to help guide therapy and management guidelines are based on general treatment of pancreatic insufficiency.

### Eagles syndrome and AGR2

Recently, Al-Shaibi et al. reported the case of two siblings with congenital enteropathy and reduced goblet cells and mucin on intestinal biopsy (Al-Shaibi et al. 2021). These patients were found to have causative homozygous mutations in the gene *AGR2*; thus, their clinical syndrome was named “Enteropathy caused by *AGR2* deficiency, Goblet cell loss, and ER stress”, or EAGLES syndrome. Bertoli-Avella et al. subsequently described a case series of 13 patients from 9 families and identified new causative variants in *AGR2* (Bertoli-Avella et al. 2021). Of the patients described in this study, 6 of the 13 from three separate families share an identical 8.2 Mb on chromosome 7 and are of Syrian descent. Additional clinical features observed in these patients include recurrent lower respiratory tract infections and bronchiectasis, cardiac anomalies, and hepatosplenomegaly; however, there was no laboratory evidence of immunologic dysfunction in either report.

Duodenal biopsy from the two patients described by Al-Shaibi et al. demonstrated little to no inflammation, but a marked reduction in the number of normal goblet cells identifiable on hematoxylin and eosin stain along with apoptosis and regenerative crypts with mitotic figures. Staining for goblet cell marker TFF3 revealed an increased number of goblet cells relative to a non-inflamed control, but less goblet cells compared to an inflamed control. Gastric biopsy, on the other hand, demonstrated extensive intestinal metaplasia with a paucity of foveolar cells along with a lymphocytic infiltrate (Al-Shaibi et al. 2021). At present, this is the only description of histology from *AGR2*-deficient patients in the literature, as Bertoli-Avella et al. did not comment on the histology from patients in their cohort. Since no clear duodenal inflammation was identified in this case, we conclude that there is insufficient information to classify this enteropathy as inflammatory at this time. The gastric phenotype, on the other hand, is likely chemical gastritis due to the loss of a protective alkaline mucus barrier.

AGR2 is a protein disulfide isomerase (PDI) which helps regulate protein folding and disulfide bridging in the endoplasmic reticulum and may also be secreted to interact with the extracellular matrix independent of its PDI activity (Fessart et al. 2016; Jach et al. 2021). AGR2 is required for production of multiple gel-form mucins, including MUC2, via formation of mixed disulfide bonds in the precursor protein (Park et al. 2009; Jach et al. 2021). Structurally, AGR2 is composed of an N-terminal signal peptide, an adjacent unstructured N-terminal domain which facilitates cell–cell adhesion, a central thioredoxin-like domain, a central peptide-binding loop, and a C-terminal KTEL ER-retention sequence.

Across the 15 cases of *AGR2*-deficiency describe thus far, there is a mixture of missense variants and splice-altering variants, with one large deletion. All the identified variants have displayed an autosomal recessive pattern of disease inheritance. The missense variants identified include p.Pro71Thr, p.His117Tyr, and p.Gly143Glu. Interestingly, none of these missense mutations fall within the identified functional domains in AGR2 (Moidu et al. 2020). The p.Pro71Thr mutation falls between the N-terminal unstructured domain and thioredoxin-like domain, and may result in a loss of proper orientation of the thioredoxin-like domain. The p.His117Tyr mutation falls between the thioredoxin-like domain and peptide-binding domain, which may alter the orientation of either of these domains or act by another mechanism. The p.Gly143Glu mutation falls shortly after the peptide-binding domain, and may alter result in interruption of a nearby alpha helix by the bulky glutamine side chain (Bertoli-Avella et al. 2021). The identified splice-altering mutations in *AGR2*, c.330 + 1G → T and c.330 + 1del, both involve the loss of normal exon 5 splicing. The large deletion variant described in the literature results in the loss of exon 1 through exon 7 of *AGR2*, as well as the entirety of the neighboring gene *AGR3*. This deletion is the only definitive complete loss of function of *AGR3* identified, and was found in a homozygous state, demonstrating that *AGR3* is not required for life.

Given that this disease has only very recently been described, there is little published information regarding treatment of these patients. Many of these patients will likely require at least partial parenteral nutrition. Their clinical similarity to patients with cystic fibrosis has been noted in the literature, with *Pseudomonas aeruginosa* colonization and bronchiectasis at an early age (Bertoli-Avella et al. 2021). Screening for pulmonary disease is, thus, an important clinical consideration in the care of these patients.

### Congenital lactase deficiency and LCT

The enzyme lactase-phlorizin hydrolase (LPH), key to our ability to digest lactose in milk, is a brush-border β-galactosidase encoded by the *LCT* gene located on chromosome 2q21 (Anguita-Ruiz et al. 2020). While many humans rapidly lose LPH expression following the weaning period, infants display LPH expression irrespective of their future lactase persistence status. Congenital lactase deficiency (CLD) is an autosomal recessive disorder. Neonates with CLD who are fed with lactose-containing milks present with watery diarrhea during the first week of life (Järvelä et al. 1998). The diarrhea induced by lactose in the presence of CLD is a classic example of a diet-induced osmotic diarrhea. These cases may produce severe diarrhea with life-threatening dehydration and metabolic acidosis (Wanes et al. 2019). While the clinical and biochemical syndrome of CLD had been described as early as the 1960s, the *LCT* gene was not discovered until 1998 by Jarvela et al. Cases of congenital lactase deficiency have been identified across many ethnicities; however, the majority of mutations identified have been in individuals of Finnish ancestry (Järvelä et al. 1998; Diekmann et al. 2015; Wanes et al. 2019).

Small intestinal biopsy of patients with CLD demonstrates normal histology without villus atrophy or inflammation, with low or absent lactase activity (Torniainen et al. 2009). In addition to lactose, LPH also catalyzes the hydrolysis of cellobiose, cellotriose, lactosylceramide, flavonoid glucosides, and phlorizin (Anguita-Ruiz et al. 2020). It is composed of an N-terminal signal peptide (Met1 to Gly19) followed by four homologous domains (Troelsen 2005; Diekmann et al. 2015). Domains I (Gly19–Arg734) and II (Leu 735–Arg868) are vital for proper LPH trafficking to its site of biologic activity. Domain III contains the phlorizin hydrolase active site while Domain IV contains the lactase active site (Jacob et al. 2002; Diekmann et al. 2015). The protein is anchored to the membrane by a C-terminal single pass transmembrane hydrophobic domain. Domains I and II are cleaved off in the process of translocation and the final brush-border membrane-bound form of LPH begins at contains only Domains III and IV (Wanes et al. 2019).

Mutations that cause CLD are a mixture of nonsense mutations, duplication and deletion-induced frameshift mutations, and missense mutations (Wanes et al. 2019). Frameshift and nonsense mutations are distributed evenly throughout the protein. Similarly, missense mutations are found in both the cleaved trafficking domains (e.g., p.Gln268His, p.Ser688Pro) and the catalytic domains (e.g., p.Ser1121Leu, p.Gly1363Ser) of LPH (Torniainen et al. 2009; Sala Coromina et al. 2015; Wanes et al. 2019). The p.Tyr1390* variant represents a particularly common founder mutation within the Finnish population (Kuokkanen et al. 2006). While most variants have been identified in only one ethnicity, the mutation p.Gly1363Ser has been found in patients of Finnish, Turkish, and Persian descent. This p.Gly1363Ser displays a fascinating phenotype whereby a novel N-glycosylation site is created and the protein displays temperature-dependent ER transit. At physiologic temperatures, p.Gly1363Ser LPH becomes trapped within the ER. However at 20 degrees Celsius, p.Gly1363Ser LPH is able to fold properly and traffics normally to the cell membrane (Wanes et al. 2019). The exact mechanism by which the other missense mutations in *LCT* result in CLD remains unclear.

Once identified, CLD represents one of the most treatable forms of congenital enteropathy. Patients respond quickly to a lactose-free diet and lactase supplementation (Peretti and Mas 2022). The unique nature of the common p.Gly1363Ser variant has prompted some authors to question whether a chaperone-based therapy could aid in variant lactase folding and treat lactase deficiency in a subset of patients (Wanes et al. 2019).

## Epithelial structure, trafficking, and polarity

Genes involved in epithelial structure, trafficking and polarity comprise an important group of CoDE disorders, and include classical disorders such as Microvillus Inclusion Disease (*MYO5B*) and Tufting Enteropathy (*EPCAM*). Several genes involve polarized trafficking, which in epithelial cells relies on unique vesicular compartments not always present in other cells. The epithelial apical recycling pathway involving Myo5B powered vesicular trafficking has been found to be affected by mutations to multiple different genes (*MYO5B*, *STX3*, *UNC45A*, *STXBP2*), pointing to the importance of this pathway in normal epithelial function. Congenital disorders of glycosylation involving the intestinal epithelia are a well-known but poorly understood cause of intestinal dysfunction. Lastly, mutations in genes such as *TTC7A* and *SPINT2* cause interesting structural and developmental defects in epithelial cells. Gene loss-of-function studies in many of these disorders have revealed fascinating and deep insights in the fundamental cell biology of intestinal epithelial cells.

### Microvillus inclusion disease and MYO5B

Microvillus inclusion disease (MVID) was first described in a 1978 case series of five infants by Davidson et al. The infants presented with persistent diarrhea from birth and histopathologic findings of villous atrophy, crypt hyperplasia, and “lysosome-like inclusions” (Davidson et al. 1978). These patients typified the severe pathology and poor prognosis associated with the disease. Thirty years later, it was identified that variants of *MYO5B*, a gene encoding the unconventional myosin motor, myosin Vb, were associated with these findings (Müller et al. 2008). Now an established diagnosis, MVID affects approximately 1 in 1,000,000 live births and displays autosomal recessive inheritance (Ruemmele et al. 2006). MVID patients present with severe watery diarrhea within the first days of life, requiring long-term parenteral nutrition. A subset of patients demonstrates a later onset of disease starting at several months of age with better overall prognosis and a possibility of future enteral autonomy (Müller et al. 2008). There is an increased prevalence of MVID-associated *MYO5B* variants among certain ethnic populations, such as those of Navajo or Middle Eastern descent (Erickson et al. 2008). Interestingly, mutations in *MYO5B* have also been reported as causative for progressive familial intrahepatic cholestasis (PFIC), and the literature demonstrates a strong correlation between mutation characteristics and disease phenotype (Aldrian et al. 2021).

**Fig. 4:**
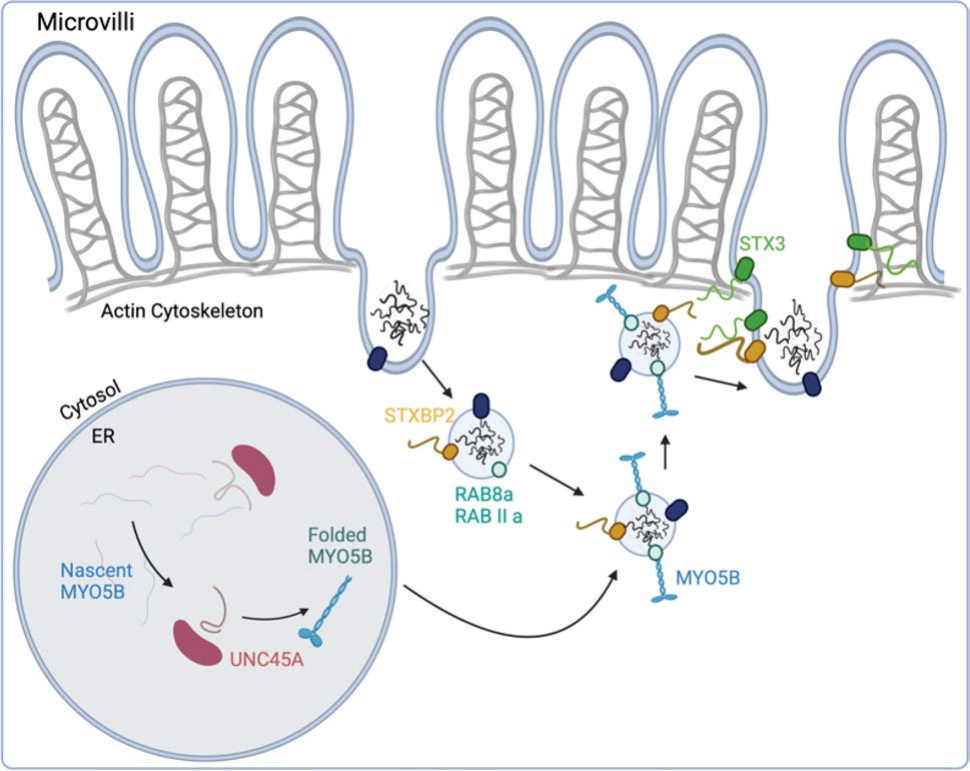
Myosin Vb-dependent epithelial vesicular trafficking. MYO5B (Myosin Vb), UNC45A (Unc45 Myosin Chaperone A), STXBP2 (syntaxin-binding protein 2), STX3 (Syntaxin 3), RAB8A (Ras-related protein RAB8a), RAB11A (Ras-related protein 11a)

MVID is fundamentally a disorder of endosomal trafficking characterized by defective apical membrane recycling. In the healthy intestinal epithelium, early endosomes sort cargo among three major pathways: the retrograde Golgi transport pathway, the degradation pathway, and the recycling pathway (O’Sullivan and Lindsay 2020). The apical recycling pathway in intestinal epithelial cells is responsible for delivering cargo back to the cell membrane. To do so, it relies on the motor activity of myosin Vb along the actin cytoskeleton. Rab8a and Rab11a serve as linkers between the recycling endosome and the C-terminal cargo-binding domain of myosin Vb (Fig. 4). Once the cargo has been delivered adjacent to the cell membrane, interaction between t-SNARE syntaxin-3 (STX3) and Sec/Munc-family protein syntaxin-binding protein-2 (STXBP2, also known as Munc18-2) is required for membrane fusion (Knowles et al. 2014; Vogel et al. 2015). Together, these proteins constitute a myosin Vb-STX3-STXBP2 axis required for proper apical recycling, and loss of any of these major components results in an intestinal MVID phenotype (Dhekne et al. 2018). While Rab8a and Rab11a are similarly vital for this pathway, there are no known MVID-associated variants in either of these proteins. Of note, knockdown of either Rab in mice results in an MVID phenotype with characteristic microvillous inclusions in electron microscopy (Sato et al. 2007; Sobajima et al. 2014). UNC45A, a chaperone which aids in myosin Vb folding, has also become recognized as a prerequisite for proper apical recycling (Duclaux-Loras et al. 2022). It is the combination of the loss of epithelial surface area, loss of epithelial transporters, loss of appropriate vesicular traffic, and leaky tight junctions that is ultimately responsible for malabsorption in these patients.

In keeping with the nature of defective recycling in MVID, small subapical endosomes containing apical membrane components such as villin, the Na^+^/H^+^ exchanger-3 (NHE3), and organized microvilli are the histopathologic hallmark of the disease (Ruemmele et al. 2006; Knowles et al. 2014). These inclusions may be highlighted by PAS stain or immunohistochemistry for apical membrane proteins and are best appreciated on electron microscopy. Histology is otherwise notable for villous atrophy without inflammation or crypt hyperplasia.

Structurally, myosin Vb is composed of an N-terminal motor domain, an intermediate elongated myosin V lever arm, and a C-terminal cargo-binding domain (Knowles et al. 2014; Dhekne et al. 2018). The range of reported MVID-inducing mutations in *MYO5B* includes missense mutations, nonsense mutations, splice-altering mutations, insertions, deletions, and duplications (van der Velde et al. 2013). An overwhelming predominance of the missense mutations affecting *MYO5B* localize to the myosin Vb motor domain. These disease-related missense mutations have been reported to interfere with F-actin binding (p.Cys514Arg, p.Leu528Phe, p.Arg531Trp, p.Phe538Ser, p.Ile550Phe, p.Pro619Leu), ATP binding (p.Gly168Arg), ATP hydrolysis (p.Arg219His), motor domain mechanics (p.Val108Gly, Gly316Arg, p.Arg401His, p.Asn456Ser, p.Arg656Cys), and protein folding (p.Ala143Glu, p.Gly435Arg, p.Pro660Leu) (van der Velde et al. 2013; Dhekne et al. 2018). In contrast to missense mutations, disease-associated truncating mutations are evenly distributed throughout the length of the protein (Aldrian et al. 2021).

Aldrian et al. assessed the differential association of *MYO5B* mutation characteristics with an MVID phenotype, a PFIC phenotype, or a mixed phenotype. This group demonstrated that total loss of myosin Vb from nonsense-mediated decay tends to result in an MVID phenotype without intrahepatic cholestasis (Aldrian et al. 2021). In contrast, late truncations (especially in compound heterozygosity with complete loss-of-function mutations) tend to result in a mixed phenotype of MVID with intrahepatic cholestasis. The behavior of missense mutations in *MYO5B* is more difficult to predict, with some missense mutations resulting in a pure MVID phenotype and some in a mixed MVID/PFIC phenotype (Qiu et al. 2017; Aldrian et al. 2021). A small subset of missense mutations, including p.Cys266Arg, and p.Ser158Phe, were found to cause intrahepatic cholestasis with displacement of bile canalicular transporters while preserving intestinal function. It remains unclear what features of these missense mutations predispose to their respective disease phenotypes.

Some MVID-associated mutations are found to be associated with less severe disease outcomes. The motor domain mutation p.Val108Gly was associated with a case of late-onset homozygous MVID which eventually achieved > 50% enteral nutrition (Müller et al. 2008; van der Velde et al. 2013). One identified mutation localizing to the cargo-binding domain, p.Leu1055dup, has been associated with better prognosis in multiple studies (Perry et al. 2014; Dhekne et al. 2018). In one case report, two compound heterozygous siblings both carrying p.Leu1055dup and p.Phe450Leufs*30 mutations were able to achieve enteral autonomy and histologic disease remission (Perry et al. 2014).

The treatment of MVID is particularly challenging. The initial management phase involves the correction of acute metabolic disturbances, including dehydration, metabolic alkalosis, and electrolyte levels. Oral feeding of these patients results in large volume diarrhea, and oral rehydration solutions are ineffective due to the mis-localization of SGLT1 (Engevik et al. 2018; Leng et al. 2020). Therefore, these patients most often require parenteral nutrition upon presentation and have life-long parenteral nutrition requirements with little hope for spontaneous enteral autonomy in most cases. As described above, a subset of patients with later onset disease may have a milder disease course and develop partial or complete enteral autonomy with age. There remains no definitive therapy for MVID, but patients are now able to survive past infancy. Fluid, electrolyte and acid/base management in *MYO5B* deficiency remains extremely challenging to manage and symptomatic therapies such as chloride channel blocking anti-diarrheal drugs may prove useful. Several promising leads have recently emerged from mouse models of MVID including lysophosphatidic acid and the Wnt/Notch pathway (Kaji et al. 2020, 2021). The next stage for this very severe and life-limiting disease is to advance and discover disease-modifying therapies that can restore even some amount of intestinal absorptive function.

### STXBP2

The first disease-causing variants of syntaxin-binding protein 2 (*STXBP2*) were found in patients with familial hemophagocytic lymphohistiocytosis type-5 (HLH5) by zur Stadt et al. in 2009 (zur Stadt et al. 2009). Like MVID, familial HLH has been mechanistically characterized as a disease of defective cellular trafficking, but primarily affects the traffic of cytotoxic granules in CD8 + T cells and NK cells (Canna and Marsh 2020). It was later demonstrated that a subset of familial HLH type-5 patients also displayed a severe congenital diarrhea phenotype, and subsequently that these patients displayed the histopathologic hallmarks of MVID (Pagel et al. 2012; Stepensky et al. 2013). As described above, STXBP2 acts as a vital adaptor protein in the final steps of the apical recycling pathway in intestinal epithelial cells. Intestinal manifestations of *STXBP2* variants display autosomal recessive inheritance with incomplete penetrance, while the familial HLH phenotype displays predominantly autosomal recessive inheritance with case reports of oligogenic inheritance (Stepensky et al. 2013; Zhang et al. 2014; Vogel et al. 2015).

As with *MYO5B*-associated disease which has a spectrum of disease that ranges from a phenotype of patients with only severe intestinal disease to patients with only moderate cholestasis, the spectrum of *STXBP2*-associated disease ranges from pure intestinal epithelial disease to pure familial HLH, with intermediate cases having features of both diseases. It has been well established that STXBP2 interaction with syntaxin-11 (STX11) is necessary for proper cytotoxic cell degranulation, while it appears that STXBP2 interaction with STX3 is necessary for proper apical recycling (Pagel et al. 2012; Vogel et al. 2015; Benavides et al. 2020). The Arg190 residue of STXBP2 appears to play a key role in STXBP2-STX11 interaction, as point mutations at this site result in a dominant negative phenotype of familial HLH type-5 without affecting the subcellular localization or expression of STXBP2, and without the development of brush border and trafficking defects (Zhang et al. 2014; Benavides et al. 2020). Equivalent conserved sites involved in STXBP2-STX3 interaction have not yet been identified. Structure–function data from missense mutations of *STXBP2* are lacking in the literature, as most missense mutations of *STXBP2* are predicted to result in dysfunctional protein folding and reduced expression (Dhekne et al. 2018).

At least some disease-associated variants of *STXBP2* display inconsistent disease phenotypes. In Pagel et al.’s initial paper describing gastrointestinal manifestations in patients with *STXBP2*-associated HLH, the absence of chronic diarrhea was linked to the presence of an exon 15 splice-site mutation, p.Val417Leufs*126; however, a later case report described a patient homozygous for this allele with chronic diarrhea and MVID-like symptoms (Pagel et al. 2012; Dhekne et al. 2018). Similarly, patients homozygous for the p.Pro774Leu mutation have been reported both with and without intestinal manifestations (Dhekne et al. 2018). Zhang et al. have demonstrated that many cases of familial HLH are due to oligogenic inheritance of multiple genes in the cytotoxic granule traffic pathway (Zhang et al. 2014). It is possible that a similar oligogenic inheritance pattern may explain the variable intestinal phenotypes seen in patients with *STXBP2* mutations.

Clinical management of familial HLH with intestinal symptoms involves the combined challenges associated with managing both MVID and HLH. The management of MVID is discussed above, while the management of familial HLH is discussed in numerous reviews (Henter et al. 2007; Canna and Marsh 2020; Griffin et al. 2020). The definitive management for all forms of familial HLH involves bone marrow transplant.

### STX3

Syntaxin-3 (STX3) is a t-SNARE protein which is vital for integration of the recycling endosome into the apical plasma membrane of intestinal epithelial cells (Knowles et al. 2014; Vogel et al. 2015). It was first implicated as a target of MVID-causing mutations by Wiegernick et al. in 2014, who identified the homozygous mutations p.Arg247* and p.Arg125Leufs*7 in two patients with MVID phenotypes but no detectable *MYO5B* mutations (Wiegerinck et al. 2014). Variants of this gene were subsequently reported in association with congenital retinopathy and intellectual disability without diarrhea (Chograni et al. 2015). Janecke et al. reported a cohort of 10 MVID patients with biallelic mutations in *STX3* (including cases recorded Wiegernick et al. and other authors), eight of whom were found to have severe retinal dystrophy (Janecke et al. 2021). The retinal phenotype of* STX3* variants is thought to be related to the trafficking of rhodopsin to rod photoreceptors (Mazelova et al. 2009).

Of the identified* STX3* variants, the only variant with intestinal-limited disease that has been identified is p.Arg247*. This variant was first reported in the initial case report (Wiegerinck et al. 2014) and was subsequently identified in a separate patient (Alsaleem et al. 2017), neither of which described retinal involvement. Overexpression of the p.Arg247* variant in Caco-2 cells resulted in disordered cellular polarity, suggesting a dominant negative effect. This mutation results in the introduction of a stop codon into the central SNARE domain of STX3, resulting in a product that lacks both the SNARE motif and the C-terminal transmembrane domain and is expressed in the cytoplasm rather than being membrane-bound (Wiegerinck et al. 2014; Dhekne et al. 2018).

*STX3* variants which have been identified as involving both intestinal and retinal disease make up the majority of cases, and are comprised primarily of early truncation and frameshift mutations, including p.Arg125Leufs*7, p.Tyr60Glnfs*16, and others (Janecke et al. 2021). Thus, it appears that the N-terminal domain of STX3 is important for preserving retinal function. This is supported by the retina-limited phenotype of the p.Glu41Gly mutation (Chograni et al. 2015). Identification of additional cases and further study of the STX3 interactome are needed to better understand the genotype–phenotype correlation of *STX3* variants. The management of the intestinal manifestations of *STX3*-associated diarrheal disease is similar to that of *MYO5B* disease and is discussed above. *STX3*-associated retinopathy is managed in a similar manner to congenital retinitis pigmentosa and is discussed in other reviews (Mendes et al. 2005; Wang et al. 2019).

### UNC45A

Unc45 myosin chaperone A (UNC45A) is a myosin co-chaperone required for adequate protein folding and expression of myosin Vb, among other myosins (Li et al. 2022; Duclaux-Loras et al. 2022). Disease-causing mutations in *UNC45A* were first described by Esteve et al. in a case series of three patients from four families with a syndrome of MVID-like diarrheal disease, cholestasis, impaired hearing, and bone fragility which was termed osteo-oto-hepato-enteric (O2HE) syndrome (Esteve et al. 2018). All identified cases of O2HE syndrome thus far have displayed autosomal recessive inheritance.

UNC45A is composed of an N-terminal tetratricopeptide repeat (TRP) domain, a central domain, and a C-terminal UCS domain. The TRP domain appears to be involved in the recruitment of heat shock protein-family chaperones Hsp70 and Hsp90 while the UCS domain has been shown to be critical for myosin binding (Barral et al. 1998; Scheufler et al. 2000; Esteve et al. 2018). A pair of very recent studies revealed the similarities in cellular phenotype between *UNC45A* mutations and *MYO5B* mutations in intestinal epithelial cells (Li et al. 2022; Duclaux-Loras et al. 2022).

Missense mutations, nonsense mutations, frameshift mutations, and splice variants have been described as disease-causing mutations for O2HE syndrome. Disease-causing missense mutations are distributed throughout the domains of the protein, but the majority fall within the central domain (e.g., p. Leu222Pro, p.Thr230Arg) (Esteve et al. 2018; Duclaux-Loras et al. 2022). Interestingly, all cases of O2HE with mild diarrheal symptoms and partial or complete weaning of parenteral nutrition have displayed compound heterozygosity involving a mutation in the distal UCS domain (p.Ala838Pro in one case and cis mutations p.Ser878Leu and p.Cys912Gly in two other cases). It is possible that disease-associated mutations in this region may portend a better prognosis for enteral autonomy; however, further study is required to determine if this trend holds true.

Management of the intestinal manifestations of *UNC45A*-associated diarrheal disease is similar to *MYO5B* mutations and is discussed above. Diagnosis and therapy for monogenic etiologies of hearing loss, including some etiologies due to defects in other UNC45A-associated myosins, are discussed in other reviews (Angeli et al. 2012; Wrobel et al. 2021).

### Congenital disease of glycosylation type 1b and MPI

Mannose-6-phosphate isomerase (MPI) is a zinc-dependent metalloenzyme which catalyzes the transformation of fructose-6-phosphate (F6P) to mannose-6-phosphate (M6P) (Eklund and Freeze 2006). This mannose-6-phosphate is then used in the synthesis of growing N-linked oligosaccharides to help direct traffic, folding, and degradation of various protein products. Autosomal recessive disease-associated mutations in *MPI* were first described in 1998 in a series of three independent publications. The symptoms of the condition were consistent across all publications: onset of protein-losing enteropathy before 1 year of age, cyclic or episodic vomiting, hepatic fibrosis of varying degrees, and a predisposition to thrombotic events (Niehues et al. 1998; de Koning et al. 1998; Jaeken et al. 1998). While MPI’s function is to catalyze a basic biochemical reaction, the disease caused by loss of MPI activity is best classified as a cellular trafficking disorder given the vital role of N-glycosylation to protein traffic through the secretory pathway.

The histology of duodenal biopsies in congenital disease of glycosylation (CDG) type 1b is characterized by partial villous atrophy with mild lymphangiectasia, while liver biopsy in these patients demonstrates congenital hepatic fibrosis. Ultrastructural analysis of hepatocytes in CDG type 1b is notable for lysosomal inclusions, presumably due to the accumulation of mis-trafficked proteins (Niehues et al. 1998; Lipiński and Tylki-Szymańska 2021). Definitive diagnosis is made by enzymatic assays targeting the function of MPI from freshly isolated fibroblasts or leukocytes with confirmatory *MPI* gene analysis (Čechová et al. 2020).

Disease-associated mutations are mostly missense mutations with some reported frameshift and splice-altering mutations. Most of the reported disease-associated missense mutations are either in close proximity to the active site (e.g., p.Met51Thr, p.Ser102Leu) or localize to the beta sheets which make up the core of the protein (e.g., p.Arg152Gln, p.Arg219Gln), with some localizing to the C-terminal domain (p.Ile398Thr, p.Arg418His) (Schollen et al. 2000). The mutation p.Met138Thr notably results in the replacement of one of the catalytic zinc-binding methionine residues (Jaeken et al. 1998). While it is likely that enteropathy in this disease is due to mis-localization of specific proteins, it is unclear which specific proteins are affected to produce this phenotype. In contrast, coagulopathy appears to be explained by the mis-trafficking of protein S, protein C, and antithrombin III (Jaeken et al. 1998). There has been little study of genotype–phenotype correlation in CGD type 1b and this represents an important area for future research.

CDG type 1b is notable for being one of the most treatable diseases of glycosylation, as the steps catalyzed by MPI may be circumvented simply by the oral or intravenous administration of exogenous mannose. This exogenous mannose is then converted to mannose-6-phosphate by mannokinase and may be utilized in N-linked oligosaccharide synthesis (Eklund and Freeze 2006; Čechová et al. 2020).

### CDG type 1h and ALG8

Disease-associated mutations in *ALG8*, a gene which encodes alpha-1,3-glucosyltransferase 8, were first reported by Chantret et al. in 2003. Clinical manifestations in this case were noted to be similar to those of CDG type 1b; however, MPI enzyme activity was found to be intact (Chantret et al. 2003). As with MPI-CDG, patients with ALG8-CDG have profound protein-losing enteropathy and hypercoagulability (Chantret et al. 2003; Eklund and Freeze 2006). Liver involvement, skeletal abnormalities, and developmental delay have also been described (Albokhari et al. 2022). Much like MPI, ALG8 regulates the production of N-linked oligosaccharides which are conjugated to proteins to direct traffic through the secretory pathway. Unfortunately, as with MPI-CDG, it remains unclear which specific proteins are mis-trafficked.

The reported cases of *ALG8*-associated CDG in the literature are made up of missense mutations, nonsense mutations, and splice mutations. The most commonly reported mutation is p.Thr47Pro (Höck et al. 2015). Paradoxically, missense mutations in *ALG8* have been associated with a poorer prognosis while multiple patients with nonsense and splice mutations have displayed long-term survival (Stölting et al. 2009). In one case, a splice variant (c.1434delC) was found to affect the dominant transcript variant of *ALG8*, but to spare the second most common transcript, presenting a possible explanation for this improved prognosis.

The range of possible disease manifestations in ALG8-CGD is quite large, and so a thorough evaluation for involved organ systems is necessary at the time of diagnosis. As with many cases of protein-losing enteropathy, therapy involves albumin supplementation and management of peripheral edema (Braamskamp et al. 2010; Levitt and Levitt 2017). The role of bowel transplant in the enteropathy-associated CDGs has yet to be established.

### MEDNIK syndrome and AP1S1

Adaptor protein-1 complex subunit sigma-1 (AP1S1) is a subunit of the adaptor protein-1 complex (AP-1), which is necessary for clathrin coat assembly at the trans-Golgi network and endosomes (Duncan 2022). This subunit is thought to play a role in stabilization of the AP-1 complex (Montpetit et al. 2008). Montpetit et al. determined that variants of *AP1S1* were associated with MEDNIK syndrome, which had previously been described as a clinical entity in four French-Canadian families by Saba et al. (Saba et al. 2005; Montpetit et al. 2008). MEDNIK syndrome is characterized by developmental delay, enteropathy, deafness, neuropathy, ichthyosis, and keratodermia. The diarrhea in MEDNIK syndrome presents within the first week of life with profound dehydration and salt-wasting (Klee et al. 2020). Interestingly, these patients also display abnormalities of copper transport leading to hypocupremia and liver copper accumulation similar to that seen in Wilson’s disease (Martinelli et al. 2013).

Knockout of AP1S1 or sole expression of patient-derived variants p.Leu90Pro and p.Glu116Lys in CaCo-2 cells results in preferential mis-localization of ZO-1 and claudin-3 to the basolateral membrane (Klee et al. 2020). The resultant loss of function of tight junctions is thought to be the ultimate etiology of enteropathy in these patients. Given the rarity of this disease and the paucity of biopsy samples from these patients, the loss of ZO-1 and claudin-3 localization has yet to be validated by staining of a patient sample.

Similar to patients with MVID, patients with MEDNIK syndrome develop profound diarrhea and metabolic disturbances within the first days of life, necessitating early correction of acid–base and electrolyte imbalances and the initiation of parenteral nutrition. Some of these patients have been able to wean off parenteral nutrition with time (Torres et al. 2019).

### Trichohepatoenteric syndrome and TTC37

Trichohepatoenteric syndrome (THES), also referred to as “syndromic diarrhea” or “phenotypic diarrhea of infancy”, is a multisystemic disease that is inherited in an autosomal recessive fashion and affects around 1 in 1,000,000 live births (Hartley et al. 2010; Fabre et al. 2011). In 60% of cases, the phenotype historically described as THES is caused by mutations in the *TTC37* gene, which encodes a cytosolic protein known as thespin (Fabre et al. 2011). The presentation of children with disease-associated *TTC37 *variants is very broad and can include moderate to severe enteropathy, failure to thrive, eczema, facial dysmorphism, recurrent upper respiratory and gastrointestinal tract infections, liver disease, hemolytic anemia, thrombocytopenia accompanied by other platelet abnormalities, cardiac defects, alopecia, and trichorrhexis nodosa (Fabre et al. 1993). Affected infants generally require parenteral nutrition, but there have been reports of enteral autonomy acquired with age (Hartley et al. 2010). THES is also associated with an immune dysregulation phenotype which has been compared to immune dysregulation, polyendocrinopathy, enteropathy, X-linked (IPEX)-disease, and Hyper IgM syndrome (Baxter et al. 2022; Kristal et al. 2022).

Histopathological analysis of biopsy specimens of the small intestine of THES patients has demonstrated varying degrees of villous atrophy and mixed inflammatory infiltrates, with a structurally normal enterocyte brush border (Landers 2003; Hartley et al. 2010). Immunohistochemical studies performed on intestinal and liver biopsies of patients with confirmed *TTC37* mutations demonstrate a wide variety of cell polarity and signaling alterations. While the Na^+^/K^+^ ATPase is appropriately expressed in the basolateral membrane, there have been reports of alterations in the localization of the Na^+^/H^+^ exchangers and the Na^+^/I symporter. Additionally, reductions in the apical expression of H^+^/K^+^ ATPase and absence of AQP7 have been described (Hartley et al. 2010).

Thespin is a component of the Ski complex, a protein complex which is a cytoplasmic cofactor of the RNA exosome complex and is involved in cytoplasmic mRNA degradation in the case of stop loss or gain. It plays a vital role both in cell housekeeping and antiviral responses (Bourgeois et al. 2018). It has been suggested that the immunologic phenotype in THES is due to the loss of thespin endonuclease activity which negatively regulates Rig-like receptors, resulting in Rig-like receptor overactivity and excessive type 1 interferon signaling (Eckard et al. 2014). However, the mechanism by which loss of thespin function results in mis-trafficking of apical transporters remains to be elucidated.

Most disease-causing mutations observed in* TTC37* are nonsense mutations, with a minority of splice-site mutations, missense mutations, and insertion or deletion-induced frameshifts. The most common mutations are p.Trp936* (which is found in families from Pakistan and India) and p.Trp1524* (which is found in families from Turkey) (Bourgeois et al. 2018). Notably, the p.Trp1524* mutation results in a late truncation, close to the C-terminus of the protein, and was found to be associated with improved survival and lower requirements of parenteral nutrition (Esteve et al. 2018). Another notable mutation, p.Lys1446_Ala1447insLeu, was found in association with p.Arg710* in a patient with immune dysregulation but without significant diarrhea (Rider et al. 2015). This suggests that this mutant protein may have residual activity in the intestinal epithelium while impairing the function of immune cells. Further study of such cases is necessary to better understand the differential function of TTC37 in epithelial and immune cells.

Patients with THES often have large parenteral nutrition requirements upon presentation; however, 30 and 50% of patients may achieve enteral autonomy after prolonged PN (Fabre et al. 2018). These patients are also at high risk of humoral immunodeficiency and should be assessed on presentation for active infection and for immune system dysfunction. Intravenous immunoglobulin therapy has been successful in the treatment of this immunodeficiency (Rider et al. 2015). Of note, hematopoietic stem cell transplant has not yielded significant improvement in outcomes for these patients (Kammermeier et al. 2014).

### SKIV2L

*SKIV2L* was identified as a disease-associated gene by Fabre et al. in a cohort of 6 patients with clinical and histopathologic THES, but without mutations in TTC37 (Fabre et al. 2012). Like thespin, SKIV2L is a member of the Ski complex, described above. The clinical manifestations of *SKIV2L*-associated THES are identical to those of TTC37-associated THES. Structurally, SKIV2L harbors a helicase domain and a helicase-associated ATP-binding domain (Bourgeois et al. 2018).

While most patients with *SKIV2L*-THES are homozygous or compound heterozygous for disease-causing alleles, a minority of patients with heterozygous genotypes and a dominant-negative allele have been described and account for approximately 10% of cases (Bourgeois et al. 2018). Disease-associated missense, nonsense, and frameshift mutations are all roughly equally frequent, with a minority of splice-site mutations reported in the literature. Most observed missense mutations are within the helicase ATP-binding domain, spanning exons 10 to 14. In contrast, reported nonsense mutations are distributed throughout the protein. As with *TTC37*, disease-causing variants of *SKIV2L* with immunologic manifestations, but without enteropathy, have been reported. One patient with heterozygous p.Gln302* and p.Arg888Glyfs*12 displayed this phenotype, however the exact mechanism by which this patient escaped intestinal manifestations remains unclear (Poulton et al. 2019).

### Tufting enteropathy and EPCAM

The *EPCAM* gene encodes the epithelial cellular adhesion molecule (EpCAM) protein, which is normally expressed on the basolateral membrane of cells in epithelial tissues and on plasma cells (Pathak et al. 2019). *EPCAM* mutations have been associated with congenital tufting enteropathy, an autosomal recessive disease with a described incidence of 1 in 50,000–100,000 live births in Western Europe (Goulet et al. 2007; Sivagnanam et al. 2008). They have also been associated with Lynch syndrome, a tumor syndrome caused by deficiencies in the mismatch repair pathway (Pathak et al. 2019). Lynch syndrome is a form of hereditary non-polyposis colon cancer (HNPCC) syndrome, which also manifests with increased risk for extracolonic malignancies.

**Fig. 5:**
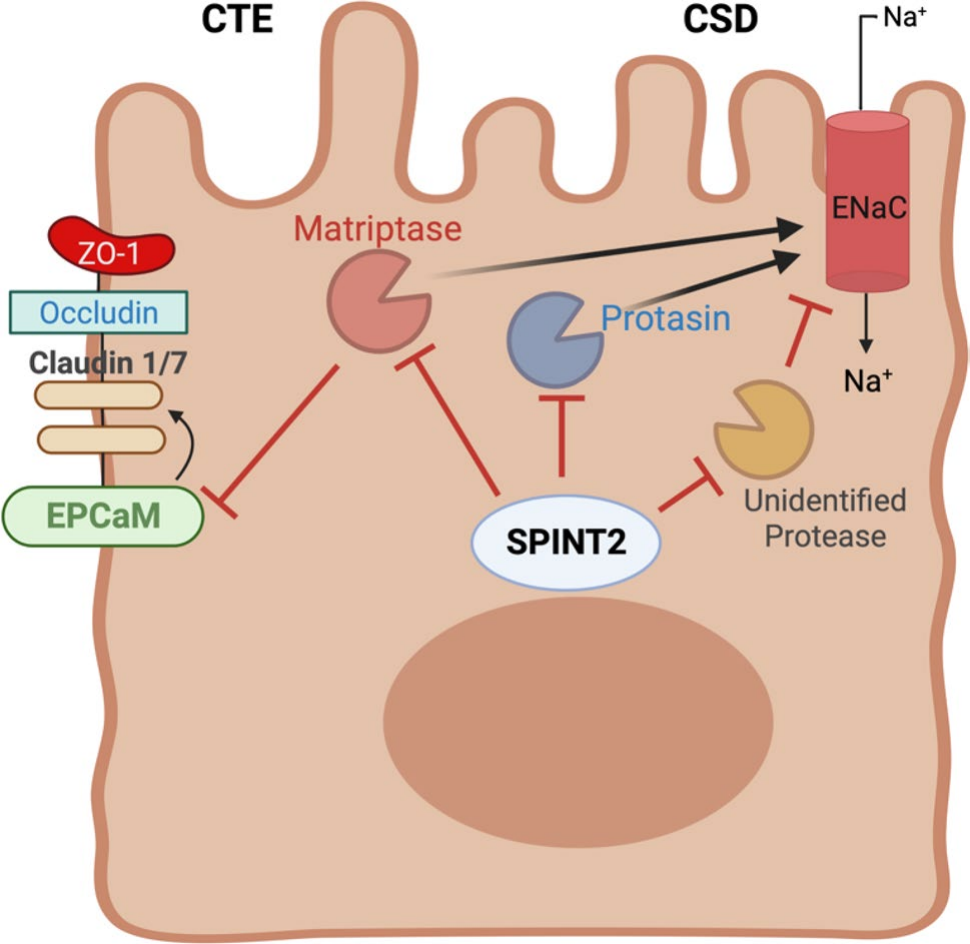
Epithelial structural and functional pathways associated with EpCAM and SPINT2 function. ENaC (Epithelial sodium channel), CTE (congential tufting enteropathy), CSD (congential sodium diarrhea), ZO-1 (Zona occludens protein 1)

Congenital tufting enteropathy (CTE), also known as intestinal epithelial dysplasia, presents in the first days of life with life-threatening intractable watery diarrhea, electrolyte disturbances, and failure to thrive (Sivagnanam et al. 2008; Kozan et al. 2015). On duodenal and colonic histology, CTE is characterized by a lack of inflammation and presence of villous atrophy, branching crypts, and the pathognomonic epithelial “tufts” of focally crowded enterocytes located at the tips of villi (Reifen et al. 1994; Goulet et al. 2007). In T84 cells, knockdown of *EPCAM* results in decreased expression of tight junction proteins ZO-1 and occludin, as well as the transporter NKCC1 and this disturbance of tight junction proteins and transporters may explain some of the disease phenotype (Fig. 5). More recent studies have suggested that altered epithelial cell differentiation may also play a major role in disease manifestations (Das et al. 2021).

Many CTE-associated mutations of *EPCAM* represent functional knockout of the protein. The most common CTE-causing mutation in *EPCAM* is c.499dupC, which results in a frameshift and is associated with a poorer overall prognosis compared to other variants. The p.Ala18_Gln24del mutation disrupts a leader peptide targeting of *EPCAM* to the cell membrane, while missense mutations preferentially affect the thyroglobulin homology domain or extracellular C-terminal domain and are thought to disrupt cell–cell interactions (Pathak et al. 2019). It is the loss of EpCAM-mediated cell–cell interaction which has been proposed to be the ultimate mechanism for enteropathy in CTE. In contrast, monoallelic deletions of the 3' end of EPCAM lead to the development of Lynch syndrome via epigenetic silencing of *MSH2* (Sehgal et al. 2014). In cases where the *EPCAM* polyadenylation sequence is lost, but the *EPCAM* protein-coding sequence and *MSH2* promoter are preserved, there is an *MSH2*-Lynch phenotype without CTE (Kloor et al. 2011; Lynch et al. 2011). Notably, there have been no overlap cases of CTE and Lynch syndrome reported thus far, however this may be due to masking of the Lynch phenotype by the high early-life mortality of CTE.

Therapeutically, CTE patients require rapid initiation of parenteral nutrition upon presentation, and most patients will never achieve enteral autonomy (Goulet et al. 2007; Das and Sivagnanam 2020). For patients with bowel transplantation the prognosis is even worse, with a 3-year survival rate of only 30% (Das and Sivagnanam 2020; Ozler et al. 2021).

### SPINT2

Serine protease inhibitor, Kunitz type 2 (SPINT2) is a negative regulator of multiple endogenous serine proteases. Disease-associated *SPINT2* mutations were first identified in the context of a patient with congenital sodium diarrhea in 2009 (Heinz-Erian et al. 2009). The following year, Sivagnanam et al. identified a patient with tufting enteropathy who lacked a mutation in *EPCAM* but was homozygous for a mutation in* SPINT2* (Sivagnanam et al. 2010). Congenital sodium diarrhea (CSD) was characterized as an autosomal recessive disorder with neonatal large volume watery diarrhea with high fecal sodium (Müller et al. 2000). Patients with *SPINT2*-mutation have additional features, including choanal atresia and corneal erosions. For this reason, CSD that is associated with these features is sometimes called “syndromic” CSD (Janecke et al. 2016). Meanwhile, the form of CTE associated with *SPINT2* mutations is similar to that associated with *EPCAM* mutations but includes the additional symptoms of systemic keratitis and choanal atresia (Salomon et al. 2014). This has been referred to as “syndromic” CTE (Sivagnanam et al. 2010). Both “syndromes” represent variations in the respective disease phenotype associated with *SPINT2* mutations and therefore can be thought of a part of the spectrum of *SPINT2* deficiency.

Ultimately, sodium-rich diarrhea is due to the mis-localization, absence, or dysregulation of sodium transporters on the intestinal epithelium—particularly NHE3 and ENaC (Müller et al. 2000). The classical form of CSD is due to the primary loss of NHE3 function, encoded by *SLC9A3* and described in the section on disorders of epithelial transporters. In the healthy intestinal epithelium, serine proteases regulate both ENaC and EpCAM. The membrane-anchored serine proteases prostasin and matriptase are converted to their active forms by reciprocal zymogen activation, linking their functionality. Prostasin and matriptase both potently activate ENaC by cleaving in the γ-subunit and increasing the channel’s open probability (Wakida et al. 2006; Bruns et al. 2007; Kota et al. 2012). Matriptase also negatively regulates EpCAM by cleaving the protein and targeting it for lysosomal degradation (Wu et al. 2017). SPINT2 serves as an endogenous inhibitor of both proteases, but in the case of *SPINT2* disease, the ability of SPINT2 to inhibit these serine proteases is lost. There may also be yet unidentified SPINT2 targets which play a role this pathology.

All reported *SPINT2*-deficiency patients with histological features of CTE so far have had genotypes with at least one hypomorphic mutation resulting in residual functionality, and constitutive knockout of *Spint2* in mice is embryonic lethal. Therefore, it is likely that some residual SPINT2 activity is necessary to avoid embryonic lethality (Kawaguchi et al. 2019; Cai et al. 2020). The most commonly identified mutation in *SPINT2* disease is p.Tyr163Cys (Janecke et al. 2016). This mutation and nearby missense mutations in the second Kunitz domain (KD-2) of SPINT2, p.Arg148Cys, p.Phe161Val, and p.Gly168Ser, result in the inability of SPINT2 to inhibit prostasin without affecting its ability to inhibit matriptase (Wu et al. 2017; Hirabayashi et al. 2018; Holt-Danborg et al. 2019). However, as these proteases undergo reciprocal zymogen activation, these mutations may still impair the biologic activity of matriptase by reducing its conversion to an active conformation (Friis et al. 2013).

It is still unclear how *SPINT2* mutations lead to CSD-predominant features versus CTE features, or vice versa. Homozygosity for missense mutations in the KD-2 domain is more associated with a CSD phenotype; however, patients homozygous for p.Tyr163Cys and other KD-2 missense mutations are present in both populations (Heinz-Erian et al. 2009; Salomon et al. 2014). Meanwhile, patients with compound heterozygosity for a KD-2 domain mutation in combination with a frameshift or loss-of-start codon tend to be associated with a CTE phenotype. Of note, there are patients with clinical CTE without mutations in *EPCAM* or *SPINT2*, suggesting that unknown genetic etiologies of CTE remain undiscovered. Overall, the genotype–phenotype correlation in *SPINT2*-associated disease is still poorly understood, and further study is necessary to determine the molecular etiology of a CTE, CSD, or mixed CTE–CSD phenotype.

Management of *SPINT2*-deficiency is dependent on the predominant phenotype. SPINT2-CTE, is managed as with EPCAM-CTE with an emphasis on nutrition, and growth as described above, with additional surveillance required for other features that require clinical attention such as choanal atresia, corneal involvement, and skin involvement, among others (Salomon et al. 2014). Likewise, treatment for SPINT2-CSD is similar to that of NHE3-CSD but requires similar attention to associated syndromic features.

### Multiple intestinal atresia and TTC7A

Tetratricopeptide repeat domain 7A (TTC7A) was discovered to be the causative gene for congenital disorder with a spectrum of abnormalities including multiple intestinal atresias (MIA), enteropathy, intestinal inflammation, variety of skin and hair manifestations, and a variable severity combined immunodeficiency. In parallel in 2013, three groups reported TTC7A mutations in cohorts of patients with intestinal atresias, combined immunodeficiency and intestinal inflammation. Samuels et al. demonstrated the presence of *TTC7A* mutations in MIA patients from seven French-Canadian families using whole-exome sequencing, along with studies by Avitzur et al. and Chen et al. in cohorts of patients with inflammatory bowel disease and combined immunodeficiency, respectively (Samuels et al. 2013; Chen et al. 2013; Avitzur et al. 2014).

TTC7A is a scaffolding protein containing nine tetratricopeptide (TRP) repeats. It has been shown to be important in the membrane localization and generation of phosphoinositides by chaperoning phosphatidylinositol-4-kinase (PI4K)-IIIα to cellular membranes (Avitzur et al. 2014; Bugda Gwilt and Thiagarajah 2022). The correct sequence and timing of the generation of various phosphoinositide species at vesicular and plasma membranes is known to be critical in the normal de novo polarization of intestinal epithelial cells and in the correct formation of the intestinal and glandular lumens (Bugda Gwilt and Thiagarajah 2022). Consequently, loss-of-function mutations in *TTC7A* lead to profound abnormalities in intestinal epithelial polarity and lumen formation. This results in a highly architecturally distorted epithelium particularly in the colon characterized by stratified or pseudostratified epithelium, formation of crypt glands with multiple lumens, and mis-localization of apical and basolateral markers (Bigorgne et al. 2014; Jardine et al. 2018; Dannheim et al. 2022). In addition to epithelial polarity defects, TTC7A also plays a role in stabilization of the Akt survival signaling pathway (Jardine et al. 2018). Therefore, loss of TTC7A function results in decreases in Akt phosphorylation, increased caspase-3 cleavage and subsequent increased susceptibility to apoptosis.

Clinically, missense mutations of *TTC7A* tend to be associated with fewer atresias, less severe immunodeficiency, and a more enterocolitis predominant phenotype with less morbidity and mortality compared to patients who have homozygous nonsense mutations, frameshifts, and splice-site mutations that result in effective gene knockout (Jardine et al. 2018). These missense mutations make up most variants that have been associated with disease manifestations (Lien et al. 2017). The most common disease-associated missense variant of *TTC7A* is p.Glu71Lys; however, all 13 cases of homozygosity for this variant were from one large French family with consanguinity (Lien et al. 2017). These patients manifested with CID and inflammatory bowel disease, but without MIA. In the initial case series linking *TTC7A* variants to MIA, five patients from four French-Canadian families all carried the same homozygous 4 bp deletion involving the donor splice site of exon 7 (Samuels et al. 2013). This variant was linked to an MIA phenotype in four homozygous patients and to MIA-CID in two heterozygous patients.

There remains no standardized management of *TTC7A* deficiency given the wide spectrum of disease, and the difficulty in managing the many clinical issues present. Surgical interventions have been attempted to correct the associated atresias; however, these resections do not prevent the formation of new atresias or always result in improvement of long-term outcomes (Jardine et al. 2018; Culbreath et al. 2022). Hematopoietic stem cell transplant is generally effective at addressing immunodeficiency in these patients, but overall outcomes have been equivocal due to the fact that this does not improve the intestinal disease phenotype (Kammermeier et al. 2016). Recent studies from the Muise group in Toronto in collaboration with our lab identified the drug leflunomide as a possible therapeutic for epithelial dysfunction in *TTC7A* deficiency. Leflunomide was able to improves survival and reduces intestinal tract narrowing in *TTC7A* knockout zebrafish model and improve apicobasal polarity and transport function in *TTC7A deficient* patient-derived colonoids (Jardine et al. 2020). As leflunomide is an already FDA-approved medication with previous use in pediatric patients initial *n* = 1 therapeutic trials in *TTC7A* patients are now underway with some initial promising results. However, its formal effectiveness remains to be determined in sufficient patients to draw conclusions and long-term feasibility for treatment of *TTC7A* intestinal disease, therefore, remains uncertain.

## Enteroendocrine function

Enteroendocrine cells are a critical secretory epithelial cell population in the intestine that play a variety of regulatory and sensory roles. Several CODE disorders (*NEUROG3*, *PCSK1*, *PERCC1*) stem from genes largely involved in normal enteroendocrine-cell development / differentiation resulting in a loss of this cell population and provided insights on their importance for normal fluid and nutrient absorption.

### Enteric anendocrinosis and NEUROG3

Neurogenin-3 (*NEUROG3*) is a gene located in chromosome 10q22.1 which plays a critical role in the differentiation of enteric stem cells and pancreatic progenitor cells. Homozygous missense mutations on this gene were first described to be causative of human disease in 2006 in a group of patients who presented with intractable malabsorptive diarrhea after initiation of feeds (Wang et al. 2006). Loss of *NEUROG3* function results in defective development of intestinal enteroendocrine cells, and therefore, the disorder has also been referred to as ‘enteric anendocrinosis’. The disorder resulting from *NEUROG3* mutations is extremely rare, with only 10 reported cases in the literature to date.

Enteric anendocrinosis is characterized by severe malabsorptive diarrhea of neonatal onset, which, unless properly addressed, can result in dehydration and metabolic acidosis; additionally, patients can suffer from childhood-onset diabetes (Rubio-Cabezas et al. 2011). The absence of Ngn3 leads to the loss of α-, β-, δ-, and pancreatic polypeptide cells, along with the respective hormones those cells produce; nonetheless, intestinal biopsies reveal a normal crypt-to-villous ratio (Gradwohl et al. 2000). Wang et. al described three patients who suffered from malabsorptive congenital diarrhea with an unclear cause, who had to be placed on parenteral nutrition and limited enteral feedings. Both patients who survived past infancy ended up developing type 1 diabetes mellitus. While biopsy samples in these patients revealed a normal villous structure, staining for chromogranin A showed defective formation of enteroendocrine cells, with a lack of synaptophysin (Wang et al. 2006).

*NEUROG3* encodes for neurogenin-3 (Ngn3), a basic helix–loop–helix transcription factor with a well-established role in neurogenic development. Studies involving mice have proven that neurogenin-3 is essential for the differentiation of all pancreatic endocrine precursors (Gradwohl et al. 2000). Downstream targets of neurogenin-3 include NeuroD, Pax4, and Nkx2.2, all of which contribute to completing the differentiation process of pancreatic precursor cells (Jensen 2004). Ngn3’s transcription is repressed by high levels of Notch signaling, and crosstalk between members of the Notch signaling pathway and Ngn3 important for coordinating intestinal differentiation in a timely and organized manner (Li et al. 2012). Sequencing in the three affected patients who were initially described showed that they suffered from homozygous missense loss-of-function mutations in the *NEUROG3* gene (Wang et al. 2006). These mutations resulted in an arginine to serine substitution at position 107 (p.Arg107Ser), which corresponds to the first helix of the protein, in one patient, and in an arginine to leucine substitution upstream at position 93 (p.Arg93Leu), in the DNA-binding region, in the other two patients. Both mutations rendered Ngn3 incapable of activating NeuroD1, which is a downstream transcription factor that regulates the expression of the insulin gene.

The establishment of neurogenin-3 as a crucial determinant for regulating human pancreatic and intestinal endocrine cell development represents an area of opportunity for research to understand the role of these cells in intestinal transport, sensory, and developmental function (Zeve et al. 2022).

### PERCC1 deficiency

The *PERCC1* gene, mapped to chromosome 16p13.3, codes for the proline and glutamate-rich protein with coiled-coil domain 1 (PERCC1). *PERCC1* was very recently found to be the causative gene in a series of cases with unknown infantile-onset malabsorptive diarrhea and failure to thrive. Uniquely, the discovery of loss of *PERCC1* function was made by analysis of a non-coding region of the genome found commonly deleted in the case series (Oz-Levi et al. 2019).

*PERCC1* flanks a genomic region named the “intestinal critical region (ICR)”, a non-coding sequence that was subsequently found to control expression of *PERCC1*. This finding came after eight patients of Jewish Iraqi origin who presented with severe congenital malabsorptive diarrhea were found, by whole-exome and whole-genome sequencing, to have a homozygous deletion of the ICR. The ICR was defined as the minimal 1,528 base pair overlapping region between the homozygous 7,013-bp deletion and the compound heterozygous 3,101-bp deletion. As expected, analysis of single-cell transcriptomes from the murine intestine identified Percc1-positive cells as enteroendocrine, with expression of Sox and Ngn3. Analysis of RNA sequencing data from *Percc1* mouse knockout models demonstrated significant stomach and intestinal downregulation of many hormones dependent on enteroendocrine-cell production, such as gastrin and somatostatin, with upregulation of certain proinflammatory genes. Interestingly, human intestinal organoids generated from affected patients initially showed no morphological abnormalities, but later, by day 42, a significant decrease was seen in the number of enteroendocrine cells in *PERCC1* patient-derived organoids. This suggests that the role of *PERCC1* may occur later in enteroendocrine development.

Recently, computational analysis of the PERCC1 protein sequence has shown that PERCC1 is a YAP (Yes-associated protein), TAZ (transcriptional coactivator with a PDZ-binding domain), and FAM181 homologue; these transcriptional regulators bind to DNA through TEADs (TEA/ATTs domain) (Sanchez-Pulido et al. 2022). The structural homology of PERCC1, with conservation of TEAD-binding interfaces-2 and -3 now yields valuable insight into the previously unknown molecular mechanism of the protein. Future research with PERCC1 cell lines might be able to clearly describe in detail the interaction between PERCC1 and TEAD (TEA/ATTS domain) transcription factors.

### PCSK1 deficiency

The human *PCSK1* gene, located in chromosome 5q15, contains 14 exons, is localized on chromosome 5q15, and codes for subtilisin-like proprotein convertase-1 and proprotein convertase-3 (PC1/3). A calcium-dependent serine endoprotease required for the biosynthetic processing of hormone and neuropeptide precursors and their transformation into their fully functional forms (Jean et al. 1993; Martín et al. 2013). PC1/3 can be found in the hypothalamus, the endocrine portion of the pancreas, and the stomach (Stijnen et al. 2016). It is synthesized as a propeptide which undergoes autocatalytic cleavage and is eventually stored in mature secretory granules (Muller and Lindberg 1999).

Initially, the gastrointestinal phenotype resulting from* PCSK1* mutations was underrecognized, due to a focus on childhood obesity (Martín et al. 2013). The first two cases of PC1/3 deficiency linked to congenital diarrhea were described in 2003, one of which was a patient previously characterized solely based on her endocrinological manifestations but who later, with subsequent re-evaluation, reported a mild–moderate degree of small intestine dysfunction (Jackson et al. 2003). To date, around 30 cases of *PCSK1* deficiency have been identified and reported in the literature (Aerts et al. 2021). As with many other CoDEs, *PCSK1* mutations are life-threatening in early infancy and can require prolonged parenteral nutrition, although in most cases this can be weaned by 2 years of age (Aerts et al. 2021). Characteristically, most patients have normal intestinal biopsies; nonetheless, mild villous atrophy without evidence of inflammation has also been described in some cases (Martín et al. 2013).

In contrast to the phenotype caused by *NEUROG3* loss-of-function mutations in enteric anendocrinosis, patients who carry *PCSK1* loss-of-function mutations have not been found to develop diabetes mellitus. However, they can present with other severe endocrinopathies, such as adrenal insufficiency, hypothyroidism, hypercortisolism, hypogonadotropic hypogonadism, and obesity in late infancy, in addition to early-onset malabsorptive diarrhea (Martín et al. 2013; Aerts et al. 2021).

Nonsense, missense, frameshift, and splice site mutations of *PCSK1* have been described. Sequencing in a cohort of 13 patients with homozygous *PCSK1* mutations yielded valuable information on the effect of different *PCSK1* point mutations, with most resulting in a lack of enzymatic activity, even when the proteins were correctly synthesized (Martín et al. 2013). The most severe clinical picture resulted from two nonsense mutations: p.Met1* mutation, which deleted the initiation codon, and the p.Arg405* mutation, which deleted the protein’s P- and C-terminal domains. The only frameshift variant identified, p.Val450fs*1, produced a truncated PC 1/3 protein, while a p.Pro258Thr substitution did not result in an alteration of the protein’s secretion or of its enzymatic activity, suggesting that it may affect the catalytic domain in a region that is not critical for protein folding.

Recently, a male newborn whose clinical picture was compatible with* PCSK1* deficiency was found, through whole-exome gene sequencing, to carry a previously undescribed homozygous c.1034A → C (p.Glu345Ala) point mutation in exon 8 of the *PCSK1* gene (Aerts et al. 2021). Given that the Glu345 residue is located in the catalytic domain of PC1/3, in vitro studies were conducted with mutant PC 1/3 to assess its role in enzymatic activity; results showed that the mutation did not impact autocatalytic activation but rendered PC1/3 unable to carry out trans cleavage. Other homozygous mutations identified previously, such as Ser307Leu and Asn309Lys, appear to not affect intracellular trafficking and maturation, but lead to a loss of enzymatic activity (Farooqi et al. 2007; Wilschanski et al. 2014). Future genetic analyses of specific *PCSK1* mutations might allow for a better characterization of this genotype–phenotype relationship.

### Mitchell-Riley syndrome and RFX6

The *RFX6* gene, mapped to chromosome 6q22.1, consists of 19 exons and codes for RFX6, a transcriptional regulatory factor that functions downstream of neurogenin-3 and participates in the development of the pancreas, intestine, and gallbladder (Aftab et al. 2008). *RFX6* mutations result in Mitchell–Riley syndrome, an extremely rare but potentially fatal autosomal recessive clinical condition, with around 20 cases reported in the literature, characterized by intestinal atresia, malrotation, progressive cholestasis, neonatal diabetes, pancreatic hypoplasia, and protracted congenital diarrhea (Smith et al. 2010). The condition was initially described in a set of five patients who presented with similar clinical manifestations, albeit of different severity (Mitchell et al. 2004). In the ensuing quest for a molecular explanation, *RFX6* was found to be involved in the disease directing differentiation of endocrine cells including pancreatic islet cell types, with the exception of pancreatic polypeptide-producing cells. It was detected in the definitive endoderm early in development, later localized to the gut and pancreatic bud, and eventually restricted to the pancreatic islets in the mature pancreas. The degree of pancreatic and gallbladder involvement in Mitchell-Riley Syndrome appears to be associated with the specific *RFX6* gene mutation, with some patients retaining residual pancreatic activity, thereby suggesting a genotype–phenotype correlation (Kambal et al. 2019). Recently, congenital glucagon-like peptide-1 (GLP-1) deficiency was identified in several Mitchell-Riley syndrome patients, suggesting a potential pathogenic explanation for the diarrhea; synthetic GLP-1 analog liraglutide was administered, resulting in diarrhea improvement in all patients, with no effect on glycemic status (Nóbrega et al. 2021). These findings warrant further investigation, as they might hold the key to managing this condition successfully.

### X-linked lissencephaly and ARX

The *ARX* gene is a small ~ 12.5 kb, 5-exon gene mapped to chromosome Xp21.3 which codes for aristaless-related homeobox, X-linked, a paired domain transcription factor that plays a role in the development of the human brain, testes, and pancreas (Strømme et al. 2002). Described initially in 2002, multiple mutations have been identified, producing heterogeneous clinical pictures of different severity (Strømme et al. 2002; Bienvenu et al. 2002; Kitamura et al. 2002). These mutations have been associated with the development of different pathologies characterized mainly by neurologic malformations and intellectual disability, such as X-linked lissencephaly with abnormal genitalia, West Syndrome, and Partington Syndrome. Additionally, the clinical picture may include severe congenital diarrhea (Kato et al. 2004). Mutant *Arx* loss-of-function mice present with severe hypoglycemia, dehydration, and death within the first few days of life; a close look into their pancreas shows that the glucagon-producing alpha-cell population is practically non-existent, with a marked increase in beta- and delta-cell populations, serving as evidence to* ARX*’s role in defining the fate of endocrine cells. It was also found in this study that *ARX* and *PAX4* play antagonistic mutually inhibitory roles in tightly determining the destiny of endocrine progenitors (Collombat et al. 2003). Duodenal biopsies in *ARX* patients and loss-of-function mice with a gastrointestinal phenotype have revealed a selective decrease in GLP-1 and CCK, suggesting enteroendocrine dysgenesis as a potential cause for the diarrhea in these patients (Terry et al. 2015).

The different types of *ARX* mutations identified include splice site, nonsense, missense, deletions, insertions, and duplications, with most being the result of expansions in the first two out of four polyalanine tracts (Shoubridge et al. 2010). The most common mutation accounting for about 40% of all *ARX* mutations is c.429_452dup, which expands the second polyalanine tracts from 12 to 20 alanine residues.

A genotype–phenotype correlation has been observed with *ARX* mutations, with the most severe manifestations arising from truncation and missense mutations that cause amino acid substitutions in the homeodomain region, whereas the least severe clinical picture without brain malformation results from polyalanine expansion and certain missense mutations (Kato et al. 2004; Shoubridge et al. 2010). Unfortunately, despite the progress that has been made to obtain a better understanding of the molecular pathogenic mechanisms of *ARX* mutations, there is still no targeted therapy available for these patients.

## Epithelial stem cell function

Intestinal epithelial stem cells situated at the base of intestinal crypt glands drive the production of all lineages and types of epithelial cell in development, homeostasis and in response to injury. Somatic mutations that accumulate in stem and progenitor cell populations are also thought to underlie intestinal neoplasia. Given the critical importance of stem cell maintenance, germline mutations in genes involved in regulating and tuning stem cell function rather than in genes fundamental to their preservation are found to cause a relatively recent category of CoDE disorders. As with CoDE disorders involving trafficking and polarity, mutations in genes such as* WNT2B* have revealed interesting and surprising insights into the normal early development of the human intestinal mucosa.

### WNT2B deficiency

The *WNT2B* gene, mapped to chromosome 1p13.2, codes for WNT2B, a protein previously known as WNT13, which is a member of the wingless-type MMTV integration site family and is involved in developmental pattern formation, differentiation, and cell growth (Katoh et al. 1996; Wakeman et al. 1998). Wnt proteins are well known to be important in development and in intestinal epithelial stem cell renewal and maintenance.

The significance of *WNT2B* in intestinal biology was relatively poorly understood until the finding of human *WNT2B* mutations linked to intestinal disease. In the original study O’Connell et al. identified a series of three patients with infantile-onset chronic diet-induced diarrhea and failure to thrive that were subsequently found to harbor homozygous nonsense variants in *WNT2B* (O’Connell et al. 2018). Histopathologic analysis of intestinal biopsies taken from these patients showed a disruption of normal crypt architecture in the colon and small intestine with a paucity of crypt glands, and decreased protein expression of the intestinal epithelial stem cell marker *OLFM4* (van der Flier et al. 2009). The patients also showed distinct histological changes in the stomach with the presence of a non-autoimmune type of atrophic gastritis (O’Connell et al. 2018). In subsequent studies, a fourth patient with novel compound heterozygous *WNT2B* variants (a frameshift variant (c.423del and a missense change, c.722 G →  A) was reported with the described missense variant predicted by homology modeling to disrupt post-translational acylation of the protein (Zhang et al. 2021).

The addition of this case also enabled confirmation of the gastrointestinal phenotype and the extension of phenotype to other organs, most notably the eye. Three out of the four reported patients were also found to have significant anterior chamber abnormalities (microcornea, colobomas) establishing *WNT2B* disease as an occulo-intestinal disorder (Zhang et al. 2021). More recent data have also pointed to significant changes in adrenal function with three patients exhibiting hyperreninemic hypoaldosteronism. In terms of management, patients required intensive parenteral nutritional and fluid management as infants. Three of the patients gradually improved with age in terms of enteral tolerance and absorption perhaps suggesting a critical developmental window for the role of *WNT2B* in intestinal function. The specific role and context of *WNT2B* function in different sites within the intestine (stomach, small intestine, and colon) remains to be explored and are the subject of ongoing studies.

### Hoyeraal-Hreidarsson syndrome, TERT, and DKC1

Dyskeratosis congenita (DC) is a multisystem disorder caused by impaired telomere maintenance. The hallmark of DC is the clinical triad of dysplastic nails, lacy reticular pigmentation of the upper back or neck, and oral leukoplakia (Savage and Niewisch 2022). Patients with DC are also at risk for myelodysplasia, pulmonary fibrosis, and neoplasia of many tissues. Hoyeraal–Hreidarsson syndrome (HHS) is a severe variant of DC with the additional features of intrauterine growth restriction, cerebellar hypoplasia, immunodeficiency, and progressive bone marrow failure (Glousker et al. 2015). About one quarter of patients with HHS will also develop a severe enteropathy with chronic diarrhea (Glousker et al. 2015). Diarrhea presents in these patients at an average age of 6.7 years but may present soon after birth in some cases (Jonassaint et al. 2013). Dyskeratosis congenita has a prevalence of approximately 1–9 per 1 million live births, while Hoyeraal–Hreidarsson syndrome represents only a small subset of these cases (Savage and Niewisch 2022).

DC and HHS are diseases caused by the inability of dividing cells to maintain adequate telomere length and thereby avoid telomere crisis and apoptosis (Gramatges and Bertuch 2013). To accomplish telomere elongation, telomerase (encoded by the *TERT* gene) forms a multi-protein complex at the ends of chromosomal DNA (Glousker et al. 2015). A distinct but related complex, the shelterin complex, serves the role of shielding the 3’ telomeric DNA overhang from recognition by DNA repair machinery. As a physical barrier to the 3’ telomeric end, it also regulates the access of telomerase to its site of catalytic activity.

Deficiency in one of the vital components of either the telomerase complex or the shelterin complex results in the dyskeratosis congenita phenotype. Patients with DC are born with very short telomeres, < 1st percentile for their age bracket as measured by flow cytometric techniques (Glousker et al. 2015). Patients with HHS have exceptionally short telomeres even among other cases of classical DC, which may account for their more severe phenotype (Alter et al. 2012). Only a handful of the DC-associated genes are found in association with HHS, and of these only certain variants seem to induce the syndrome.

Biopsy of patients with HHS involving congenital diarrhea demonstrates focally atrophic glandular and epithelial elements with apoptosis in both the colon and duodenum. This is accompanied by a mild mononuclear infiltrate and fibrosis in the lamina propria (Sznajer et al. 2003; Jonassaint et al. 2013). While the exact mechanism by which these patients acquire refractory diarrhea remains unclear, it is generally accepted that disorders of telomere biology result in stem cell failure in highly proliferative tissues (Bertuch 2016; Woo et al. 2016). It is, therefore, suspected that failure of the stem cell niche within the intestinal tract of these patients results in the observed glandular and epithelial atrophy, and in turn the refractory diarrhea.

*DKC1*, encoding the protein dyskerin, was the first gene found in association with DC (specifically the X-linked recessive form of the disease) in 1998 (Knight et al. 1998). Several years later, *TERT*, encoding human telomerase reverse transcriptase, was identified in association with autosomal dominant DC (Armanios et al. 2005). Over the last 20 years, several more genes associated with autosomal dominant and autosomal recessive DC have been discovered, but the only genes with variants also seen in HHS include *TERT, DKC1, RTEL1, TNIF2, ACD,* and *PARN*. *TNIF2* and *ACD* encode components of the shelterin complex while *RTEL1* encodes a DNA helicase that associates with telomerase and *PARN* encodes a poly-A-specific 3’ exoribonuclease (Glousker et al. 2015; Bertuch 2016).

Dyskerin is a nucleolar protein composed of an N-terminal dyskerin-like domain, a central TruB pseudouridine synthetase domain, and a PUA RNA-binding domain. Mutations in dyskerin account for 20–25% of identified cases of dyskeratosis congenita, and many of these represent de novo mutations (Bertuch 2016; Savage and Niewisch 2022). While over 50 disease-causing variants have been identified in *DKC1*, only a quarter of these cause HHS (Glousker et al. 2015; Bertuch 2016). These mutations are overwhelmingly missense mutations, with one identified intronic mutation. Most HHS-associated mutations in dyskerin cluster around the N-terminal dyskerin-like domain (e.g., p.Ile32Thr, p.Thr49Met) or around the PUA domain (e.g., p.Ser304Asp, p.Lys314Arg). Two HHS-associated mutations have also been identified in the TruB domain (p.Ser121Gly, p.Arg158Trp). As of this time, it remains unclear why some mutations manifest as classical DC and some manifest as HHS, with many variants which are HHS-associated in some patients manifesting as DC in others (Glousker et al. 2015). The distinct lack of nonsense and frameshift mutations suggests that some residual dyskerin function is necessary for life, and this is corroborated by the observation that targeted *Dkc1* disruption in mice results in embryonic lethality (He et al. 2002).

Telomerase is an endogenous reverse transcriptase which serves the important role of extending telomeric TTAGGG repeats based on an associated RNA template, encoded by the *hTR* gene and attached via a pseudoknot/template domain (Sekne et al. 2022; Liu et al. 2022). Human telomerase has four domains: the telomerase essential N-terminal (TEN) domain, telomerase RNA-binding (TRB) domain, reverse transcriptase (RT) domain, and C-terminal extension (CTE) domain. As with *DKC1*, disease-associated mutations of *TERT* tend to be missense variants rather than frameshifts or nonsense mutations. Over 50 disease-associated variants of *TERT* have been described in the setting of DC, HHS, idiopathic pulmonary fibrosis, aplastic anemia, Revesz syndrome, and myelodysplastic syndrome (Glousker et al. 2015; Bertuch 2016). Of these, only five variants have been described in association with HHS: p.Pro530Leu, p.Thr567Met, p.Ala880Thr, p.Arg901Trp, and p.Phe1127Leu. The variants p.Ala880Thr and p.Arg901Trp fall within the RT domain and impair telomerase catalytic activity, while p.Pro530Leu and p.Thr567Met fall within the TRB domain. The p.Phe1127Leu mutation falls within the CTE domain and results in a substitution only five residues from the C-terminus of the protein. Of note, this is the only variant of *TERT* which is associated with autosomal dominant inheritance of HHS, although the disease penetrance is incomplete. It has been suggested that *TERT* as well as the related gene *TERC* may display the anticipation phenomenon. This is explained by the theory that a de novo variant may result in a degree of telomere shortening insufficient to cause disease over an individual’s lifetime, but significant enough to cause disease in the next generation if these short telomeres are passed on (Mason and Bessler 2011).

DC and HHS are multisystem disorders. Once either diagnosis is made, patients must be screened for involvement of the gut, lungs, eyes, liver, and bone marrow. The gastrointestinal manifestations can cause significant morbidity and necessitate colectomy and chronic parenteral nutrition in some patients (Jonassaint et al. 2013). Bone marrow failure secondary to disorders of telomerase biology does not respond to immunosuppression as it is not an autoimmune process; however, exogenous androgens have been effective in some patients (Fernández García and Teruya-Feldstein 2014). The only cure for hematologic involvement is allogeneic hematopoietic stem cell transplant (Fioredda et al. 2018).

### EGFR deficiency

The *EGFR* gene encodes the epidermal growth factor receptor (EGFR). The EGF signaling pathway has been well characterized as a vital mitogenic signaling pathway which supports and regulates the stem cell niche in many organs, including the gut (Abud et al. 2021). While most of the literature surrounding this pathway focuses on the important role of EGF gain-of-function in cancer biology, rare cases of congenital EGFR variants with impaired function have also been described. The first of these cases was reported by Campbell et al. in 2014, who described a patient with skin erosions, alopecia, trichomegaly, watery diarrhea, and respiratory difficulties with recurrent pulmonary infections (Campbell et al. 2014). This patient was found to have a homozygous missense mutation in* EGFR* by whole-exome sequencing. Subsequently, Ganetzky et al., Hayashi et al., and Earl et al. reported additional isolated cases and Mazurova et al. identified 18 cases across 16 Roma families (Ganetzky et al. 2015; Hayashi et al. 2018; Mazurova et al. 2020; Earl et al. 2020). Interesting, all patients outside of the Roma cohort had severe diarrhea, but only 3 of the 18 patients in the Roma cohort developed these symptoms, suggesting that genotype may be predictive of gut involvement. Cases of congenital *EGFR* hypofunction have been almost uniformly fatal in the neonatal period, with most cases resulting in death before 6 months of age and only two reported patients living beyond infancy (Mazurova et al. 2020; Earl et al. 2020).

Descriptions of histopathology in patients with *EGFR* hypofunction are scarce in the literature, but the information available paints a picture of mild focal inflammation with generally preserved tissue architecture (Hayashi et al. 2018; Earl et al. 2020). At present, the exact mechanism by which *EGFR* hypofunction results in severe diarrhea remains unclear; however, study of diarrhea in the setting of EGFR tyrosine kinase inhibition for the treatment of malignancy suggests a multifactorial pathology involving atrophic changes, inflammation, and secretory diarrhea (Hirsh et al. 2014; Rugo et al. 2019). Inhibition of Egfr in mice has been shown to decrease pluripotency and self-renewal and to increase differentiation in intestinal stem cells, which is thought to account for the atrophic changes in EGFR inhibition (Yu et al. 2019). Patients with *EGFR* mutations also display shortened telomeres for their respective age bracket, similar to patients with dyskeratosis congenita (Ganetzky et al. 2015).

Among the 23 cases reviewed, only three pathologic variants have been identified. 22 patients demonstrated homozygosity for p.Gly428Asp while only the one case reported by Hayashi et al. demonstrated compound heterozygosity for p.Arg98* and p.Ile365Asn (Hayashi et al. 2018; Mazurova et al. 2020; Earl et al. 2020). The lack of nonsense and frameshift mutations observed in *EGFR* is suggestive that some residual function in this pathway is necessary for life, consistent with the observation that complete *Egfr* knockout leads to pre-implantation death of mouse embryos (Yu et al. 2019). The p.Gly428Asp mutation falls within the second ligand-binding domain of EGFR, and studies indicate decreased affinity of this mutant receptor for immobilized EGF (Siwak et al. 2010; Ganetzky et al. 2015). In contrast, the p.Ile365Asn mutation falls in-between the two ligand-binding domains, and may alter the three-dimensional structure of the receptor in a way that similarly decreases its affinity for EGF.

At current, there are no consensus treatment guidelines for diarrhea in these patients. Many of the patients described required total parenteral nutrition. Given the severity of the diarrhea and electrolyte abnormalities observed in the setting of this intrinsic gut defect, gut transplant may be of utility. Unfortunately, many of these patients present with multiorgan dysfunction which may pose a barrier to successful transplantation.

## Genetic and diagnostic outlook

In the words of Sir Humphrey Davy, “Nothing tends so much to the advancement of knowledge as the application of a new instrument” (Davy 1812). The landscape of human genetics has changed rapidly in the past 20 years and only shows signs of further acceleration with the application of next-generation sequencing (NGS) and CRISPR technologies. Next-generation sequencing has proven to be a revolutionary technology. It may be used to generate a panel of known genes associated with a specific clinical syndrome, utilized for broad sequencing of the entire genome, or targeted at the approximately 22,000 coding genes of the human genome (Choi et al. 2009; Behjati and Tarpey 2013). While the protein-coding genes make up only 1% of the genome, they harbor approximately 85% of known disease-causing variants. As such, whole-exome sequencing has been the modality of choice as a cheaper, easier to analyze alternative to genome sequencing that is less biased than a discrete gene panel. Utilizing whole-exome sequencing of trios, the Deciphering Developmental Disorders Study was able to achieve diagnoses in 40% of over 1000 cases unidentified of congenital disorders (Wright et al. 2018). As a consequence, these data have also led the large-scale discovery of novel developmental diseases and a deeper understanding of developmental biology (Deciphering Developmental Disorders Study, 2015; (Kaplanis et al. 2020). Our own analysis of our CoDE patient cohort has indicated a similar diagnostic success rate.

Whole-exome or -genome sequencing (WES/WGS) is becoming increasingly utilized by clinicians in many specialties, as testing becomes progressively more available and turnaround times for results become shorter. As the biotechnology industry races to advance technologies necessary for widely available genetic sequencing, the cost of sequencing a genome has dropped from 100 million US dollars in 2001 to less than 1000 US dollars in 2021, outstripping the pace of even Moore’s Law for computer processing power (Wetterstrand 2021). This has led to adoption of exome sequencing in some centers for rapid screening of selected newborns (Holm et al. 2018). Given the current cost and turnaround times for WES/WGS, there is ongoing consideration of expansion of universal newborn screening to include more rare diseases including certain CoDE disorders where early diagnosis can significantly impact management or treatment. Although the costs of sequencing continue to drop, the availability of genetic sequencing remains restricted to high-income countries, or specialist tertiary hospitals and academic centers. Extending access to communities more broadly should be an important goal moving forward. In addition to access, the limitations to sequencing are changing from economic and practical to ethical and sociological: How do we ensure proper informed consent for these cases? How do we balance the wellbeing of the infant and their family, the right of family members to the privacy of their own genomes, and the benefit of this knowledge to society at large (Remec et al. 2021)? Genetic studies involving ethnic groups face unique concerns regarding equity, such as those of the Navajo Nation, who placed a moratorium on genetic research studies within their jurisdiction in 2002 over concerns for data mistreatment and potential for injustice (Claw et al. 2021).

The oldest of the disorders described in this paper were originally identified as functional protein deficiencies using basic biochemical methods and time-consuming mapping onto respective chromosomal locations. The patients described in these articles were diagnosed only due to the availability of research expertise and funding. Now, patients with new gene mutations are being identified in routine clinical practice at high-volume centers with the aid of next-generation sequencing. This has led to a rapid increase in the array of known disease-causing variants, due in part to the contributions from molecular geneticists and clinicians analyzing exome sequences both within and outside of a research context (Rockowitz et al. 2020). As the cost of sequencing continues to drop and this technology becomes further integrated into clinical practice, the rate of discovery may yet increase for CoDE disorders. The new challenge for the field will be putting this torrent of genetic data into the context of intestinal physiology and deciphering the underlying cell biological mechanisms involved.

## Therapeutic outlook

As the genetic basis of CoDE disorders becomes increasingly more defined and precise, the next major challenge in the field relates to the development of therapies for these rare disorders. A critical first step in this process is understanding the tissue pathophysiology, genotype–phenotype correlations and cell biology related to the epithelial proteins involved. Studies on the natural history of individual disorders and in-depth analysis of tissue histology are needed including the use of new spatial methods such as multiplex immunofluorescence (Hickey et al. 2022), spatial transcriptomics (Moffitt et al. 2016) and machine-learning based analysis (Syed et al. 2019). An important effort as part of these analyses are resources for the research community such as patient registries, and systematic characterization of intestinal changes such as tissue and cellular atlases for CoDE disorders. The formation of national and international consortia to study these diseases such as the Pediatric Congenital Diarrhea Consortium (PediCoDE; www.pedicode.org) has now brought together many of these community resources and an important next step for the field is to leverage these tools for therapeutic benefit.

One major method for both understanding biology and the development of therapies is the use of patient-derived intestinal primary cells or enteroids in both three- and two-dimensional models. Since CoDE disorders involve epithelial dysfunction, enteroids represent a uniquely powerful model for these disorders and accurately reflect the cellular changes seen in the native intestine. Recent studies in a number of disorders have highlighted their use in understanding mechanisms of epithelial dysfunction (van Rijn et al. 2019; van Vugt et al. 2021; Duclaux-Loras et al. 2022). Recent studies have also shown how enteroids may be leveraged to accelerate therapy development by acting as a patient-specific method for therapy validation (Jardine et al. 2020). In addition to validation, resource generation and biobanking of patient samples may soon enable medium throughput primary screening for potential therapies.

The identification of the genetic basis of disorders has also presented the promise of direct gene-targeted therapy based on specific variants. Antisense oligonucleotides (ASOs) represent a particularly attractive option for this application, as they can be designed upon the basis of the coding sequence alone. ASOs are modified nucleotide sequences which are capable of changing protein expression by interacting with immature mRNA to either promote mRNA degradation or to affect mRNA splicing (Oberemok et al. 2018). These therapeutics have found success in the treatment of neurodevelopmental and neurodegenerative disorders, such as nusinersen for spinal muscular atrophy and inotersen for hereditary transthyretin amyloidosis (Finkel et al. 2017; Benson et al. 2018). In an example of this type of precision therapy, Kim et al. identified a rare case of Batten’s disease in a young patient, determined the causative variant by whole-exome sequencing, and used this information to develop a splice-altering OSA (milasen) which was then used to treat this patient in a N-of-1 clinical study to significant effect (Kim et al. 2019). ASOs are, however, primarily useful in cases with gain-of-function variants or variants in which an existing, unaffected splice variant may be phenocopied by exon skipping. Another major hurdle, specific to their use in CoDE disorders, is the delivery and retention of intact ASOs within the GI tract. The use of ASOs and other gene therapies for intestinal genetic disorders, while holding tremendous promise, are therefore not quite ready yet for clinical application.

## Summary

Monogenic intestinal epithelial disorders or CoDE disorders are a unique group of genetic intestinal disorders characterized by primary epithelial defects that lead to altered fluid and electrolyte transport, barrier disruption, and defects in tissue development and regeneration (Tables 1 and 2). Many CoDEs exhibit little to no chronic mucosal inflammation although several disorders are characterized by varying levels of mucosal inflammation that is largely secondary to their primary epithelial dysfunction. This is distinct from genetic disorders that can be characterized as very early-onset IBD or primary immunodeficiency with intestinal involvement that primarily affect immune cell function. The genetic basis of CoDE disorders is now increasingly well defined and information on specific disorders as outlined in this review continues to grow. The next frontier in the field is to translate these new genetic and biological insights into interventions that can significantly change the lives of CoDE patients.

**Table 1:** Genetics of CoDE disorders

Disorder	Name	Inheritance	Incidence (fraction of live births)	High prevalence populations	Protein function
Congenital chloride diarrhea (MIM #214700)	*SLC26A3*	AR	1 in 40,000 (Saudi Arabia, Kuwait, Finland), 1 in 200,000 (Poland)	Arab, Finnish, Polish	Cl^–^/HCO_3_^–^ exchanger
Congenital sodium diarrhea (MIM #616868) (MIM #614616)	*SLC9A3*	AR	Unknown	Finnish	Na^+^/H^+^ exchanger (NHE3)
	*GUCY2C*	AD	Unknown	Unknown	Guanylate cyclase
Lysinuric Protein Intolerance (MIM #222700)	*SLC7A7*	AR	1 in 60,000 (Finland, France)	Finnish, French, Japanese	Amino acid transporter
Glucose-galactose malabsorption (MIM #606824)	*SLC5A1*	AR	Unknown	Arab, Amish	Na^+^–glucose cotransporter
Primary bile acid diarrhea (MIM #613291)	*SLC10A2*	AR	Unknown	Swedish	Apical bile acid transporter
	*SLC51B*	AR	Unknown	Unknown	Basolateral bile acid transporter
Acrodermatitis enteropathica (MIM #201100)	*SLC39A4*	AR	1 in 500,000 (Netherlands)	None	Zinc transporter
Congenital lactase deficiency (MIM #223000)	*LCT*	AR	1 in 60,000 (Finland)	Finnish	Disaccharidase
Sucrase-isomaltase deficiency (MIM #222900)	*SI*	AR	1 in 5,000 (Europe), 1 in 33 (Native Canadian, Alaskan, and Greenlandic)	Canadian, Alaskan, and Greenlandic Inuit	Disaccharidase
Trehalase deficiency (MIM #612119)	*TREH*	AR/AD	1 in 100,000 (Worldwide)	Finnish and Greenlandic Inuit	Disaccharidase
DGAT1 deficiency (MIM #615,863)	*DGAT1*	AR	< 1 in 1,000,000 (Worldwide)	Ashkenazi Jewish	Triglyceride synthesis
Abetalipoproteinemia (MIM #200100)	*MTP*	AR	1 in 1,000,000 (Worldwide), 1 in 70,000 (Ashkenazi Jewish)	Ashkenazi Jewish	Microsomal triglyceride transfer protein
Hypobetalipoproteinemia (MIM #615558)	*APOB*	AR/AD	Unknown	None	Lipid absorption
Chylomicron retention disease (MIM #246700)	*SAR1B*	AR	< 1 in 1,000,000 (Worldwide)	French Canadian, North African	Intracellular chylomicron trafficking
Enterokinase deficiency (MIM #226200)	*TMPRSS15*	AR	Unknown	None	Pro-enterokinase
EAGLES syndrome (MIM #606358)	*AGR2*	AR	Unknown	Syrian	Protein disulfide isomerase
WNT2B deficiency (MIM #618168)	*WNT2B*	AR	Unknown	Unknown	Growth factor
Dyskeratosis congenita, Hoyeraal–Hreidarsson syndrome (MIM #613989)	*TERT*	AR/AD	< 1 in 1,000,000 (Europe)	Unknown	Maintenance of telomeres
	*DKC1*	XR			
	*RTEL1*	AR			
	*PARN*	AR			
	*TINF2*	AD			Shelterin complex
	*ACD*	AR			
EGFR deficiency (MIM #616069)	*EGFR*	AR	Unknown	Unknown	Growth factor receptor
Microvillus inclusion disease (MIM #251850)	*MYO5B*	AR	< 1 in 1,000,000 (Europe)	Navajo, Arab	Cellular trafficking, polarity, and signaling
	*STX3*	AR		Unknown	Syntaxin
	*UNC45A*	AR		Unknown	Chaperone
Tufting enteropathy (MIM #613217)	*EPCAM*	AR	1 in 100,000 (Western Europe)	Arab, Mexican	Cell–cell adhesion and signaling
Syndromic Na^+^ diarrhea /tufting enteropathy (MIM #270420)	*SPINT2*	AR	1 in 500,000 (Western Europe)	Unknown	Serine protease inhibitor
Trichohepatoenteric syndrome 1 (THES1) (MIM #222470)	*TTC37*	AR	< 1 in 1,000,000 (Worldwide)	Pakistani, French, North African	Cell polarity and signaling
Trichohepatoenteric syndrome 2 (THES2) (MIM #614602)	*SKIV2L*	AR/AD	< 1 in 1,000,000 (Worldwide)	Turkish, French	Helicase
Familial hemophagocytic lymphohistiocytosis 5 (MIM #613101)	*STXBP2*	AR	1 in 1,000,000 (Worldwide)	Turkish, Kurdish, Pakistani	Syntaxin-binding protein
TTC7A deficiency (MIM #243150)	*TTC7A*	AR	Unknown	French Canadian	Protein transport and trafficking
Congenital Disease of Glycosylation type 1b (MIM #602579)	*MPI*	AR	< 1 in 1,000,000 (Worldwide)	Unknown	Mannose phosphate isomerase
Congenital Disease of Glycosylation type 1 h (MIM #608104)	*ALG8*	AR	< 1 in 1,000,000 (Worldwide)	Unknown	Glucosyl-transferase
MEDNIK syndrome (MIM #609313)	*AP1S1*	AR	< 1 in 1,000,000 (Worldwide)	French Canadian	Clathrin coat assembly
Enteric anendocrinosis (MIM #610370)	*NEUROG3*	AR	Unknown	Unknown	Transcription factor–cell fate
X-linked lissencephaly (MIM #300215)	*ARX*	XR	Unknown	Unknown	Homeodomain transcription factors
Enteric dysendocrinosis (MIM #600955)	*PCSK1*	AR	Unknown	Unknown	A hormonal endopeptidase
Mitchell-Riley syndrome (MIM #615710)	*RFX6*	AR	Unknown	Unknown	Transcription factor–cell fate
Intractable Malabsorptive Diarrhea of Infancy syndrome (MIM #618662)	*PERCC1*	AR	Unknown	Iraqi-Jewish	Enteroendocrine cell differentiation

*AR* autosomal recessive, *AD* autosomal dominant, *XR* X-linked recessive

**Table 2:** Intestinal phenotype and clinical characteristics of CoDE disorders

Gene name	Diarrhea type	Biopsy features	Enteral autonomy	Other intestinal characteristics	Extra-intestinal features
Epithelial transport					
*SLC26A3*	Severe watery, ETRD*	Normal	Yes	High fecal Cl^−^ (> 90 mM)	Polyhydramnios; hypochloremic hypokalemic metabolic alkalosis
*SLC9A3*	Severe watery, ETRD	Normal	Yes	High fecal Na^+^ content (typically > 140 mM)	Polyhydramnios; metabolic acidosis
*GUCY2C*	Severe watery, ETRD	Normal	Yes	High fecal Na^+^ content (typically > 140 mM)	Polyhydramnios; metabolic acidosis
*SLC5A1*	Severe watery, diet-induced	Normal	Yes	Diarrhea resolves with fasting or with a glucose and galactose-free diet; severe dehydration	Metabolic acidosis
* SLC10A2*	Watery/ fatty, ETRD and diet-induced	Normal	Yes	Excessive colonic Cl^−^ secretion; also fat malabsorption, due to lack of bile acid reuptake and insufficient bile acids in the intestinal lumen. In this condition, diarrhea worsens with feeds, unlike other forms of ETRD.*	Fat-soluble vitamin deficiency; cholestatic liver disease
*SLC51B*	As above for *SLC10A2*				
*SLC39A4*	Watery	Normal	Yes	Variable severity diarrhea	Erythematous vesiculobullous acrodermatitis (typically on cheeks and buttocks), alopecia, ophthalmic disorders, low serum zinc
* SLC7A7*	Severe watery, diet-induced	Normal, sometimes villous atrophy	Yes	Diarrhea and coma following a protein-rich meal. Diarrhea often resolves with protein restriction	Metabolic acidosis; orotic aciduria
Epithelial enzymes and metabolism					
*LCT*	Watery, diet-induced	Normal	Yes	Diarrhea resolves with fasting or a lactose-free diet. Must be distinguished from secondary lactose intolerance, which is induced by mucosal injury (after an enteric infection) and is transient	
* SI*	Watery, diet-induced	Normal	Yes	Diarrhea resolves with fasting or a carbohydrate-free diet (no sucrose, maltose, or starch); IBS	Failure to thrive
* TREH*	Watery	Normal	Yes	Diarrhea associated with a dose-dependent consumption of a trehalose-containing diet (e.g., mushrooms or foods processed with bakers’ yeast). Young infants are asymptomatic because trehalose is not in typical infant formula or breast milk	
* PRSS7 /TMPRSS15*	Watery, diet-induced	Normal	Yes	Diarrhea that improves on an amino acid-based diet	Failure to thrive, low serum protein, and diffuse edema starting in the first weeks of life
* DGAT1*	Watery, diet-induced	Abnormal villi; positive PAS stain	Yes	Diarrhea induced by enteral lipids, ETRD; severe protein-losing enteropathy, emesis	Hypoalbuminemia, growth failure
* PLVAP*	Watery, diet-induced	Normal	Yes	Severe protein-losing enteropathy, hematochezia	Hypoalbuminemia, hypertriglyceridemia possible, early-onset anasarca, hypogammaglobulinemia; facial abnormalities, cardiac and renal anomalies possible
*MTTP*	Fatty, diet-induced	Fat-laden enterocytes	Yes	Normal fecal elastase	Low LDL
*APOB*	Fatty, diet-induced	Fat-laden enterocytes	Yes	Normal fecal elastase	Low LDL
*SAR1B*	Fatty, diet-induced	Fat-laden enterocytes	Yes	Normal fecal elastase	Low LDL, deficiency of fat-soluble vitamins, selective absence of chylomicrons
*AGR2*	Watery	Goblet cells lacking mucin, regenerative crypts, apoptotic bodies and mitotic figures	Yes	Infantile-onset Inflammatory bowel disease-like clinical picture	Recurrent lower respiratory tract infections, bronchiectasis, cardiac anomalies, hepatosplenomegaly
Epithelial trafficking and polarity					
* MYO5B*	Watery, ETRD	Villous atrophy, microvillus inclusions on EM; positive CD10/villin stain	No (possible, but rare)	Diarrhea that improves but does not resolve with fasting	Familial intrahepatic cholestasis
* STX3*	Severe watery ETRD	Villous atrophy, abnormal microvilli	Variable	Diarrhea that improves but does not resolve with fasting	Congenital retinopathy and intellectual disability possible
*UNC45A*	Severe watery ETRD	Villous atrophy, abnormal microvilli	No	Diarrhea that improves but does not resolve with fasting	Cholestasis, impaired hearing, bone fragility
* EPCAM*	Severe watery ETRD	Villous atrophy and focal epithelial tufts in small and large bowel; positive MOC31 stain	Variable	Severe watery diarrhea that persists with fasting	
*SPINT2*	Severe watery ETRD	Tufting enteropathy-like features	No	Rarely present with intestinal atresia	Choanal atresia
*TTC37*	Watery/bloody	Villous atrophy, mononuclear infiltrates	No		Liver disease, immune defects, facial dysmorphism, abnormal hair
* SKIV2L*	Watery/bloody	Villous atrophy, mononuclear infiltrates	No		Liver disease, immune defects, facial dysmorphism, abnormal hair
*STXBP2*	Diet-induced	Short apical microvilli	No	Chronic diarrhea, which might not resolve after bone marrow transplant	Hemophagocytic lymphohistiocytosis (excessive immune activation, hepatitis, cytopenias)
* TTC7A*	ETRD	Enterocolitis, villous atrophy, and chronic inflammation	Variable	Intestinal atresia	Underlying SCID
*MPI*	Watery	Villous atrophy, mild lymphangiectasias	Yes	Protein-losing enteropathy, accompanied by cyclic or episodic vomiting	Hepatic fibrosis, thrombophilia with low protein S, C, and antithrombin III. Improves with exogenous mannose
*ALG8*	Watery	Villous atrophy, mild lymphangiectasias	No	Protein-losing enteropathy, accompanied by cyclic or episodic vomiting	Hepatic fibrosis, skeletal dysplasia, proteinuria, thrombophilia with low antithrombin III. Does not improve with exogenous mannose
*AP1S1*	Watery, ETRD	Unknown	Variable (some patients able to be weaned off with time)	Severe early-onset salt-wasting diarrhea	Developmental delay, deafness, ichthyosis with keratodermia, liver fibrosis, hypocupremia
Enteroendocrine cell function					
*NEUROG3*	Watery, diet-induced	Normal villous architecture; selective loss of EEC’s (chromogranin or synaptophysin stains)	Yes	Generalized malabsorption	Later onset of insulin-dependent diabetes (without antibodies)
*ARX*	Diet-induced	Normal villous architecture; selective reduction of certain EEC’s	Yes	Generalized malabsorption	Lissencephaly (smooth cerebral cortex) and severe neurologic abnormalities, seizures
*PCSK1*	Severe, diet-induced	Normal villous architecture	Yes	Generalized malabsorption	Multiple systemic endocrinopathies (adrenal insufficiency, hypothyroidism, diabetes insipidus); elevated pro-insulin, metabolic acidosis
* RFX6*	Watery	Normal villous architecture and enteroendocrine cells	Yes	Generalized malabsorption, intestinal atresia, malrotation, intrinsic and extrinsic biliary duct abnormalities	Neonatal diabetes
*PERCC1*	Severe watery secretory diarrhea	Range from normal to partial villous atrophy	Variable (most patients are able to be weaned off PN after the first decade of life)	Generalized malabsorption	
Stem cell function					
*RTEL1*	Watery/ bloody	Crypt apoptosis, increased intraepithelial lymphocytes, villous atrophy, and expanded lamina propria	Variable (some patients may require life-long parenteral nutrition and colectomy)	Can present with esophageal stenosis and enterocolitis; celiac enteropathy diagnosis possible	Dysplastic nails, lacy reticular pigmentation of the upper back or neck, oral leukoplakia, myelodysplasia, mucosal fragility, pulmonary or liver fibrosis, developmental delay, microcephaly
*TINF2*					
*ACD*					
*PARN*					
*TERT*					
* DKC1*					
*WNT2B*	Severe watery diarrhea	Effacement of crypt architecture, crypt dropout, chronic inflammatory infiltrate in the lamina propria	No	Intestinal failure	Ocular dysgenesis
*EGFR*	Watery	Focal chronic inflammatory infiltrate	Variable		Polyhydramnios; fragile, ichthyotic skin; recurrent respiratory infections

*CoDEs* congenital diarrheas and enteropathies, *ETRD* electrolyte transport-related diarrhea, *IBS* irritable bowel syndrome, *PAS* periodic acid-Schiff, *LDL* lowdensity lipoprotein, *EM* electron microscopy, *SCID* severe combined immunodeficiency, *EEC’s* enteroendocrine cells, *PN* parenteral nutrition

Original table modified for this publication. From: Thiagarajah et al. (2018). Table used with the permission of Elsevier Inc. All rights reserved.

## Supplementary Information

Below is the link to the electronic supplementary material.

Supplementary file 1 (DOCX 22 KB)

## Data Availability

Data sharing not applicable to this article as no datasets were generated or analysed during the current study.

## References

[CR1] Abud HE, Chan WH, Jardé T (2021). Source and impact of the EGF family of ligands on intestinal stem cells. Front Cell Dev Biol.

[CR2] Aerts L, Terry NA, Sainath NN (2021). Novel homozygous inactivating mutation in the PCSK1 gene in an infant with congenital malabsorptive diarrhea. Genes.

[CR3] Aftab S, Semenec L, Chu JS-C, Chen N (2008). Identification and characterization of novel human tissue-specific RFX transcription factors. BMC Evol Biol.

[CR4] Aichbichler BW, Zerr CH, Santa Ana CA (1997). Proton-pump inhibition of gastric chloride secretion in congenital chloridorrhea. N Engl J Med.

[CR5] Albokhari D, Ng BG, Guberinic A (2022). ALG8-CDG: Molecular and phenotypic expansion suggests clinical management guidelines. J Inherit Metab Dis.

[CR6] Aldrian D, Vogel GF, Frey TK (2021). Congenital diarrhea and cholestatic liver disease: phenotypic spectrum associated with MYO5B mutations. J Clin Med.

[CR7] Alsaleem BMR, Ahmed ABM, Fageeh MA (2017). Microvillus inclusion disease variant in an infant with intractable diarrhea. CRG.

[CR8] Al-Shaibi AA, Abdel-Motal UM, Hubrack SZ (2021). Human AGR2 deficiency causes mucus barrier dysfunction and infantile inflammatory bowel disease. Cell Mol Gastroenterol Hepatol.

[CR9] Alter BP, Rosenberg PS, Giri N (2012). Telomere length is associated with disease severity and declines with age in dyskeratosis congenita. Haematologica.

[CR10] Angeli S, Lin X, Liu XZ (2012). Genetics of hearing and deafness. Anat Rec.

[CR11] Anguita-Ruiz A, Aguilera CM, Gil Á (2020). Genetics of lactose intolerance: an updated review and online interactive world maps of phenotype and genotype frequencies. Nutrients.

[CR13] Armanios M, Chen J-L, Chang Y-PC (2005). Haploinsufficiency of telomerase reverse transcriptase leads to anticipation in autosomal dominant dyskeratosis congenita. Proc Natl Acad Sci USA.

[CR14] Asano K, Matsushita T, Umeno J (2009). A genome-wide association study identifies three new susceptibility loci for ulcerative colitis in the Japanese population. Nat Genet.

[CR15] Auclair N, Sané AT, Ahmarani L (2021). Sar1b mutant mice recapitulate gastrointestinal abnormalities associated with chylomicron retention disease. J Lipid Res.

[CR16] Avery GB, Villavicencio O, Lilly JR, Randolph JG (1968). Intractable diarrhea in early infancy. Pediatrics.

[CR17] Avitzur Y, Guo C, Mastropaolo LA (2014). Mutations in tetratricopeptide repeat domain 7A result in a severe form of very early onset inflammatory bowel disease. Gastroenterology.

[CR18] Ayoub C, Azar Y, Abou-Khalil Y (2021). Identification of a variant in APOB gene as a major cause of hypobetalipoproteinemia in lebanese families. Metabolites.

[CR19] Barral JM, Bauer CC, Ortiz I, Epstein HF (1998). Unc-45 mutations in *Caenorhabditis elegans* implicate a CRO1/She4p-like domain in myosin assembly. J Cell Biol.

[CR20] Baxter SK, Walsh T, Casadei S (2022). Molecular diagnosis of childhood immune dysregulation, polyendocrinopathy, and enteropathy, and implications for clinical management. J Allergy Clin Immunol.

[CR21] Behjati S, Tarpey PS (2013). What is next generation sequencing?. Arch Dis Child Educ Pract Ed.

[CR22] Benavides N, Spessott WA, Sanmillan ML (2020). STXBP2-R190C variant in a patient with neonatal hemophagocytic lymphohistiocytosis (HLH) and G6PD deficiency reveals a critical role of STXBP2 domain 2 on granule exocytosis. Front Immunol.

[CR23] Benn M, Stene MCA, Nordestgaard BG (2008). Common and rare alleles in apolipoprotein B contribute to plasma levels of low-density lipoprotein cholesterol in the general population. J Clin Endocrinol Metab.

[CR24] Benson MD, Waddington-Cruz M, Berk JL (2018). Inotersen treatment for patients with hereditary transthyretin amyloidosis. N Engl J Med.

[CR25] Bertoli-Avella A, Hotakainen R, Al Shehhi M (2021). A disorder clinically resembling cystic fibrosis caused by biallelic variants in the AGR2 gene. J Med Genet.

[CR26] Bertuch AA (2016). The molecular genetics of the telomere biology disorders. RNA Biol.

[CR27] Bienvenu T, Poirier K, Friocourt G (2002). ARX, a novel Prd-class-homeobox gene highly expressed in the telencephalon, is mutated in X-linked mental retardation. Hum Mol Genet.

[CR28] Bigorgne AE, Farin HF, Lemoine R (2014). TTC7A mutations disrupt intestinal epithelial apicobasal polarity. J Clin Invest.

[CR29] Biterova EI, Isupov MN, Keegan RM (2019). The crystal structure of human microsomal triglyceride transfer protein. Proc Natl Acad Sci USA.

[CR30] Bogdanic E, Müller T, Heinz-Erian P (2022). Further delineation of SLC9A3-related congenital sodium diarrhea. Mol Genet Genomic Med.

[CR31] Bourgeois P, Esteve C, Chaix C (2018). Tricho-Hepato-Enteric Syndrome mutation update: mutations spectrum of TTC37 and SKIV2L, clinical analysis and future prospects. Hum Mutat.

[CR32] Braamskamp MJAM, Dolman KM, Tabbers MM (2010). Clinical practice. Protein-losing enteropathy in children. Eur J Pediatr.

[CR33] Bruns JB, Carattino MD, Sheng S (2007). Epithelial Na+ channels are fully activated by furin- and prostasin-dependent release of an inhibitory peptide from the gamma-subunit. J Biol Chem.

[CR34] BugdaGwilt K, Thiagarajah JR (2022). Membrane lipids in epithelial polarity: sorting out the PIPs. Front Cell Dev Biol.

[CR35] Burnett JR, Hooper AJ, Hegele RA (2021). APOB-related familial hypobetalipoproteinemia.

[CR36] Byeon MK, Westerman MA, Maroulakou IG (1996). The down-regulated in adenoma (DRA) gene encodes an intestine-specific membrane glycoprotein. Oncogene.

[CR37] Cai C, Chen Y, Chen X, Ji F (2020). Tufting enteropathy: a review of clinical and histological presentation, etiology, management, and outcome. Gastroenterol Res Pract.

[CR38] Campbell P, Morton PE, Takeichi T (2014). Epithelial inflammation resulting from an inherited loss-of-function mutation in EGFR. J Invest Dermatol.

[CR39] Canna SW, Marsh RA (2020). Pediatric hemophagocytic lymphohistiocytosis. Blood.

[CR40] Cases S, Smith SJ, Zheng Y-W (1998). Identification of a gene encoding an acyl CoA:diacylglycerol acyltransferase, a key enzyme in triacylglycerol synthesis. Proc Natl Acad Sci USA.

[CR41] Čechová A, Altassan R, Borgel D (2020). Consensus guideline for the diagnosis and management of mannose phosphate isomerase-congenital disorder of glycosylation. J Inherit Metab Dis.

[CR42] Chan AP, Namjoshi SS, Jardack PM (2021). Long-term dietary changes in subjects with glucose galactose malabsorption secondary to biallelic mutations of SLC5A1. Dig Dis Sci.

[CR43] Chantret I, Dancourt J, Dupré T (2003). A deficiency in dolichyl-P-glucose:Glc1Man9GlcNAc2-PP-dolichyl alpha3-glucosyltransferase defines a new subtype of congenital disorders of glycosylation. J Biol Chem.

[CR44] Charcosset M, Sassolas A, Peretti N (2008). Anderson or chylomicron retention disease: molecular impact of five mutations in the SAR1B gene on the structure and the functionality of Sar1b protein. Mol Genet Metab.

[CR45] Chen R, Giliani S, Lanzi G (2013). Whole-exome sequencing identifies tetratricopeptide repeat domain 7A (TTC7A) mutations for combined immunodeficiency with intestinal atresias. J Allergy Clin Immunol.

[CR46] Chograni M, Alkuraya FS, Ourteni I (2015). Autosomal recessive congenital cataract, intellectual disability phenotype linked to STX3 in a consanguineous Tunisian family. Clin Genet.

[CR47] Choi M, Scholl UI, Ji W (2009). Genetic diagnosis by whole exome capture and massively parallel DNA sequencing. Proc Natl Acad Sci USA.

[CR48] Choi W, Yeruva S, Turner JR (2017). Contributions of intestinal epithelial barriers to health and disease. Exp Cell Res.

[CR49] Claw KG, Dundas N, Parrish MS (2021). Perspectives on genetic research: results from a survey of Navajo community members. Front Genet.

[CR50] Cohen MB, Guarino A, Shukla R, Giannella RA (1988). Age-related differences in receptors for *Escherichia coli* heat-stable enterotoxin in the small and large intestine of children. Gastroenterology.

[CR51] Collombat P, Mansouri A, Hecksher-Sorensen J (2003). Opposing actions of Arx and Pax4 in endocrine pancreas development. Genes Dev.

[CR52] Culbreath K, Keefe G, Nes E (2022). Intestinal atresias and intestinal failure in patients with TTC7A mutations. J Pediatr Surg Case Rep.

[CR53] Cutz E, Rhoads JM, Drumm B (1989). Microvillus inclusion disease: an inherited defect of brush-border assembly and differentiation. N Engl J Med.

[CR54] Dannheim K, Ouahed J, Field M (2022). Pediatric gastrointestinal histopathology in patients with tetratricopeptide repeat domain 7A (TTC7A) germline mutations: a rare condition leading to multiple intestinal atresias, severe combined immunodeficiency, and congenital enteropathy. Am J Surg Pathol.

[CR55] Darrow DC (1945). Congenital alkalosis with diarrhea. J Pediatr.

[CR56] Das B, Sivagnanam M (2020). Congenital tufting enteropathy: biology, pathogenesis and mechanisms. J Clin Med.

[CR57] Das B, Okamoto K, Rabalais J (2021). Aberrant epithelial differentiation contributes to pathogenesis in a murine model of congenital tufting enteropathy. Cell Mol Gastroenterol Hepatol.

[CR58] Davidson GP, Cutz E, Hamilton JR, Gall DG (1978). Familial enteropathy: a syndrome of protracted diarrhea from birth, failure to thrive, and hypoplastic villus atrophy. Gastroenterology.

[CR59] Davy SH (1812) Elements of chemical philosophy. Bradford and Inskeep

[CR60] de Koning TJ, Dorland L, van Diggelen OP (1998). A novel disorder of N-glycosylation due to phosphomannose isomerase deficiency. Biochem Biophys Res Commun.

[CR61] Deng Z, Zhao Y, Ma Z (2021). Pathophysiological role of ion channels and transporters in gastrointestinal mucosal diseases. Cell Mol Life Sci.

[CR62] Dhekne HS, Pylypenko O, Overeem AW (2018). MYO5B, STX3, and STXBP2 mutations reveal a common disease mechanism that unifies a subset of congenital diarrheal disorders: a mutation update. Hum Mutat.

[CR63] Di Meglio L, Grimaldi G, Esposito F (2022). Step-up approach for sodium butyrate treatment in children with congenital chloride diarrhea. Front Pediatr.

[CR64] Diekmann L, Pfeiffer K, Naim HY (2015). Congenital lactose intolerance is triggered by severe mutations on both alleles of the lactase gene. BMC Gastroenterol.

[CR65] Dimitrov G, Bamberger S, Navard C (2019). Congenital Sodium Diarrhea by mutation of the SLC9A3 gene. Eur J Med Genet.

[CR66] Doya LJ, Mohammad L, Omran R (2021). Chylomicron retention disease caused by a new pathogenic variant in sar1b protein: a rare case report from Syria. BMC Pediatr.

[CR67] Duclaux-Loras R, Lebreton C, Berthelet J (2022). UNC45A deficiency causes microvillus inclusion disease-like phenotype by impairing myosin VB-dependent apical trafficking. J Clin Invest.

[CR68] Duncan MC (2022). New directions for the clathrin adaptor AP-1 in cell biology and human disease. Curr Opin Cell Biol.

[CR69] Earl BR, Szybowska M, Marwaha A (2020). Epidermal growth factor receptor deficiency: expanding the phenotype beyond infancy. J Dermatol.

[CR70] Eckard SC, Rice GI, Fabre A (2014). The SKIV2L RNA exosome limits activation of the RIG-I-like receptors. Nat Immunol.

[CR71] Eklund EA, Freeze HH (2006). The congenital disorders of glycosylation: a multifaceted group of syndromes. NeuroRx.

[CR72] Eldredge JA, Couper MR, Barnett CP (2021). New pathogenic mutations associated with diacylglycerol O-acyltransferase 1 deficiency. J Pediatr.

[CR73] Elkadri AA (2020). Congenital diarrheal syndromes. Clin Perinatol.

[CR74] Engevik AC, Kaji I, Engevik MA (2018). Loss of MYO5B leads to reductions in Na+ absorption with maintenance of CFTR-dependent Cl-secretion in enterocytes. Gastroenterology.

[CR75] Erickson RP, Larson-Thomé K, Valenzuela RK (2008). Navajo microvillous inclusion disease is due to a mutation in MYO5B. Am J Med Genet A.

[CR76] Esteve C, Francescatto L, Tan PL (2018). Loss-of-function mutations in UNC45A cause a syndrome associating cholestasis, diarrhea, impaired hearing, and bone fragility. Am J Hum Genet.

[CR77] Evanson JM, Stanbury SW (1965). Congenital chloridorrhoea or so-called congenital alkalosis with diarrhoea. Gut.

[CR78] Fabre A, Bourgeois P, Chaix C et al (1993) Trichohepatoenteric syndrome. In: Adam MP, Mirzaa GM, Pagon RA et al (eds) GeneReviews®. University of Washington, Seattle, Seattle (WA) 29334452

[CR79] Fabre A, Martinez-Vinson C, Roquelaure B (2011). Novel mutations in TTC37 associated with tricho-hepato-enteric syndrome. Hum Mutat.

[CR80] Fabre A, Charroux B, Martinez-Vinson C (2012). SKIV2L mutations cause syndromic diarrhea, or trichohepatoenteric syndrome. Am J Hum Genet.

[CR81] Fabre A, Petit L-M, Hansen LF (2018). A new mutation in the C-terminal end of TTC37 leading to a mild form of syndromic diarrhea/tricho-hepato-enteric syndrome in seven patients from two families. Am J Med Genet A.

[CR82] Farooqi IS, Volders K, Stanhope R (2007). Hyperphagia and early-onset obesity due to a novel homozygous missense mutation in prohormone convertase 1/3. J Clin Endocrinol Metab.

[CR83] Fernández García MS, Teruya-Feldstein J (2014). The diagnosis and treatment of dyskeratosis congenita: a review. J Blood Med.

[CR84] Ferreira H, Ramos RN, Quan CF (2018). Chylomicron retention disease: a description of a new mutation in a very rare disease. Pediatr Gastroenterol Hepatol Nutr.

[CR85] Fessart D, Domblides C, Avril T (2016). Secretion of protein disulphide isomerase AGR2 confers tumorigenic properties. Elife.

[CR86] Finkel RS, Mercuri E, Darras BT (2017). Nusinersen versus sham control in infantile-onset spinal muscular atrophy. N Engl J Med.

[CR87] Fioredda F, Iacobelli S, Korthof ET (2018). Outcome of haematopoietic stem cell transplantation in dyskeratosis congenita. Br J Haematol.

[CR88] Fiskerstrand T, Arshad N, Haukanes BI (2012). Familial diarrhea syndrome caused by an activating *GUCY2C* mutation. N Engl J Med.

[CR89] Forte LR, London RM, Krause WJ, Freeman RH (2000). Mechanisms of guanylin action via cyclic GMP in the kidney. Annu Rev Physiol.

[CR90] Friis S, Uzzun Sales K, Godiksen S (2013). A matriptase-prostasin reciprocal zymogen activation complex with unique features. J Biol Chem.

[CR91] Gamble JL, Fahey KR, Appleton J, MacLachlan E (1945). Congenital alkalosis with diarrhea. J Pediatr.

[CR92] Ganetzky R, Finn E, Bagchi A (2015). EGFR mutations cause a lethal syndrome of epithelial dysfunction with progeroid features. Mol Genet Genomic Med.

[CR93] Gehart H, Clevers H (2019). Tales from the crypt: new insights into intestinal stem cells. Nat Rev Gastroenterol Hepatol.

[CR94] Georges A, Bonneau J, Bonnefont-Rousselot D (2011). Molecular analysis and intestinal expression of SAR1 genes and proteins in Anderson’s disease (Chylomicron retention disease). Orphanet J Rare Dis.

[CR95] Gericke B, Amiri M, Scott CR, Naim HY (2017). Molecular pathogenicity of novel sucrase-isomaltase mutations found in congenital sucrase-isomaltase deficiency patients. Biochimica Et Biophysica Acta (BBA) - Molecular Basis of Disease.

[CR96] Glousker G, Touzot F, Revy P (2015). Unraveling the pathogenesis of Hoyeraal-Hreidarsson syndrome, a complex telomere biology disorder. Br J Haematol.

[CR97] Gluchowski NL, Chitraju C, Picoraro JA (2017). Identification and characterization of a novel DGAT1 missense mutation associated with congenital diarrhea. J Lipid Res.

[CR98] Goulet O, Salomon J, Ruemmele F (2007). Intestinal epithelial dysplasia (tufting enteropathy). Orphanet J Rare Dis.

[CR99] Gradwohl G, Dierich A, LeMeur M, Guillemot F (2000). neurogenin3 is required for the development of the four endocrine cell lineages of the pancreas. Proc Natl Acad Sci USA.

[CR100] Gramatges MM, Bertuch AA (2013). Short telomeres: from dyskeratosis congenita to sporadic aplastic anemia and malignancy. Transl Res.

[CR101] Griffin G, Shenoi S, Hughes GC (2020). Hemophagocytic lymphohistiocytosis: an update on pathogenesis, diagnosis, and therapy. Best Pract Res Clin Rheumatol.

[CR102] Haas JT, Winter HS, Lim E (2012). DGAT1 mutation is linked to a congenital diarrheal disorder. J Clin Invest.

[CR103] Hadorn B, Tarlow MJ, Lloyd JK, Wolff OH (1969). Intestinal enterokinase deficiency. Lancet.

[CR104] Hartley JL, Zachos NC, Dawood B (2010). Mutations in TTC37 cause trichohepatoenteric syndrome (phenotypic diarrhea of infancy). Gastroenterology.

[CR105] Haworth JC, Gourley B, Hadorn B, Sumida C (1971). Malabsorption and growth failure due to intestinal enterokinase deficiency. J Pediatr.

[CR106] Hayashi S, Yokoi T, Hatano C (2018). Biallelic mutations of EGFR in a compound heterozygous state cause ectodermal dysplasia with severe skin defects and gastrointestinal dysfunction. Hum Genome Var.

[CR107] He J, Navarrete S, Jasinski M (2002). Targeted disruption of Dkc1, the gene mutated in X-linked dyskeratosis congenita, causes embryonic lethality in mice. Oncogene.

[CR108] Heinz-Erian P, Müller T, Krabichler B (2009). Mutations in SPINT2 cause a syndromic form of congenital sodium diarrhea. Am J Hum Genet.

[CR109] Henter J-I, Horne A, Aricó M (2007). HLH-2004: Diagnostic and therapeutic guidelines for hemophagocytic lymphohistiocytosis. Pediatr Blood Cancer.

[CR110] Hickey JW, Neumann EK, Radtke AJ (2022). Spatial mapping of protein composition and tissue organization: a primer for multiplexed antibody-based imaging. Nat Methods.

[CR111] Hirabayashi KE, Moore AT, Mendelsohn BA (2018). Congenital sodium diarrhea and chorioretinal coloboma with optic disc coloboma in a patient with biallelic SPINT2 mutations, including p. (Tyr163Cys). Am J Med Genet A.

[CR112] Hirsh V, Blais N, Burkes R (2014). Management of diarrhea induced by epidermal growth factor receptor tyrosine kinase inhibitors. Curr Oncol.

[CR113] Höck M, Wegleiter K, Ralser E (2015). ALG8-CDG: novel patients and review of the literature. Orphanet J Rare Dis.

[CR114] Höglund P, Haila S, Socha J (1996). Mutations of the Down-regulated in adenoma (DRA) gene cause congenital chloride diarrhoea. Nat Genet.

[CR115] Höglund P, Auranen M, Socha J (1998). Genetic background of congenital chloride diarrhea in high-incidence populations: Finland, Poland, and Saudi Arabia and Kuwait. Am J Hum Genetics.

[CR116] Höglund P, Holmberg C, Sherman P, Kere J (2001). Distinct outcomes of chloride diarrhoea in two siblings with identical genetic background of the disease: implications for early diagnosis and treatment. Gut.

[CR117] Holm IA, Agrawal PB, Ceyhan-Birsoy O (2018). The BabySeq project: implementing genomic sequencing in newborns. BMC Pediatr.

[CR118] Holmberg C, Perheentupa J (1985). Congenital Na+ diarrhea: a new type of secretory diarrhea. J Pediatr.

[CR119] Holmberg C, Perheentupa J, Launiala K (1975). Colonic electrolyte transport in health and in congenital chloride diarrhea. J Clin Invest.

[CR120] Holmberg C, Perheentupa J, Launiala K, Hallman N (1977). Congenital chloride diarrhoea. Clinical analysis of 21 Finnish patients. Arch Dis Child.

[CR121] Holt-Danborg L, Vodopiutz J, Nonboe AW (2019). SPINT2 (HAI-2) missense variants identified in congenital sodium diarrhea/tufting enteropathy affect the ability of HAI-2 to inhibit prostasin but not matriptase. Hum Mol Genet.

[CR122] Holzinger A, Maier EM, Bück C (2002). Mutations in the proenteropeptidase gene are the molecular cause of congenital enteropeptidase deficiency. Am J Hum Genet.

[CR123] Hooper AJ, van Bockxmeer FM, Burnett JR (2005). Monogenic hypocholesterolaemic lipid disorders and apolipoprotein B metabolism. Crit Rev Clin Lab Sci.

[CR124] Hsiao P-J, Lee M-Y, Wang Y-T (2015). MTTP-297H polymorphism reduced serum cholesterol but increased risk of non-alcoholic fatty liver disease-a cross-sectional study. BMC Med Genet.

[CR125] Jach D, Cheng Y, Prica F (2021). From development to cancer—an ever-increasing role of AGR2. Am J Cancer Res.

[CR126] Jackson RS, Creemers JWM, Farooqi IS (2003). Small-intestinal dysfunction accompanies the complex endocrinopathy of human proprotein convertase 1 deficiency. J Clin Invest.

[CR127] Jacob R, Peters K, Naim HY (2002). The prosequence of human lactase-phlorizin hydrolase modulates the folding of the mature enzyme. J Biol Chem.

[CR128] Jaeken J, Matthijs G, Saudubray JM (1998). Phosphomannose isomerase deficiency: a carbohydrate-deficient glycoprotein syndrome with hepatic-intestinal presentation. Am J Hum Genet.

[CR129] Janecke AR, Heinz-Erian P, Yin J (2015). Reduced sodium/proton exchanger NHE3 activity causes congenital sodium diarrhea. Hum Mol Genet.

[CR130] Janecke AR, Heinz-Erian P, Müller T (2016). Congenital sodium diarrhea: a form of intractable diarrhea, with a link to inflammatory bowel disease. J Pediatr Gastroenterol Nutr.

[CR131] Janecke AR, Liu X, Adam R (2021). Pathogenic STX3 variants affecting the retinal and intestinal transcripts cause an early-onset severe retinal dystrophy in microvillus inclusion disease subjects. Hum Genet.

[CR132] Jardine S, Dhingani N, Muise AM (2018). TTC7A: Steward of Intestinal Health. Cell Mol Gastroenterol Hepatol.

[CR133] Jardine S, Anderson S, Babcock S (2020). Drug screen identifies leflunomide for treatment of inflammatory bowel disease caused by TTC7A deficiency. Gastroenterology.

[CR134] Järvelä I, Enattah NS, Kokkonen J (1998). Assignment of the locus for congenital lactase deficiency to 2q21, in the vicinity of but separate from the lactase-phlorizin hydrolase gene. Am J Hum Genet.

[CR135] Jean F, Basak A, Rondeau N (1993). Enzymic characterization of murine and human prohormone convertase-1 (mPC1 and hPC1) expressed in mammalian GH4C1 cells. Biochem J.

[CR136] Jensen J (2004). Gene regulatory factors in pancreatic development. Dev Dyn.

[CR137] Jonassaint NL, Guo N, Califano JA (2013). The gastrointestinal manifestations of telomere-mediated disease. Aging Cell.

[CR138] Jones B, Jones EL, Bonney SA (2003). Mutations in a Sar1 GTPase of COPII vesicles are associated with lipid absorption disorders. Nat Genet.

[CR139] Jostins L, Ripke S, Weersma RK (2012). Host-microbe interactions have shaped the genetic architecture of inflammatory bowel disease. Nature.

[CR140] Kaji I, Roland JT, Watanabe M (2020). Lysophosphatidic acid increases maturation of brush borders and SGLT1 activity in MYO5B-deficient mice, a model of microvillus inclusion disease. Gastroenterology.

[CR141] Kaji I, Roland JT, Rathan-Kumar S (2021). Cell differentiation is disrupted by MYO5B loss through Wnt/Notch imbalance. JCI Insight.

[CR142] Kambal MA, Al-Harbi DA, Al-Sunaid AR, Al-Atawi MS (2019). Mitchell-Riley syndrome due to a novel mutation in RFX6. Front Pediatr.

[CR143] Kammermeier J, Drury S, James CT (2014). Targeted gene panel sequencing in children with very early onset inflammatory bowel disease–evaluation and prospective analysis. J Med Genet.

[CR144] Kammermeier J, Lucchini G, Pai S-Y (2016). Stem cell transplantation for tetratricopeptide repeat domain 7A deficiency: long-term follow-up. Blood.

[CR145] Kaplanis J, Samocha KE, Wiel L (2020). Evidence for 28 genetic disorders discovered by combining healthcare and research data. Nature.

[CR146] Kato M, Das S, Petras K (2004). Mutations of ARX are associated with striking pleiotropy and consistent genotype-phenotype correlation. Hum Mutat.

[CR147] Katoh M, Hirai M, Sugimura T, Terada M (1996). Cloning, expression and chromosomal localization of Wnt-13, a novel member of the Wnt gene family. Oncogene.

[CR148] Kawaguchi M, Yamamoto K, Takeda N (2019). Hepatocyte growth factor activator inhibitor-2 stabilizes Epcam and maintains epithelial organization in the mouse intestine. Commun Biol.

[CR149] Kelly CJ, Zheng L, Campbell EL (2015). Crosstalk between microbiota-derived short-chain fatty acids and intestinal epithelial HIF augments tissue barrier function. Cell Host Microbe.

[CR150] Kelsey WM (1954). Congenital alkalosis with diarrhea. AMA Am J Dis Child.

[CR151] Kiela PR, Ghishan FK (2016). Physiology of intestinal absorption and secretion. Best Pract Res Clin Gastroenterol.

[CR152] Kim J, Hu C, Moufawad El Achkar C (2019). Patient-customized oligonucleotide therapy for a rare genetic disease. N Engl J Med.

[CR153] Kini A, Zhao B, Basic M (2022). Upregulation of antimicrobial peptide expression in slc26a3-/- mice with colonic dysbiosis and barrier defect. Gut Microbes.

[CR154] Kitamura K, Yanazawa M, Sugiyama N (2002). Mutation of ARX causes abnormal development of forebrain and testes in mice and X-linked lissencephaly with abnormal genitalia in humans. Nat Genet.

[CR155] Klee KMC, Janecke AR, Civan HA (2020). AP1S1 missense mutations cause a congenital enteropathy via an epithelial barrier defect. Hum Genet.

[CR156] Kloor M, Voigt AY, Schackert HK (2011). Analysis of EPCAM protein expression in diagnostics of Lynch syndrome. J Clin Oncol.

[CR157] Knight S, Vulliamy T, Copplestone A (1998). Dyskeratosis Congenita (DC) Registry: identification of new features of DC. Br J Haematol.

[CR158] Knowles BC, Roland JT, Krishnan M (2014). Myosin Vb uncoupling from RAB8A and RAB11A elicits microvillus inclusion disease. J Clin Invest.

[CR159] Konishi K, Mizuochi T, Yanagi T (2019). Clinical features, molecular genetics, and long-term outcome in congenital chloride diarrhea: a Nationwide Study in Japan. J Pediatr.

[CR160] Kota P, García-Caballero A, Dang H (2012). Energetic and structural basis for activation of the epithelial sodium channel by matriptase. Biochemistry.

[CR161] Kozan PA, McGeough MD, Peña CA (2015). Mutation of EpCAM leads to intestinal barrier and ion transport dysfunction. J Mol Med.

[CR162] Kristal E, Nahum A, Ling G (2022). Hyper IgM in tricho-hepato-enteric syndrome due to TTC37 mutation. Immunol Res.

[CR163] Kuokkanen M, Kokkonen J, Enattah NS (2006). Mutations in the translated region of the lactase gene (LCT) underlie congenital lactase deficiency. Am J Hum Genet.

[CR164] Landers (2003) Intractable diarrhea of infancy with facial dysmorphism, trichorrhexis nodosa, and cirrhosis. In: Pediatric dermatology 10.1046/j.1525-1470.2003.20514.x 14521564

[CR165] LeBlanc C, Schlegel C, Robinson JR (2020). The financial burden of an undiagnosed congenital diarrhea disorder. J Pediatr Gastroenterol Nutr.

[CR166] Lee J, Hegele RA (2014). Abetalipoproteinemia and homozygous hypobetalipoproteinemia: a framework for diagnosis and management. J Inherit Metab Dis.

[CR167] Leng C, Rings EHHM, de Wildt SN, van IJzendoorn SCD, (2020). Pharmacological and parenteral nutrition-based interventions in microvillus inclusion disease. J Clin Med.

[CR168] Leppert M, Breslow JL, Wu L (1988). Inference of a molecular defect of apolipoprotein B in hypobetalipoproteinemia by linkage analysis in a large kindred. J Clin Invest.

[CR169] Levitt DG, Levitt MD (2017). Protein losing enteropathy: comprehensive review of the mechanistic association with clinical and subclinical disease states. Clin Exp Gastroenterol.

[CR170] Li HJ, Kapoor A, Giel-Moloney M (2012). Notch signaling differentially regulates the cell fate of early endocrine precursor cells and their maturing descendants in the mouse pancreas and intestine. Dev Biol.

[CR171] Li Q, Zhou Z, Sun Y (2022). A functional relationship between UNC45A and MYO5B connects two rare diseases with shared enteropathy. Cell Mol Gastroenterol Hepatol.

[CR172] Lien R, Lin Y-F, Lai M-W (2017). Novel mutations of the tetratricopeptide repeat domain 7A gene and phenotype/genotype comparison. Front Immunol.

[CR173] Lipiński P, Tylki-Szymańska A (2021). Congenital disorders of glycosylation: what clinicians need to know?. Front Pediatr.

[CR174] Liu B, He Y, Wang Y (2022). Structure of active human telomerase with telomere shelterin protein TPP1. Nature.

[CR175] Luaniala K, Perheentupa J, Pasternack A, Hallman N (1968) Familial chloride diarrhea-chloride malabsorption. In: Modern Problems in Pediatrics. Karger, Basel, p 137 4887367

[CR176] Lynch HT, Riegert-Johnson DL, Snyder C (2011). Lynch syndrome-associated extracolonic tumors are rare in two extended families with the same EPCAM deletion. Am J Gastroenterol.

[CR177] Madhusudan M, Sankaranarayanan S, Ravikumar T (2021). Enterokinase deficiency: a case of pancreatic insufficiency. Indian J Pediatr.

[CR178] Mantoo MR, Malik R, Das P (2021). Congenital diarrhea and enteropathies in infants: approach to diagnosis. Indian J Pediatr.

[CR179] Martín MG, Turk E, Lostao MP (1996). Defects in Na+/glucose cotransporter (SGLT1) trafficking and function cause glucose-galactose malabsorption. Nat Genet.

[CR180] Martín MG, Lindberg I, Solorzano-Vargas RS (2013). Congenital proprotein convertase 1/3 deficiency causes malabsorptive diarrhea and other endocrinopathies in a pediatric cohort. Gastroenterology.

[CR181] Martinelli D, Travaglini L, Drouin CA (2013). MEDNIK syndrome: a novel defect of copper metabolism treatable by zinc acetate therapy. Brain.

[CR182] Mason PJ, Bessler M (2011). The genetics of dyskeratosis congenita. Cancer Genet.

[CR183] Matsunoshita N, Nozu K, Yoshikane M (2018). Congenital chloride diarrhea needs to be distinguished from Bartter and Gitelman syndrome. J Hum Genet.

[CR184] Mazelova J, Ransom N, Astuto-Gribble L (2009). Syntaxin 3 and SNAP-25 pairing, regulated by omega-3 docosahexaenoic acid, controls the delivery of rhodopsin for the biogenesis of cilia-derived sensory organelles, the rod outer segments. J Cell Sci.

[CR185] Mazurova S, Tesarova M, Zeman J (2020). Fatal neonatal nephrocutaneous syndrome in 18 Roma children with EGFR deficiency. J Dermatol.

[CR186] Mendes HF, van der Spuy J, Chapple JP, Cheetham ME (2005). Mechanisms of cell death in rhodopsin retinitis pigmentosa: implications for therapy. Trends Mol Med.

[CR187] Miller SA, Burnett JR, Leonis MA (2014). Novel missense MTTP gene mutations causing abetalipoproteinemia. Biochim Biophys Acta.

[CR188] Mitchell J, Punthakee Z, Lo B (2004). Neonatal diabetes, with hypoplastic pancreas, intestinal atresia and gall bladder hypoplasia: search for the aetiology of a new autosomal recessive syndrome. Diabetologia.

[CR189] Moffitt JR, Hao J, Bambah-Mukku D (2016). High-performance multiplexed fluorescence in situ hybridization in culture and tissue with matrix imprinting and clearing. Proc Natl Acad Sci USA.

[CR190] Moidu NA, Rahman NS, Syafruddin SE (2020). Secretion of pro-oncogenic AGR2 protein in cancer. Heliyon.

[CR191] Montpetit A, Côté S, Brustein E (2008). Disruption of AP1S1, causing a novel neurocutaneous syndrome, perturbs development of the skin and spinal cord. PLoS Genet.

[CR192] Mössner J (2010). New advances in cell physiology and pathophysiology of the exocrine pancreas. Dig Dis.

[CR193] Müller T, Wijmenga C, Phillips AD (2000). Congenital sodium diarrhea is an autosomal recessive disorder of sodium/proton exchange but unrelated to known candidate genes. Gastroenterology.

[CR194] Müller T, Hess MW, Schiefermeier N (2008). MYO5B mutations cause microvillus inclusion disease and disrupt epithelial cell polarity. Nat Genet.

[CR195] Müller T, Rasool I, Heinz-Erian P (2016). Congenital secretory diarrhoea caused by activating germline mutations in *GUCY2C*. Gut.

[CR196] Muller L, Lindberg I (1999). The cell biology of the prohormone convertases PC1 and PC2. Prog Nucleic Acid Res Mol Biol.

[CR197] Naim HY, Roth J, Sterchi EE (1988). Sucrase-isomaltase deficiency in humans. Different mutations disrupt intracellular transport, processing, and function of an intestinal brush border enzyme. J Clin Invest.

[CR198] Nambu R, Muise AM (2020). Advanced understanding of monogenic inflammatory bowel disease. Front Pediatr.

[CR199] Niehues R, Hasilik M, Alton G (1998). Carbohydrate-deficient glycoprotein syndrome type Ib. Phosphomannose isomerase deficiency and mannose therapy. J Clin Invest.

[CR200] Nóbrega S, Monteiro MP, Pereira-da-Silva L (2021). Congenital glucagon-like peptide-1 deficiency in the pathogenesis of protracted diarrhea in Mitchell-Riley syndrome. J Clin Endocrinol Metab.

[CR201] Nwia SM, Li XC, de Leite APO (2022). The Na+/H+ exchanger 3 in the intestines and the proximal tubule of the kidney: localization, physiological function, and key roles in angiotensin II-induced hypertension. Front Physiol.

[CR202] O’Connell AE, Zhou F, Shah MS (2018). Neonatal-onset chronic diarrhea caused by homozygous nonsense WNT2B mutations. Am J Hum Genet.

[CR203] O’Sullivan MJ, Lindsay AJ (2020). The endosomal recycling pathway—at the crossroads of the cell. Int J Mol Sci.

[CR204] Oberemok VV, Laikova KV, Repetskaya AI (2018). A half-century history of applications of antisense oligonucleotides in medicine, agriculture and forestry: we should continue the journey. Molecules.

[CR205] Ozler O, Brunner-Véber A, Fatih P (2021). Long-term follow-up of tufting enteropathy caused by EPCAM mutation p.Asp253Asn and absent EPCAM expression. JPGN Reports.

[CR206] Oz-Levi D, Olender T, Bar-Joseph I (2019). Noncoding deletions reveal a gene that is critical for intestinal function. Nature.

[CR207] Pagel J, Beutel K, Lehmberg K (2012). Distinct mutations in STXBP2 are associated with variable clinical presentations in patients with familial hemophagocytic lymphohistiocytosis type 5 (FHL5). Blood.

[CR208] Park S-W, Zhen G, Verhaeghe C (2009). The protein disulfide isomerase AGR2 is essential for production of intestinal mucus. Proc Natl Acad Sci USA.

[CR209] Pathak SJ, Mueller JL, Okamoto K (2019). EPCAM mutation update: Variants associated with congenital tufting enteropathy and Lynch syndrome. Hum Mutat.

[CR210] Peotter J, Kasberg W, Pustova I, Audhya A (2019). COPII-mediated trafficking at the ER/ERGIC interface. Traffic.

[CR211] Peretti N, Mas E (2022). Congenital disorders of intestinal digestion and absorption (sugars, proteins, lipids, ions). Best Pract Res Clin Gastroenterol.

[CR212] Perheentupa J, Eklund J, Hallman N (1965). Chronic diarrhea and alkalosis. Pediatrics.

[CR213] Perry A, Bensallah H, Martinez-Vinson C (2014). Microvillous atrophy: atypical presentations. J Pediatr Gastroenterol Nutr.

[CR214] Poulton C, Pathak G, Mina K (2019). Tricho-hepatic-enteric syndrome (THES) without intractable diarrhoea. Gene.

[CR215] Puntis JWL, Zamvar V (2015). Congenital sucrase–isomaltase deficiency: diagnostic challenges and response to enzyme replacement therapy. Arch Dis Child.

[CR216] Qiu Y, Gong J, Feng J (2017). Defects in myosin VB are associated with a spectrum of previously undiagnosed low γ-glutamyltransferase cholestasis. Hepatology.

[CR217] Reifen RM, Cutz E, Griffiths AM (1994). Tufting enteropathy: a newly recognized clinicopathological entity associated with refractory diarrhea in infants. J Pediatr Gastroenterol Nutr.

[CR218] Remec ZI, TrebusakPodkrajsek K, Repic Lampret B (2021). Next-generation sequencing in newborn screening: a review of current state. Front Genet.

[CR219] Rider NL, Boisson B, Jyonouchi S (2015). Novel TTC37 mutations in a patient with immunodeficiency without diarrhea: extending the phenotype of trichohepatoenteric syndrome. Front Pediatr.

[CR220] Rockowitz S, LeCompte N, Carmack M (2020). Children’s rare disease cohorts: an integrative research and clinical genomics initiative. NPJ Genom Med.

[CR221] Rubio-Cabezas O, Jensen JN, Hodgson MI (2011). Permanent neonatal diabetes and enteric anendocrinosis associated with biallelic mutations in *NEUROG3*. Diabetes.

[CR222] Ruemmele FM, Schmitz J, Goulet O (2006). Microvillous inclusion disease (microvillous atrophy). Orphanet J Rare Dis.

[CR223] Rugo HS, Di Palma JA, Tripathy D (2019). The characterization, management, and future considerations for ErbB-family TKI-associated diarrhea. Breast Cancer Res Treat.

[CR224] Russo P (2020). Updates in pediatric congenital enteropathies. Surg Pathol Clin.

[CR225] Saba TG, Montpetit A, Verner A (2005). An atypical form of erythrokeratodermia variabilis maps to chromosome 7q22. Hum Genet.

[CR226] Sala Coromina J, VinaixaVergés A, Garcia Puig R (2015). Congenital lactase deficiency: identification of a new mutation. An Pediatr.

[CR227] Salomon J, Goulet O, Canioni D (2014). Genetic characterization of congenital tufting enteropathy: epcam associated phenotype and involvement of SPINT2 in the syndromic form. Hum Genet.

[CR228] Samuels ME, Majewski J, Alirezaie N (2013). Exome sequencing identifies mutations in the gene TTC7A in French-Canadian cases with hereditary multiple intestinal atresia. J Med Genet.

[CR229] Sanchez-Pulido L, Jia S, Hansen CG, Ponting CP (2022). PERCC1, a new member of the Yap/TAZ/FAM181 transcriptional co-regulator family. Bioinformatics Advances.

[CR230] Santos AJM, Lo Y-H, Mah AT, Kuo CJ (2018). The intestinal stem cell niche: homeostasis and adaptations. Trends Cell Biol.

[CR231] Sato T, Mushiake S, Kato Y (2007). The Rab8 GTPase regulates apical protein localization in intestinal cells. Nature.

[CR232] Savage SA, Niewisch MR (2022). Dyskeratosis congenita and related telomere biology disorders.

[CR233] Scheepers A, Joost H-G, Schürmann A (2004). The glucose transporter families SGLT and GLUT: molecular basis of normal and aberrant function. JPEN J Parenter Enteral Nutr.

[CR234] Scheufler C, Brinker A, Bourenkov G (2000). Structure of TPR domain-peptide complexes: critical elements in the assembly of the Hsp70-Hsp90 multichaperone machine. Cell.

[CR235] Schollen E, Dorland L, de Koning TJ (2000). Genomic organization of the human phosphomannose isomerase (MPI) gene and mutation analysis in patients with congenital disorders of glycosylation type Ib (CDG-Ib). Hum Mutat.

[CR236] Sehgal R, Sheahan K, O’Connell PR (2014). Lynch syndrome: an updated review. Genes.

[CR237] Sekne Z, Ghanim GE, van Roon A-MM, Nguyen THD (2022). Structural basis of human telomerase recruitment by TPP1-POT1. Science.

[CR238] Shao X-X, Lin D-P, Sun L (2018). Association of ulcerative colitis with solute-linked carrier family 26 member A3 gene polymorphisms and its expression in colonic tissues in Chinese patients. Int J Colorectal Dis.

[CR239] Shoubridge C, Fullston T, Gécz J (2010). ARX spectrum disorders: making inroads into the molecular pathology. Hum Mutat.

[CR240] Sivagnanam M, Mueller JL, Lee H (2008). Identification of EpCAM as the gene for congenital tufting enteropathy. Gastroenterology.

[CR241] Sivagnanam M, Janecke AR, Müller T (2010). Case of syndromic tufting enteropathy harbors SPINT2 mutation seen in congenital sodium diarrhea. Clin Dysmorphol.

[CR242] Siwak DR, Carey M, Hennessy BT (2010). Targeting the epidermal growth factor receptor in epithelial ovarian cancer: current knowledge and future challenges. J Oncol.

[CR243] Smith SB, Qu H-Q, Taleb N (2010). Rfx6 directs islet formation and insulin production in mice and humans. Nature.

[CR244] Sobajima T, Yoshimura S-I, Iwano T (2014). Rab11a is required for apical protein localisation in the intestine. Biol Open.

[CR245] Stepensky P, Bartram J, Barth TF (2013). Persistent defective membrane trafficking in epithelial cells of patients with familial hemophagocytic lymphohistiocytosis type 5 due to STXBP2/MUNC18-2 mutations. Pediatr Blood Cancer.

[CR246] Stephen J, Vilboux T, Haberman Y (2016). Congenital protein losing enteropathy: an inborn error of lipid metabolism due to DGAT1 mutations. Eur J Hum Genet.

[CR247] Stijnen P, Ramos-Molina B, O’Rahilly S, Creemers JWM (2016). PCSK1 mutations and human endocrinopathies: from obesity to gastrointestinal disorders. Endocr Rev.

[CR248] Stölting T, Omran H, Erlekotte A (2009). Novel ALG8 mutations expand the clinical spectrum of congenital disorder of glycosylation type Ih. Mol Genet Metab.

[CR249] Strømme P, Mangelsdorf ME, Shaw MA (2002). Mutations in the human ortholog of Aristaless cause X-linked mental retardation and epilepsy. Nat Genet.

[CR250] Suda Y, Kurokawa K, Nakano A (2017). Regulation of ER-Golgi transport dynamics by GTPases in budding yeast. Front Cell Dev Biol.

[CR251] Sui X, Wang K, Gluchowski NL (2020). Structure and catalytic mechanism of a human triacylglycerol-synthesis enzyme. Nature.

[CR252] Syed S, Al-Boni M, Khan MN (2019). Assessment of machine learning detection of environmental enteropathy and celiac disease in children. JAMA Netw Open.

[CR253] Sznajer Y, Baumann C, David A (2003). Further delineation of the congenital form of X-linked dyskeratosis congenita (Hoyeraal-Hreidarsson syndrome). Eur J Pediatr.

[CR254] Terry NA, Lee RA, Walp ER (2015). Dysgenesis of enteroendocrine cells in Aristaless-Related Homeobox polyalanine expansion mutations. J Pediatr Gastroenterol Nutr.

[CR255] Thiagarajah JR, Kamin DS, Acra S (2018). Advances in evaluation of chronic diarrhea in infants. Gastroenterology.

[CR256] Torniainen S, Freddara R, Routi T (2009). Four novel mutations in the lactase gene (LCT) underlying congenital lactase deficiency (CLD). BMC Gastroenterol.

[CR257] Torres C, Badalyan V, Mohan P (2019) P4.26: Outcomes of three genetic syndromes with intestinal failure (IF), short bowel syndrome (SBS), intestinal failure associated liver disease (IFALD) and parenteral nutrition (PN) dependency, treated in our Intestinal Rehabilitation Program (IRP). Children’s National Medical Center Washington DC. Transplantation 103:S154. 10.1097/01.tp.0000576368.39404.86

[CR258] Treem WR (2012). Clinical aspects and treatment of congenital sucrase-isomaltase deficiency. J Pediatr Gastroenterol Nutr.

[CR259] Troelsen JT (2005). Adult-type hypolactasia and regulation of lactase expression. Biochim Biophys Acta.

[CR260] van der Flier LG, Haegebarth A, Stange DE (2009). OLFM4 is a robust marker for stem cells in human intestine and marks a subset of colorectal cancer cells. Gastroenterology.

[CR261] van der Velde KJ, Dhekne HS, Swertz MA (2013). An overview and online registry of microvillus inclusion disease patients and their MYO5B mutations. Hum Mutat.

[CR262] van Rijn JM, Ardy RC, Kuloğlu Z (2018). Intestinal failure and aberrant lipid metabolism in patients with DGAT1 deficiency. Gastroenterology.

[CR263] van Rijn JM, van Hoesel M, de Heus C (2019). DGAT2 partially compensates for lipid-induced ER stress in human DGAT1-deficient intestinal stem cells. J Lipid Res.

[CR264] van Vugt AHM, Bijvelds MJC, De Jonge HR, et al (2021) A potential treatment of congenital sodium diarrhea in patients with activating GUCY2C mutations. Clin Transl Gastroenterol 12:427. 10.14309/ctg.0000000000000427 10.14309/ctg.0000000000000427 PMC8604003 34797252

[CR297] Vlasschaert C, McIntyre AD, Thomson LA, Kennedy BA, Ratko S, Prasad C, Hegele RA (2021) Abetalipoproteinemia due to a novel splicing variant in MTTP in 3 siblings. J Investig Med High Impact Case Rep 9:9232470962110224. 10.1177/23247096211022484 10.1177/23247096211022484 PMC8182224 34078172

[CR265] Vogel GF, Klee KMC, Janecke AR (2015). Cargo-selective apical exocytosis in epithelial cells is conducted by Myo5B, Slp4a, Vamp7, and Syntaxin 3. J Cell Biol.

[CR266] Wakeman JA, Walsh J, Andrews PW (1998). Human Wnt-13 is developmentally regulated during the differentiation of NTERA-2 pluripotent human embryonal carcinoma cells. Oncogene.

[CR267] Wakida N, Kitamura K, Tuyen DG (2006). Inhibition of prostasin-induced ENaC activities by PN-1 and regulation of PN-1 expression by TGF-beta1 and aldosterone. Kidney Int.

[CR268] Wanes D, Husein DM, Naim HY (2019). Congenital lactase deficiency: mutations, functional and biochemical implications, and future perspectives. Nutrients.

[CR269] Wang J, Cortina G, Wu SV (2006). Mutant neurogenin-3 in congenital malabsorptive diarrhea. N Engl J Med.

[CR270] Wang AL, Knight DK, Vu T-TT, Mehta MC (2019). Retinitis pigmentosa: review of current treatment. Int Ophthalmol Clin.

[CR271] Wang L, Qian H, Nian Y (2020). Structure and mechanism of human diacylglycerol O-acyltransferase 1. Nature.

[CR272] Wang L, Zhang D, Fan C (2020). Novel compound heterozygous TMPRSS15 gene variants cause enterokinase deficiency. Front Genet.

[CR273] Wedenoja S, Hã-Glund P, Holmberg C (2010). Review article: the clinical management of congenital chloride diarrhoea. Aliment Pharmacol Ther.

[CR274] Wedenoja S, Pekansaari E, Höglund P (2011). Update on SLC26A3 mutations in congenital chloride diarrhea. Hum Mutat.

[CR275] Weijers HA, va de Kamer JH, Mossel DA, Dicke WK (1960). Diarrhoea caused by deficiency of sugar-splitting enzymes. Lancet.

[CR276] Welty FK (2014). Hypobetalipoproteinemia and Abetalipoproteinemia. Curr Opin Lipidol.

[CR277] Wetterstrand K (2021) DNA Sequencing costs: data from the NHGRI Genome Sequencing Program (GSP). Accessed 19 Aug 2022

[CR278] Wiegerinck CL, Janecke AR, Schneeberger K (2014). Loss of syntaxin 3 causes variant microvillus inclusion disease. Gastroenterology.

[CR279] Wilschanski M, Abbasi M, Blanco E (2014). A novel familial mutation in the PCSK1 gene that alters the oxyanion hole residue of proprotein convertase 1/3 and impairs its enzymatic activity. PLoS ONE.

[CR280] Woo D-H, Chen Q, Yang T-LB (2016). Enhancing a Wnt-telomere feedback loop restores intestinal stem cell function in a human organotypic model of dyskeratosis congenita. Cell Stem Cell.

[CR281] Wright EM, Turk E, Martin MG (2002). Molecular basis for glucose-galactose malabsorption. Cell Biochem Biophys.

[CR282] Wright EM, Loo DDF, Hirayama BA (2011). Biology of human sodium glucose transporters. Physiol Rev.

[CR283] Wright CF, McRae JF, Clayton S (2018). Making new genetic diagnoses with old data: iterative reanalysis and reporting from genome-wide data in 1,133 families with developmental disorders. Genet Med.

[CR284] Wrobel C, Zafeiriou M-P, Moser T (2021). Understanding and treating paediatric hearing impairment. EBioMedicine.

[CR285] Wu C-J, Feng X, Lu M (2017). Matriptase-mediated cleavage of EpCAM destabilizes claudins and dysregulates intestinal epithelial homeostasis. J Clin Invest.

[CR286] Xiao F, Juric M, Li J (2012). Loss of downregulated in adenoma (DRA) impairs mucosal HCO3(-) secretion in murine ileocolonic inflammation. Inflamm Bowel Dis.

[CR287] Xu L, Gu W, Luo Y (2020). DGAT1 mutations leading to delayed chronic diarrhoea: a case report. BMC Med Genet.

[CR288] Xue J, Thomas L, Tahmasbi M (2020). An inducible intestinal epithelial cell-specific NHE3 knockout mouse model mimicking congenital sodium diarrhea. Clin Sci.

[CR289] Younis M, Rastogi R, Chugh A (2020). Congenital diarrheal diseases. Clin Perinatol.

[CR290] Yu Q (2021). Slc26a3 (DRA) in the gut: expression, function, regulation, role in infectious diarrhea and inflammatory bowel disease. Inflamm Bowel Dis.

[CR291] Yu M, Wei Y, Xu K (2019). EGFR deficiency leads to impaired self-renewal and pluripotency of mouse embryonic stem cells. PeerJ.

[CR292] Zamel R, Khan R, Pollex RL, Hegele RA (2008). Abetalipoproteinemia: two case reports and literature review. Orphanet J Rare Dis.

[CR293] Zeve D, Stas E, de Sousa CJ (2022). Robust differentiation of human enteroendocrine cells from intestinal stem cells. Nat Commun.

[CR294] Zhang K, Chandrakasan S, Chapman H (2014). Synergistic defects of different molecules in the cytotoxic pathway lead to clinical familial hemophagocytic lymphohistiocytosis. Blood.

[CR295] Zhang YJ, Jimenez L, Azova S (2021). Novel variants in the stem cell niche factor WNT2B define the disease phenotype as a congenital enteropathy with ocular dysgenesis. Eur J Hum Genet.

[CR296] zur Stadt U, Rohr J, Seifert W (2009). Familial hemophagocytic lymphohistiocytosis type 5 (FHL-5) is caused by mutations in Munc18-2 and impaired binding to syntaxin 11. Am J Hum Genet.

