# Supplementary material for: The genetics of monogenic intestinal epithelial disorders
Source: Hum Genet. 2022 Nov 23;142(5):613–54. doi: 10.1007/s00439-022-02501-5 (PMC10182130; doi:10.1007/s00439-022-02501-5)
Supplement: Supplementary file 1 — (DOCX 22 KB) [file 439_2022_2501_MOESM1_ESM.docx]

**Table S1. Baseline characteristics of the study population according to the occurrence of delivery at <37 weeks of gestation.**

| **Variable** | **Delivery ≥37 weeks**  **(n=88)** | **Delivery <37 weeks**  **(n=27)** |
| --- | --- | --- |
| Median maternal age (years, IQR) | 30 (25-33) | 33 (27-36) |
| Median pre-pregnancy BMI (kg/m^2^, IQR) | 22.6 (20.0-25.4) | 23.4 (21.9-28.0) |
| Smoking status, n (%) | 15 (17%) | 3 (11%) |
| FGR in previous pregnancy, n (%) | 7 (8.0%) | 2 (7.4%) |
| SGA in previous pregnancy, n (%) | 3 (3.4%) | 0 (0%) |
| Stillbirth in previous pregnancy, n (%) | 0 (0%) | 0 (0%) |
| Risk factors for placental dysfunction |  |  |
| High |  |  |
| Chronic hypertension, n (%) | 1 (1.1%) | 1 (3.7%) |
| Previous preeclampsia, n (%) | 4 (4.5%) | 0 (0%) |
| Pre-pregnancy diabetes, n (%) | 1 (1.1%) | 0 (0%) |
| Chronic kidney disease, n (%) | 0 (0%) | 0 (0%) |
| SLE, n (%) | 0 (0%) | 0 (0%) |
| Antiphospholipid syndrome, n (%) | 2 (2.3%) | 3 (11%) |
| Moderate |  |  |
| First pregnancy, n (%) | 34 (39%) | 13 (48%) |
| Age ≥ 40 years, n (%) | 7 (8.0%) | 1 (3.7%) |
| BMI ≥ 35 kg/m2, n (%) | 0 (0%) | 0 (0%) |
| Family history of preeclampsia, n (%) | 0 (0%) | 0 (0%) |
| Pregnancy interval >10 years, n (%) | 0 (0%) | 1 (3.7%) |
| High risk for placental dysfunction (NICE), n (%) | 9 (10%) | 4 (15%) |
| Ethnicity |  |  |
| White, n (%) | 71 (81%) | 23 (85%) |
| Black, n (%) | 8 (9.1%) | 1 (3.7%) |
| Asian, n (%) | 1 (1.1%) | 1 (3.7%) |
| Southeast Asian, n (%) | 4 (4.5%) | 2 (7.4%) |
| Other, n (%) | 4 (4.5%) | 0 (0%) |
| Mode of conception |  |  |
| Spontaneous, n (%) | 86 (97.7%) | 24 (88.9%) |
| IVF, n (%) | 2 (2.3%) | 3 (11.1%) |
| Insemination, n (%) | 0 (0%) | 0 (0%) |
| Median GA at diagnosis (weeks, IQR) | 34.71 (34.00- 35.63) | 33.57 (32.64-34.29) |
| Median sFlt-1/PlGF (pg/mL, IQR) | 11 (6-30) | 112 (67-311) |
| UtAPI >95^th^ percentile, n (%) | 14 (16%) | 19 (70%) |
| Abnormal fetal Doppler assessment |  |  |
| UAPI >95^th^ percentile, n (%) | 0 (0%) | 2 (7.4%) |
| CPR<5^th^ percentile, n (%) | 13 (15%) | 12 (44%) |
